# Clinical significance and biological mechanisms of glutathione S-transferase mu gene family in colon adenocarcinoma

**DOI:** 10.1186/s12881-020-01066-2

**Published:** 2020-06-15

**Authors:** Erna Guo, Haotang Wei, Xiwen Liao, Liuyu Wu, Xiaoyun Zeng

**Affiliations:** 1 School of Public Health, Guangxi Medical University, 22 Shuang Yong Road, Nanning, 530021 Guangxi Zhuang Autonomous Region People’s Republic of China; 2 School of International Education, Guangxi Medical University, Nanning, 530021 Guangxi Zhuang Autonomous Region People’s Republic of China; 3 Department of gastrointestinal surgery, The Third Affiliated Hospital of Guangxi Medical University, Nanning, 530031 Guangxi Zhuang Autonomous Region People’s Republic of China; 4 Department of Hepatobiliary Surgery, The First Affiliated Hospital of Guangxi Medical University, Nanning, 530021 Guangxi Zhuang Autonomous Region People’s Republic of China

**Keywords:** GSTM, Prognosis, mRNA, Expression, Colon adenocarcinoma

## Abstract

**Background:**

Colon adenocarcinoma (COAD) is the most common form of colon cancer. The *glutathione S-transferase Mu* (*GSTM*) gene belongs to the *GST* gene family, which functions in cell metabolism and detoxification. The relationship between *GSTM* and COAD and the underlying mechanism remain unknown.

**Methods:**

Data extracted from The Cancer Genome Atlas included mRNA expression and clinical information such as gender, age, and tumor stage. Prognostic values of GSTM genes were identified by survival analysis. Function and mechanism of prognostic GSTM genes were identified by gene set enrichment analysis. A nomogram was used to predict the contribution of risk factors to the outcome of COAD patients.

**Results:**

Low expression of *GSTM1* and *GSTM2* was related to favorable OS (adjusted *P* = 0.006, adjusted HR = 0.559, 95% CI = 0.367–0.849 and adjusted *P* = 0.002, adjusted HR = 0.519, 95% CI = 0.342–0.790, respectively) after adjusting for tumor stage. Enrichment analysis also showed that genes involved were related to cell cycle, metabolism, and detoxification processes, as well as the Wnt signaling and NF-κB pathways.

**Conclusions:**

In conclusion, low expression of *GSTM1* and *GSTM2* were significantly associated with favorable prognosis in COAD. These two genes may serve as potential biomarkers of COAD prognosis.

## Background

Colon adenocarcinoma (COAD) is the most common form of colon cancer. There were 140,250 estimated new cases and 50,630 estimated deaths in 2018, and the five years survival rate is 64.5% as determined by the Surveillance, Epidemiology, and End Results Program (SEER; https://seer.cancer.gov) [1]. Alcohol consumption, smoking, and obesity are risk factors for colorectal cancers [2–4]. Identifying appropriate biomarkers for COAD patients prognosis is important. The *glutathione S-transferase Mu* (*GSTM*) gene family belongs to the GST sub-family, which plays important roles in cell metabolism and detoxification [5–7]. *GSTM* is encoded by five genes (*GSTM1*–*5*) [8–11]. However, the correlation of *GSTM* with the prognosis of cancers is not clear, and there are no reports about the relationship between the *GSTM* family and COAD. In the present study, we investigated the expression of the *GSTM* gene family in COAD, and performed a survival analysis including clinical data. A nomogram model was used to predict the outcome of COAD, and joint-effects survival analysis was carried out to show that low expression of *GSTM1* and *GSTM2* was a sensitive predictor of favorable prognosis. Gene set enrichment analysis (GSEA) and serval enrichment analysis were performed to clarify the potential function and prognostic value of low *GSTM1* and *GSTM2* expression.

## Methods

### Data preparation

The patient’s individual prognosis information was downloaded from University of California, Santa Cruz Xena browser (UCSC Xena: http:// xena.ucsc.edu/, accessed Oct. 5th, 2018). The mRNA expression data for the analysis were generated from The Cancer Genome Atlas (TCGA, http://tcga-data.nci.nih.gov/tcga, accessed Oct. 1th, 2018). Clinical information of 438 patients including gender, age, and tumor stage were selected after deleting cases with missing survival status and survival time of 0 days.

### Bioinformatics analysis

To understand the distribution of GSTM genes between COAD tumor and normal tissues, boxplots were generated from Gene Expression Profiling Interactive Analysis (GEPIA, http://gepia.cancer-pku.cn/, accessed Oct. 2, 2018) [12]. The Database for Annotation, Visualization, and Integrated Discovery (DAVID) v.6.8 (https://david.ncifcrf.gov/tools.jsp, accessed Oct. 11, 2018) [13, 14] and BiNGO (https://www.psb.ugent.be/cbd/papers/BiNGO/Home, accessed Oct.12, 2018) [15] were used to analyze functional enrichment.

### Correlation and association analyses

A gene function prediction website (GeneMANIA: http://genemania.org/, accessed Oct. 15, 2018) was used to analyze interactions among GSTM family members [16]. As well as The Search Tool for the Retrieval of Interacting Genes/Proteins (STRING) database (http://string.embl.de/; accessed Oct.15, 2018), and those with a required interaction score > 0.15 were considered statistically significant [17].

### Correlation matrix of GSTM genes in COAD

Pearson’s correlation coefficient (r) is used to evaluate the association between GSTM genes in COAD. A correlation coefficient r ≥ 0.4 or r ≤ − 0.4 was considered to reflect a high correlation. *P* value less than 0.01 was considered statistically significant.

### Clinical significance of GSTM genes in COAD

For each GSTM gene, patients were evenly divided into high- and low-expression groups by median expression. The Kaplan-Meier estimator was applied to identify correlations between the genes and patient overall survival (OS). Multivariate Cox proportional hazards regression model was adjusted for tumor stage.

### Nomogram for predicting the prognosis of COAD

A nomogram was generated to predict the prognostic outcome and risk rank. All GSTM genes and clinical information were included in the nomogram model. Points were positively correlated with risk, and the points corresponding to each parameter were assessed. Prognosis was predicted at 1, 5 and 10 years [18].

### Joint-effects survival analysis

In order to further improve the prognostic ability of GSTM genes in COAD OS, we further analyzed the combined effects of prognostic GSTM genes combinations.

### Gene set enrichment analysis (GSEA)

Biological function differences between GSTM gene phenotypes with different expression levels were explored using GSEA v.3.0 with reference to gene sets of c2 (c2.all.v6.1.symbols.gmt) and c5 (c5.all.v6.1.symbols.gmt) gene set, respectively [19]. Enrichment results meeting *P* < 0.05 and a false discovery rate (FDR) < 0.25 were considered to be significantly different between the two groups.

### Statistical analysis

Hazard ratios (HRs) and 95% confidence intervals (CIs) were used to assess the risk ratios of survival differences between groups. *P* < 0.05 was considered to be significantly different between groups. SPSS v.25.0 (IBM, Chicago, IL, USA) and GraphPad v.7.0 (La Jolla, CA, USA) are used for statistical analysis and figures drawing respectively. Figures plotting was performed by R v.3.5.1 and Cytoscape v3.6.1 [20].

## Results

### Data analysis

**Fig. 1:**
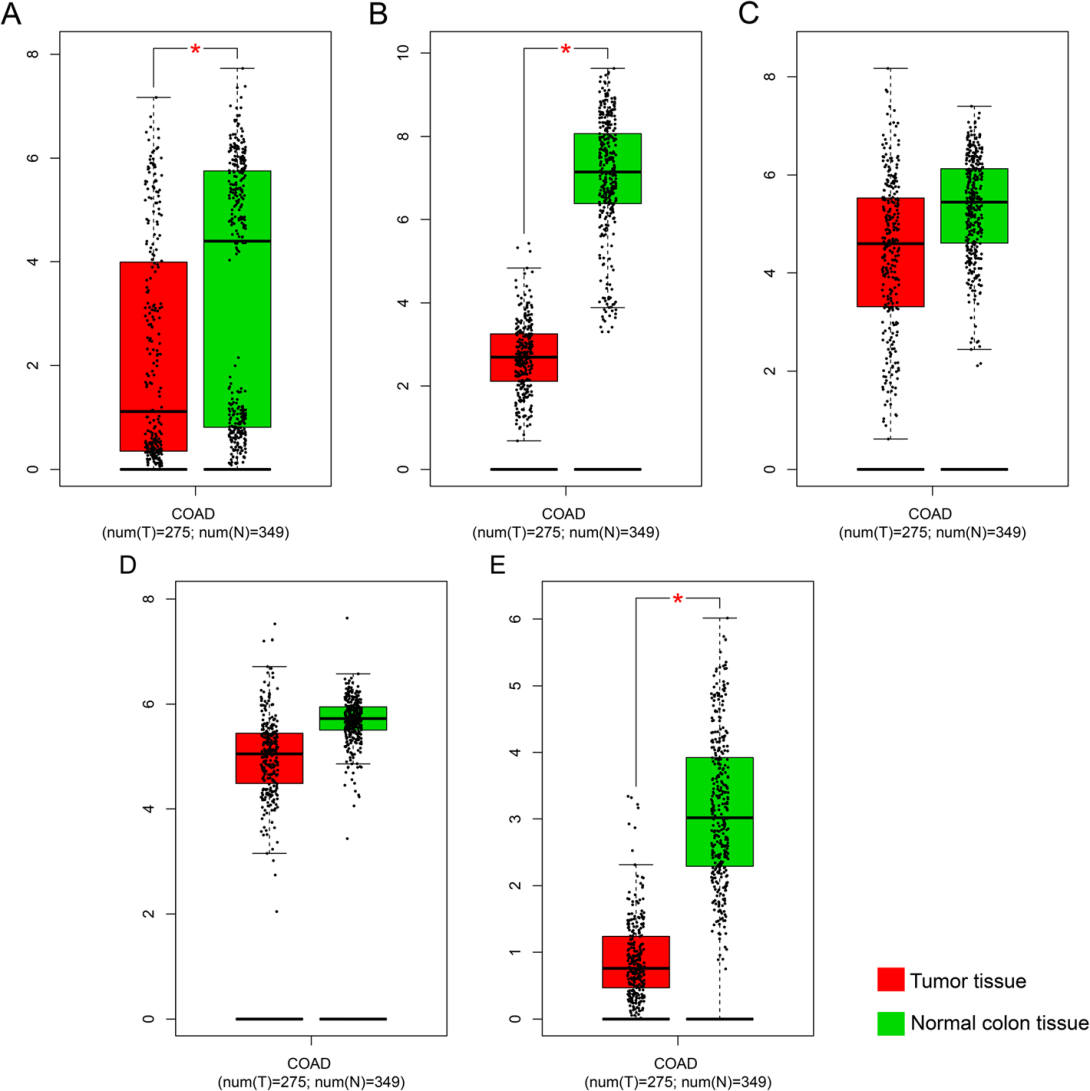
Boxplots for GSTM gene family expression in colon tumor and normal tissues by Gene Expression Profiling Interactive Analysis (GEPIA). (**a**) GSTM1; (**b**) GSTM2; (**c**) GSTM3; (**d**) GSTM4; (**e**) GSTM5

After selection, 438 cases were included in the analysis (Table S1). Tumor stage was the only factor associated with favorable prognosis; earlier tumor stages were associated with a more favorable prognosis. Expression profile of GSTM genes are summarized in Fig. 1. *GSTM1*, *GSTM2*, and *GSTM3* were expressed at significantly higher levels in normal tissues than in colon tumor tissues (Fig. 1A, B, and E).

### Functional enrichment analysis of *GSTM* genes

**Fig. 2:**
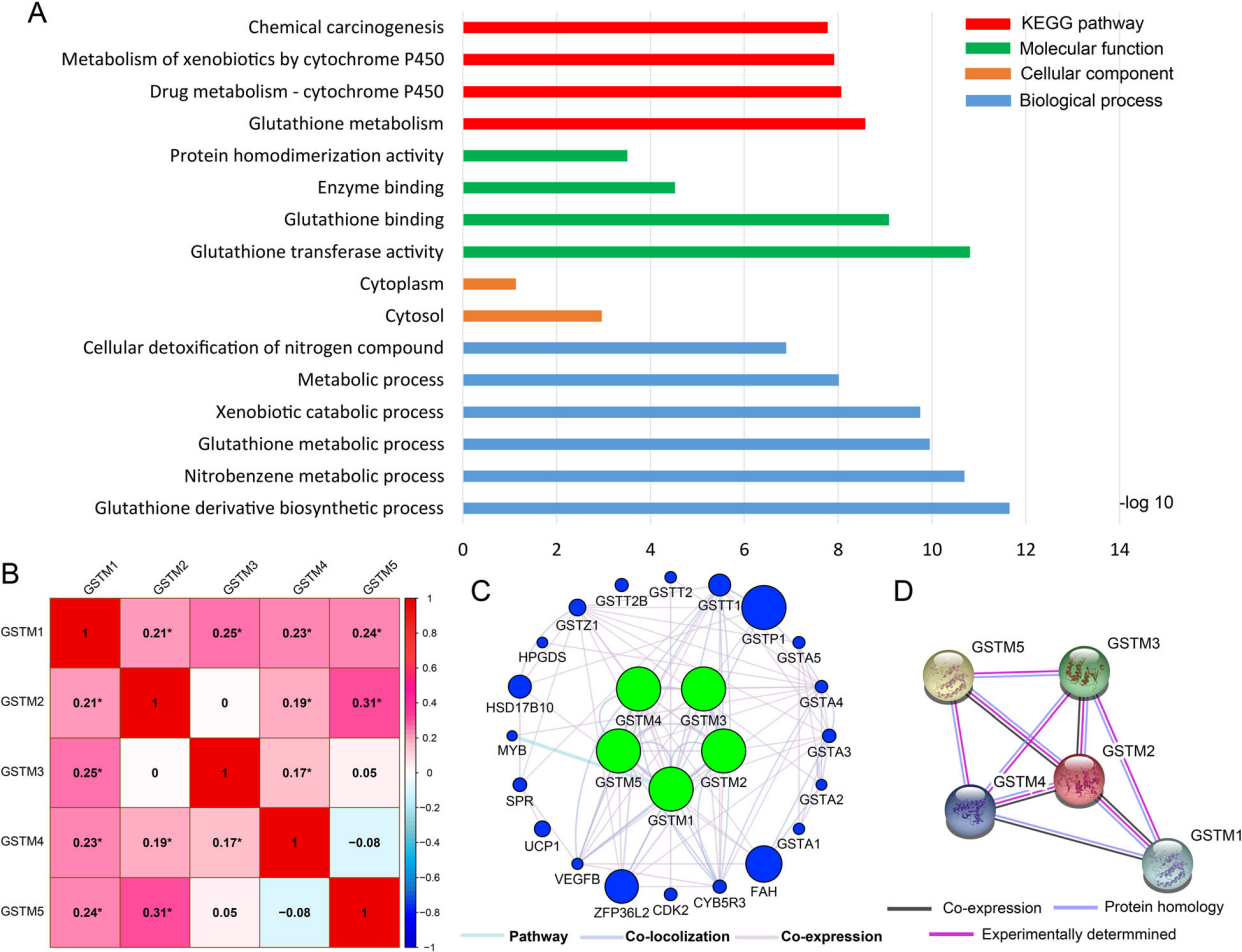
(**a**) GO enrichment and KEGG pathway analysis by DAVID; (**b**) Pearson’s correlation coefficients between GSTM gene expression levels. *P < 0.001; (**c**) Gene-gene interaction network for GSTM gene family by GeneMANIA. (**d**) Protein-protein interaction network for GSTM gene family by STRING

Functional enrichment of *GSTM* genes were evaluated for gene ontology (GO) functional and Kyoto Encyclopedia of Genes and Genomes (KEGG) pathway analyses (Fig. 2A). The results of BiNGO enrichment analysis are shown in Fig. S1. There were no results for CC in this enrichment. The GSTM family was involved in metabolic processes and glutathione-related processes including metabolism, transfer, and binding. Correlation analysis between the *GSTM* family is shown in Fig. 2B. There were no significant associations between *GSTM2* and *GSTM3*, *GSTM3* and *GSTM5*, or *GSTM4* and *GSTM5*. The other genes were markedly related to each other (*P* < 0.01). The correlation of *GSTM1* and *GSTM2* with matrix gene expression is shown in Figs. S2 and S3 (all *P* < 0.05 and correlation coefficient > 0.4). Co-expression analysis of the GSTM family at the mRNA level by GeneMANIA is shown in Fig. 2C. The PPI network determined by STRING is shown in Fig. 2D.

### Survival analysis

**Fig. 3:**
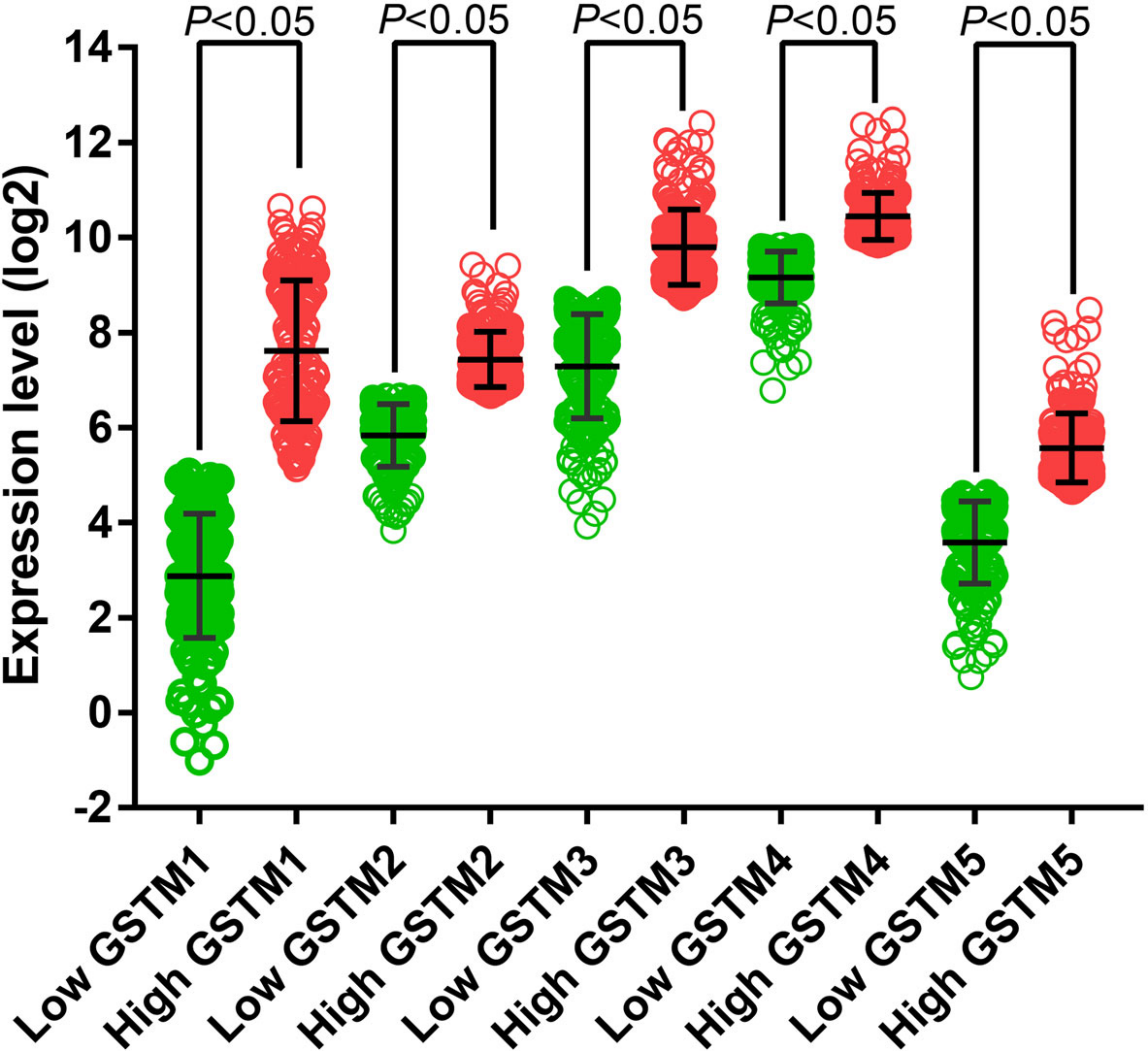
Scatter plots for GSTM1, GSTM2, GSTM3, GSTM4, and GSTM5 gene expression levels in The Cancer Genome Atlas

**Fig. 4:**
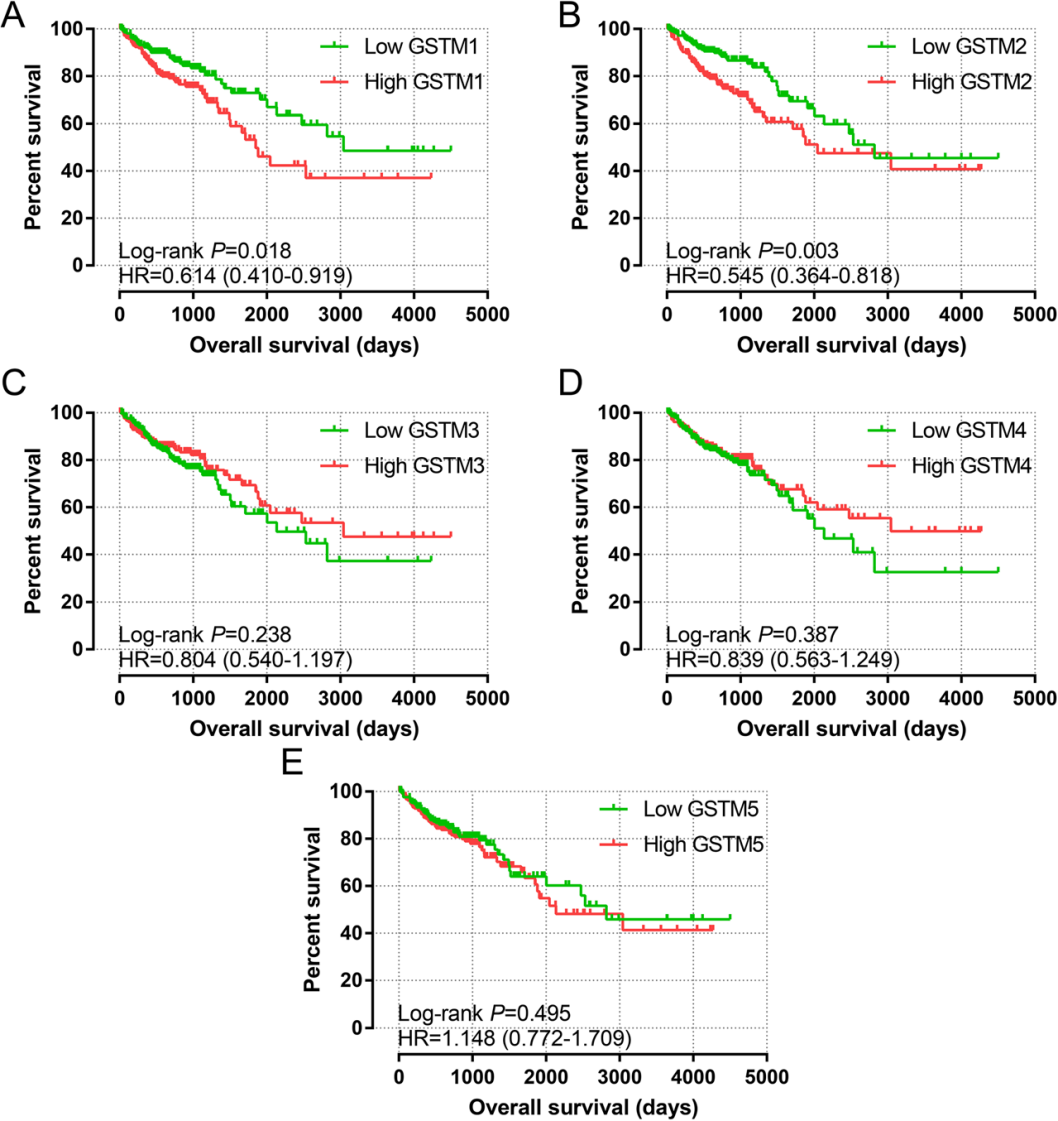
Prognostic value of GSTM expression for OS. (**a**–**e**) Kaplan-Meier survival curves for COAD patients according to GSTM1 (**a**), GSTM2 (**b**), GSTM3 (**c**), GSTM4 (**d**), and GSTM5 (**e**) expression (*n* = 438)

Vertical scatter plots for the expression of the GSTM genes are shown in Fig. 3. Differences between high- and low-expression groups were markedly difference (all *P* < 0.05). Survivorship curves of GSTM genes are summarized in Fig. 4A–E. Only low expression of *GSTM1* and *GSTM2* was markedly related to favorable prognosis (*P* = 0.018, HR = 0.614, 95% CI = 0.410–0.919, Fig. 4A; *P* = 0.003, HR = 0.545, 95% CI = 0.364–0.818, Fig. 4B, respectively). The multivariate Cox proportional hazard regression model only included tumor stage. The results are summarized in Table 1. The results of univariate survival analysis were consistent with those of multivariate survival analysis: low expression of *GSTM1* and *GSTM2* was markedly related to favorable OS (adjusted *P* = 0.006, adjusted HR = 0.559, 95% CI = 0.367–0.849; adjusted *P* = 0.002, adjusted HR = 0.519, 95% CI = 0.342–0.790, respectively).

**Table 1:** Prognostic survival analysis results

Gene	Patients (*n* = 438)	No. of events (%)	MST (days)	Crude HR (95% CI)	Crude P	Adjusted HR* (95% CI)	Adjusted P*
***GSTM1***							
High	219	57 (26.0%)	1849	Ref.	**0.018**	Ref.	**0.006**
Low	219	41 (18.7%)	3042	0.614 (0.410–0.919)		0.559 (0.367–0.849)	
***GSTM2***							
High	219	59 (26.9%)	2047	Ref.	**0.003**	Ref.	**0.002**
Low	219	39 (17.8%)	2821	0.545 (0.364–0.818)		0.519 (0.342–0.790)	
***GSTM3***							
High	219	45 (20.5%)	3042	Ref.	0.804	Ref.	0.469
Low	219	53 (24.3%)	2134	0.804 (0.540–1.197)		0.860 (0.571–1.295)	
***GSTM4***							
High	219	46 (21.0%)	3042	Ref.	0.387	Ref.	0.729
Low	219	52 (23.7%)	2134	0.839 (0.563–1.249)		0.930 (0.618–1.400)	
***GSTM5***							
High	219	53 (24.2%)	2134	Ref.	0.495	Ref.	0.903
Low	219	45 (20.5%)	2821	1.148 (0.772–1.709)		0.975 (0.647–1.468)	

Notes: *, adjustment for tumor stage

Abbreviations: GSTM, Glutathione S-transferase Mu; MST, median survival time; HR, hazard ratio; CI, confidence interval

### Nomogram for predicting outcome

**Fig. 5:**
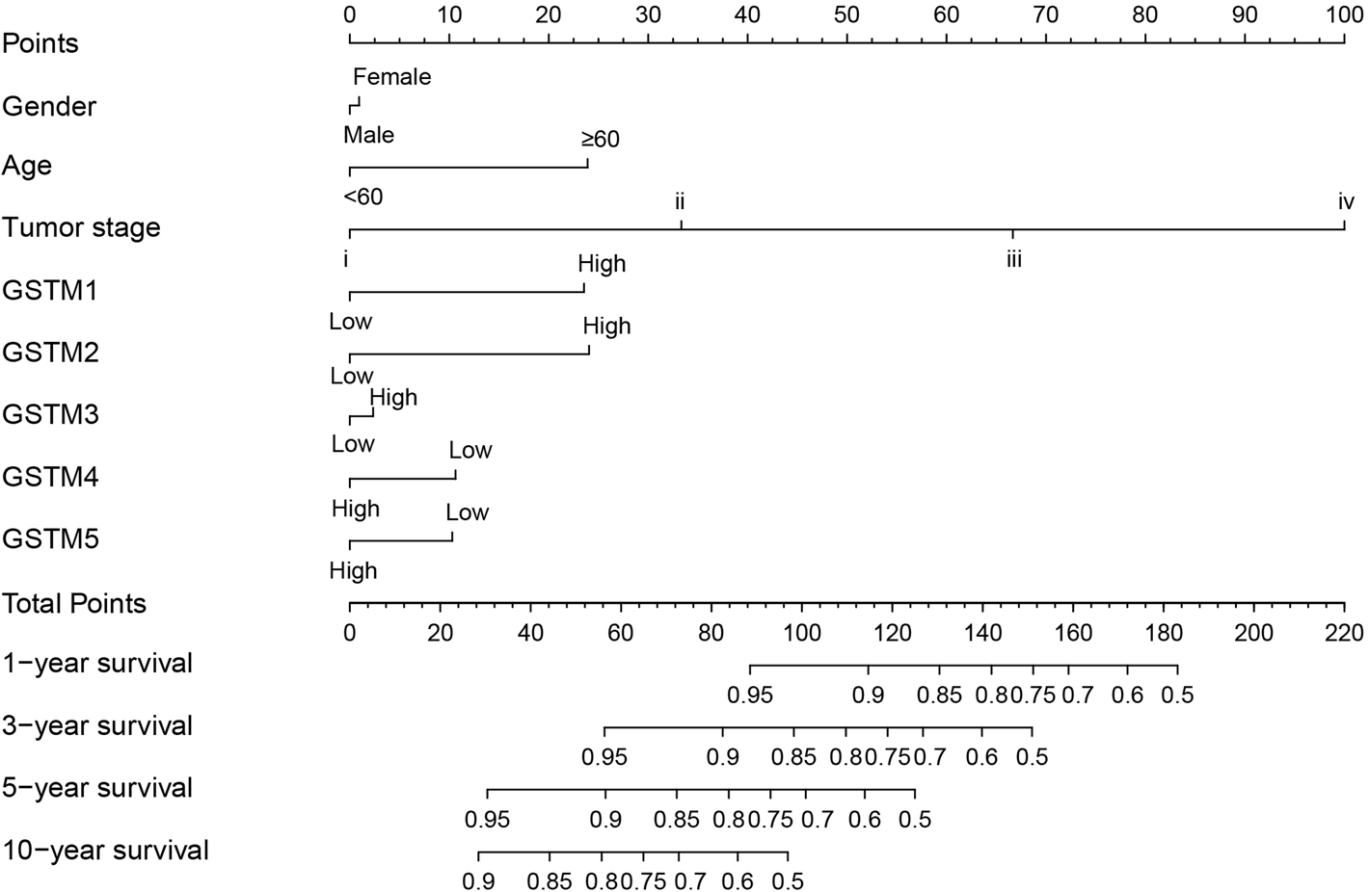
Nomogram for predicting the relationship between the risk score and clinical information

The nomogram for predicting the prognostic value is shown in Fig. 5. Regarding clinical data, tumor stage provided the highest contribution risk score, and high expression of *GSTM1* and *GSTM2* showed higher contribution risk scores for COAD patients.

### Joint-effects survival analysis

**Fig. 6:**
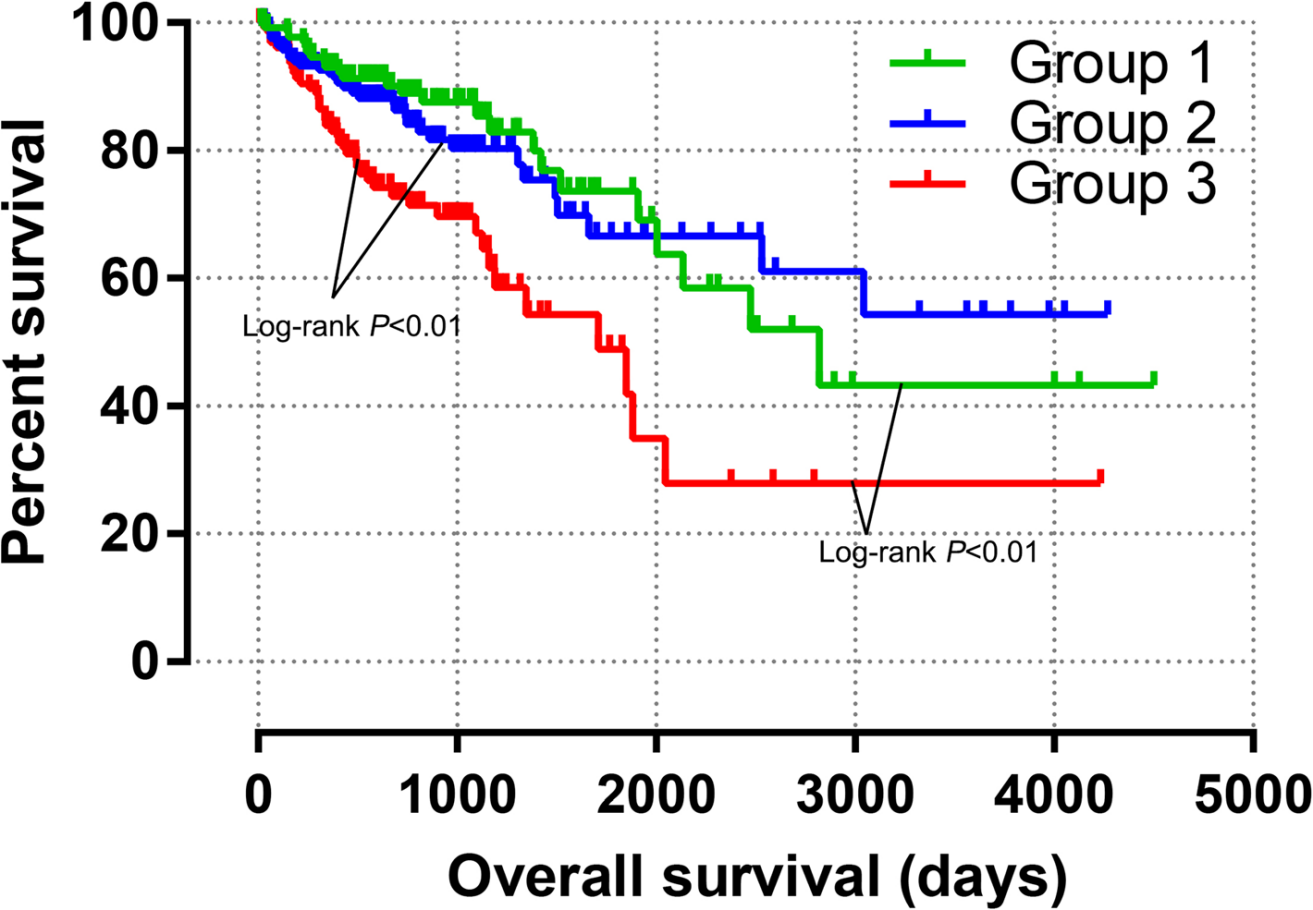
Joint-effects analysis of *GSTM1* and *GSTM2* expression level combinations

The grouping situation is summarized in Table 2. Group 1 showed the expression level combination related to favorable OS (low expression of *GSTM1* and *GSTM2*). Group 3 included the combination associated with worse OS (high expression of *GSTM1* and *GSTM2*). Compared with Group 3, Group 1 and Group 2 was related to favorable prognosis (all *P* < 0.05, Fig. 6, Table 3).

**Table 2:** The grouping information of joint-effects analysis

Group	Combinations
1	Low GSTM1 + Low GSTM2
2	High GSTM1 + Low GSTM2 Low GSTM1 + High GSTM2
3	High GSTM1 + High GSTM2

Abbreviations: GSTM, Glutathione S-transferase Mu

**Table 3:** Joint-effects analysis of the prognostic value of combinations of *GSTM1* and *GSTM2*

Group	Patients (n = 438)	No. of events (%)	MST (days)	Crude *P*	Crude HR (95% CI)	Adjusted P*	Adjusted HR* (95% CI)
**1**	134	24 (17.9%)	2821	**0.001**	0.421 (0.254–0.697)	**0.001**	0.416 (0.251–0.689)
**2**	170	32 (12.4%)	N/A	**0.001**	0.467 (0.293–0.744)	**0.001**	0.469 (0.294–0.689)
**3**	134	42 (31.3%)	1711	**< 0.001**	Ref.	**< 0.001**	Ref.

Notes: *, adjustment for tumor stage

Abbreviations: GSTM, Glutathione S-transferase Mu; MST, median survival time; HR, hazard ratio; CI, confidence interval

### GSEA

**Fig. 7:**
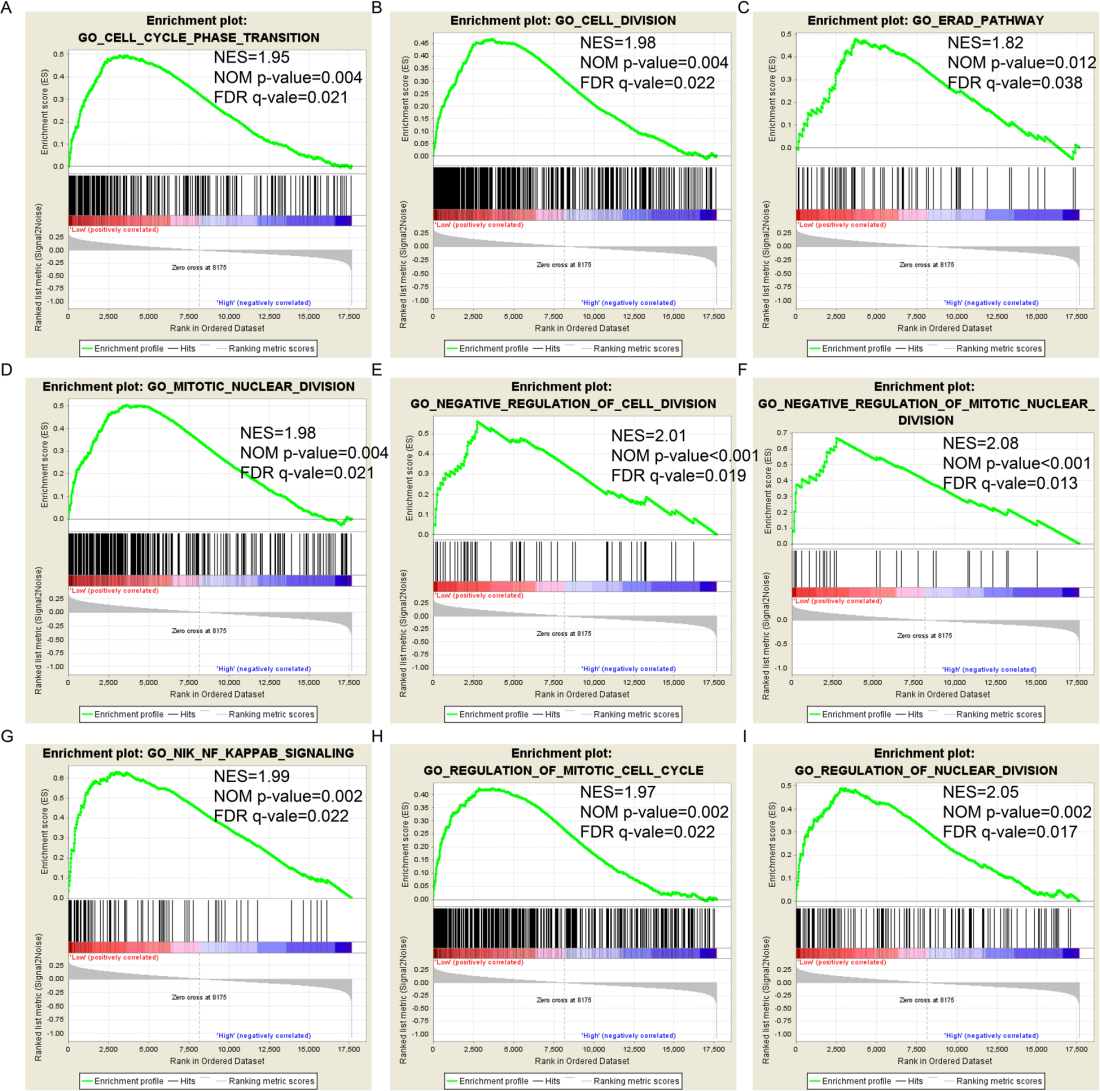
GO enrichment results by GSEA for *GSTM2*. (**a**) Cell cycle phase transition; (**b**) Cell division; (**c**) Erad pathway; (**d**) Mitotic nuclear division; (**e**) Negative regulation of cell division; (**f**) Negative regulation of mitotic nuclear division; (**g**) NIK/NF kappa B signaling; (**h**) Regulation of mitotic cell cycle; (**i**) Regulation of nuclear division

**Fig. 8:**
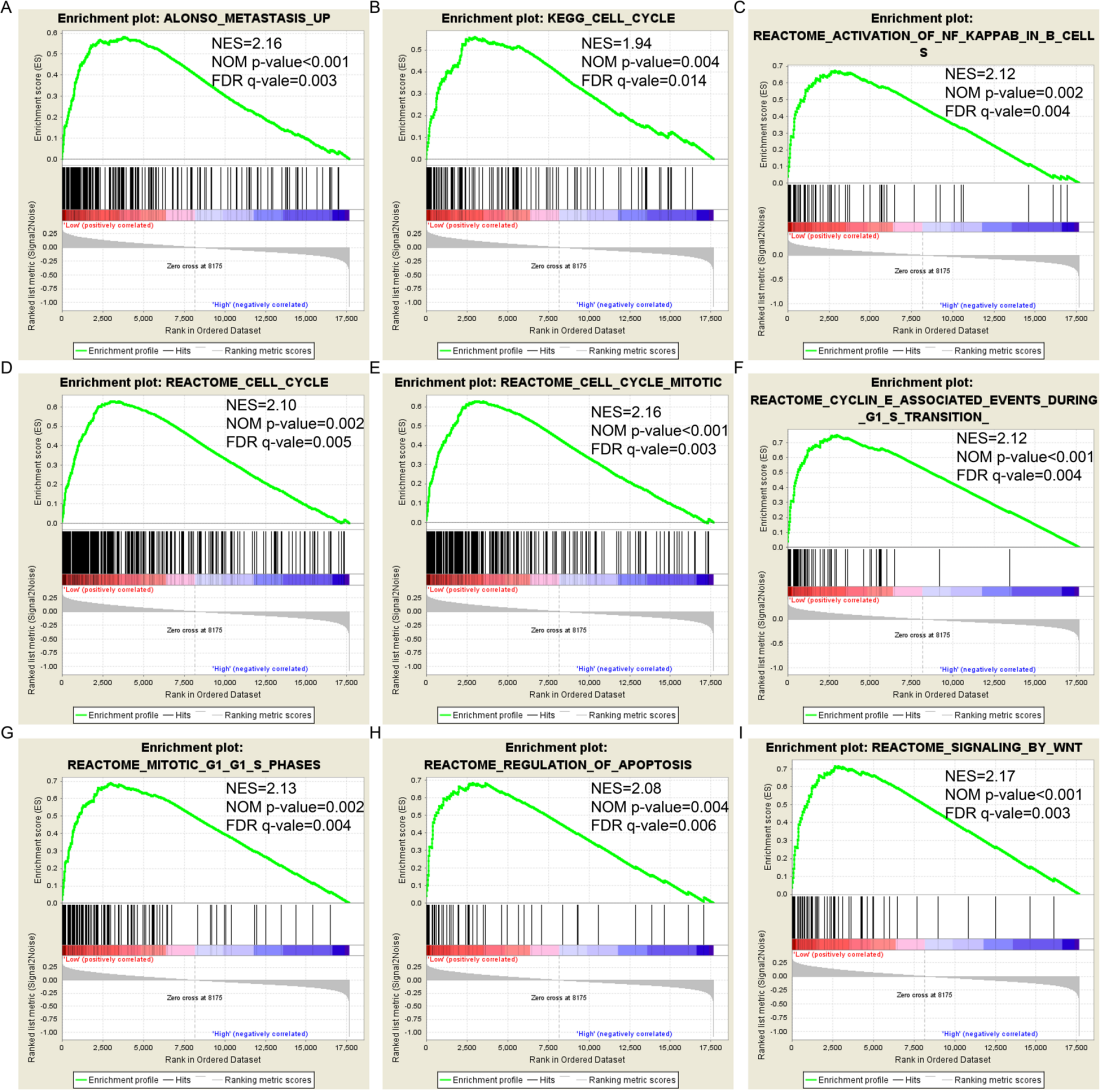
KEGG enrichment result by GSEA for *GSTM2.* (**a**) Metastasis up; (**b**) Cell cycle; (**c**) Activation of NF kappa B in B cells; (**d**) Cell cycle; (**e**) Cell cycle mitotic; (**f**) Cyclin E associated events during G1 S transition; (**g**) Mitotic G1/G1/S phases; (**h**) Regulation of apoptosis; (**i**) Signaling by Wnt

GSEA was performed to predict the effect of *GSTM1* and *GSTM2* low expression on prognosis. There were no statistically significant enrichment results for *GSTM1* in both GO and KEGG analyses. The GO and KEGG enrichment results are shown in Fig. 7A–I and Fig. 8A–I, respectively. For GO enrichment, low expression of *GSTM2* was associated with cell division (Fig. 7B, D, E, F, and I), cell cycle (Fig. 7A and H), the NIF/NF-κB signaling pathway (Fig. 7G), and the ERAD pathway (Fig. 7C). For KEGG enrichment, low expression of *GSTM2* was associated with cell metastasis (Fig. 8A), cell cycle (Fig. 8B, D, E, F, and G), activation of NF-κB (Fig. 8C), cell apoptosis (Fig. 8H), and the WNT signaling pathway (Fig. 8I). The results are shown in Tables S2 and S3.

## Discussion

In the current study, we investigated the expression level of the *GSTM* gene family in COAD, and performed a survival analysis including clinical data and *GSTM* gene expression. A nomogram model was used to predict the outcomes of COAD patients, and joint-effects survival analysis show that the combination of *GSTM1* and *GSTM2* low expression was a sensitive predictor of favorable prognosis. GSEA and serval enrichment analysis were performed to explain the effects of low expression level of *GSTM1* and *GSTM2* on prognosis.

*GSTMs* belong to the sub-family of soluble *GSTs* and include five members, *GSTM1*, *GSTM2*, *GSTM3*, *GSTM4*, and *GSTM5* [8–11]. GSTs play important roles and are associated with glutathione (GSH) in the detoxification process [5–7]. Several *GSTs* are involved in the MAPK pathway, which controls cell proliferation, cell differentiation, and cell death, including the subfamilies *GSTA*, *GSTP*, and *GSTM* [21].

*GSTM1* is polymorphic in humans, and 40–60% of the population have a homozygous deletion of this gene [22]. Therefore, most studies of GSTM1 are performed using GSTM1-wt (wild-type genotype) and GSTM1-null (null genotype). Combining *GSTM1* and p53 variants can divide colorectal cancer patients into several subgroups with significantly different prognosis, *GSTM1+* polymorphism was associated with favorable OS in patients with colorectal cancer [23]. In ovarian cancer, GSTM1-null patients have a significant better survival than GSTM-wt patients, [24, 25] which could be attributed to the effect of GSTM1 on the expression of the p53 gene [25]. A previous study showed that *GSTM1* induces tumor resistance by hydrolyzing tumor chemotherapy drugs or activating anti-apoptotic pathways, [26] and it was shown to be a negative regulator of apoptosis-related signaling cascades [22]. GSTM1 functions as a tumor suppressor gene in hepatocellular carcinoma; however, the prognostic value was not reported [27]. *GSTM1* is also a risk factor of relapse in childhood acute lymphoblastic leukemia and hepatocellular carcinoma [24, 28, 29]. *GSTM1* may also affect OS in breast cancer [30]. In gastric cancer, *GSTM1*-wt patients show better tumor-related and disease-free survival [31]. However, in the study of Acevedo et al., there was no significant correlation between GSTM1 polymorphisms and prognosis of prostate cancer [32].

GSTM2, a striated muscle-specific isozyme, [33] is highly expressed in mouse liver cancer, and involved in the Wnt/beta-catenin pathway [34]. In prostate cancer, GSTM2 is a potential tumor suppressor [35]. *GSTM2* is among phase I or II metabolism-related genes, which were from phase II-conjugation [36]. These results are consistent with our GO enrichment results. *GSTM2* is expressed at low levels in lung cancer [37]. There are no further reports about the relationship between the *GSTM2* and cancer prognosis.

In the present study, low expression of *GSTM1* and *GSTM2* and their combination were associated with favorable OS in COAD patients. GSTM1 and GSTM2 are involved in cell cycle and detoxification, and tumor-inhibiting cytokines may be degraded by the expression of *GSTM1* and *GSTM2* by speculating the results of enrichment analysis. However, GSTs can also degrade carcinogenic compounds. Therefore, further studies of the combination, connections, interactions, and synergy among GSTs family members are needed.

Regarding *GSTM3*, mutation of this gene may increase the risk of bladder cancer [38]. Polymorphisms of the GSTM4 gene are associated with increased risk of lung cancer [39] and could be used as a biomarker for the prediction of cisplatin response [40]. There are no reports on the relationship between cancer and the expression level of GSTM5.

The Wnt signaling pathway is critical for the development of colon cancer and patient outcome [41, 42]. GSTM2 is related to the Wnt signaling pathway, [34] which is consistent with the present enrichment results. This could explain the results showing that low expression of *GSTM2* was related to favorable prognosis. In addition, predictive function of low GSTM2 and GSTM1 were involved in the cell cycle, which is associated with the occurrence of cancers and outcome.

Previous studies of GSTM genes focused on the GSTM-null and GSTM-wt genotypes and their association with the risk and susceptibility to cancers. We found that low expression of *GSTM1* and *GSTM2* and their combination were correlated with favorable OS, and the nomogram showed that 1-, 5-, 10-year survival rates were affected by low expression levels of *GSTM1* and *GSTM2*.

Our study had several disadvantages. First, further studies with a larger sample size are needed due to the small sample size of our study, and additional verification cohorts still need to verify our results. Second, due to the limited clinical data provided by TCGA, many factors affecting the prognosis of COAD cannot be included in the Cox model for correction. Third, because of the polymorphisms of *GSTM* genes, the genotype should also be included. Despite the above disadvantages, the present study is the first to report the relationship between the prognosis of COAD and *GSTM* gene family. These results suggest that low *GSTM1* and *GSTM2* expression was related to favorable prognosis in COAD. These two genes may be used as prognostic biomarkers for predicting the outcomes of COAD patients.

## Conclusion

Our study showed that the *GSTM1* and *GSTM2* expression was down-regulated in COAD, and low expression was markedly related to favorable prognosis. GSEA was performed to predict the function and mechanism. The results of GSEA indicated that the cell metabolism and detoxification functions of *GSTM1* and *GSTM2* may affect the prognosis of COAD patients. A nomogram including clinical information and gene expression levels was generated to predict the risk score for each factor. GSTM1 and GSTM2 seem interesting candidates for further studies aimed to validate their use as biomarkers of prognosis in COAD. Therefore, our findings can be used as preliminary support data for GSTM1 and GSTM2 as potential prognostic biomarkers for COAD. However, further studies are needed to confirm the present results.

## Supplementary information

**Additional file 1: Figure S1.** GO functional enrichment analysis by BiNGO of GSTM family.

**Additional file 2: Figure S2.** Gene interaction network for the GSTM1 gene and potentially related COAD gene cohort in TCGA.

**Additional file 3: Figure S3.** Gene interaction network for the GSTM2 gene and potentially related COAD gene cohort in TCGA.

**Additional file 4: Table S1.** Clinical information.

**Additional file 5: Table S2.** KEGG enrichment result by GSEA for *GSTM2* (c2.all.v6.2.symbols.gmt).

**Additional file 6: Table S3.** GO enrichment results by GSEA for *GSTM2* (c5.all.v6.2.symbols.gmt).

## Data Availability

The raw datasets used during the present study can be downloaded from The Cancer Genome Atlas (https://portal.gdc.cancer.gov/projects/TCGA-COAD). The COAD RNA-seq dataset are open assess to everyone and can be downloaded directly from The Cancer Genome Atlas website without any login account.

## Abbreviations

COAD: Colon adenocarcinoma
*GSTM*: Glutathione S-transferase Mu
GSEA: Gene set enrichment analysis
TCGA: The Cancer Genome Atlas
GEPIA: Gene Expression Profiling Interactive Analysis
DAVID: Database for Annotation, Visualization, and Integrated Discovery
STRING: Search Tool for the Retrieval of Interacting Genes/Proteins
PPI: Protein–protein interaction
OS: Overall survival
MSigDB: Molecular Signatures Database
FDR: False discovery rate
