# Supplementary figures and images for: Clinical significance and biological mechanisms of glutathione S-transferase mu gene family in colon adenocarcinoma
Source: BMC Med Genet. 2020 Jun 15;21:130. doi: 10.1186/s12881-020-01066-2 (PMC7296959; doi:10.1186/s12881-020-01066-2)

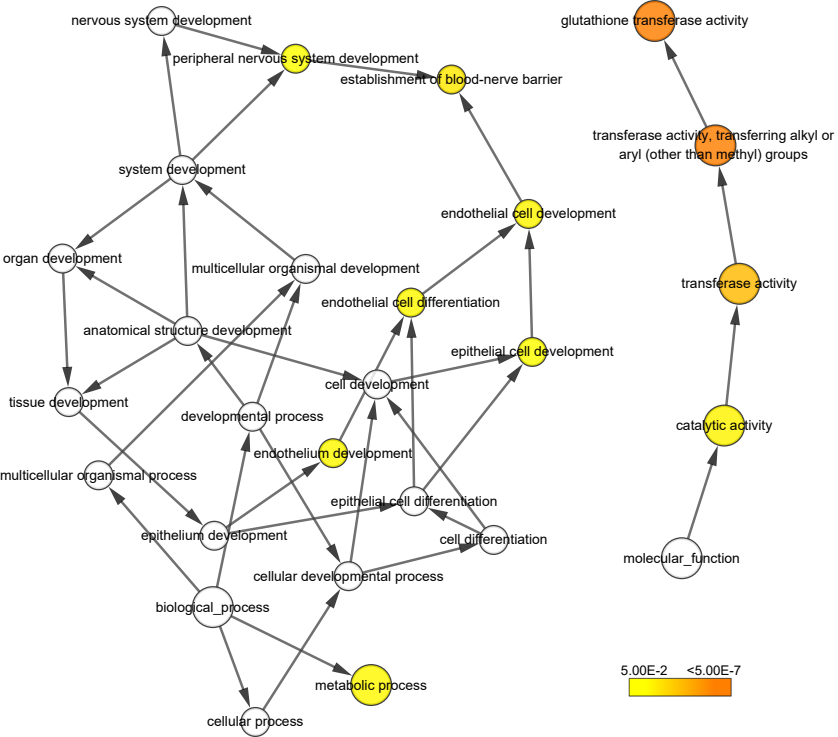

Supplement: Supplementary file 1 — Additional file 1: Figure S1. GO functional enrichment analysis by BiNGO of GSTM family. [file 12881_2020_1066_MOESM1_ESM.pdf]

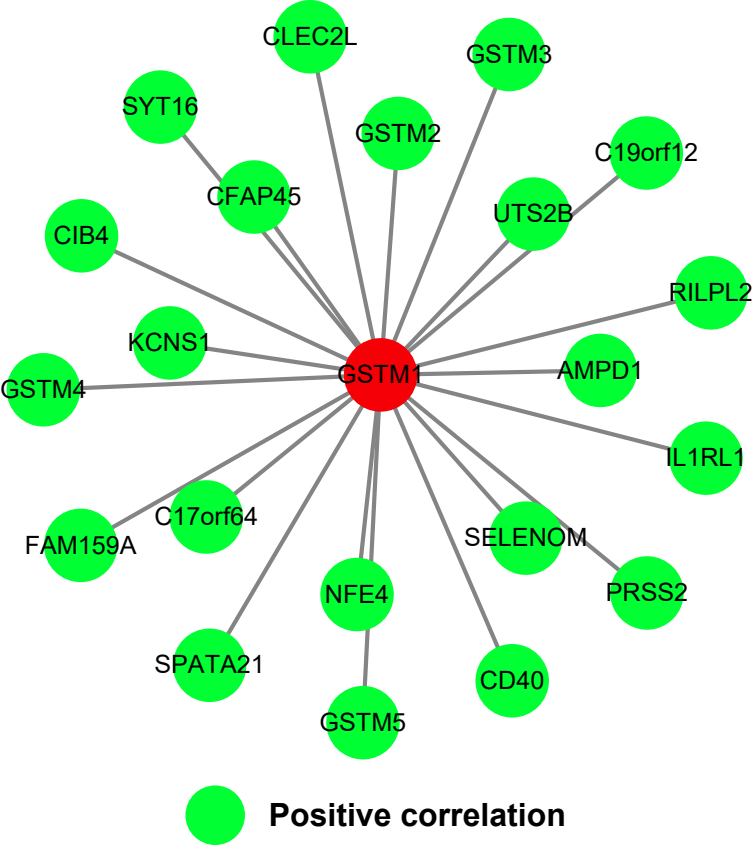

Supplement: Supplementary file 2 — Additional file 2: Figure S2. Gene interaction network for the GSTM1 gene and potentially related COAD gene cohort in TCGA. [file 12881_2020_1066_MOESM2_ESM.pdf]

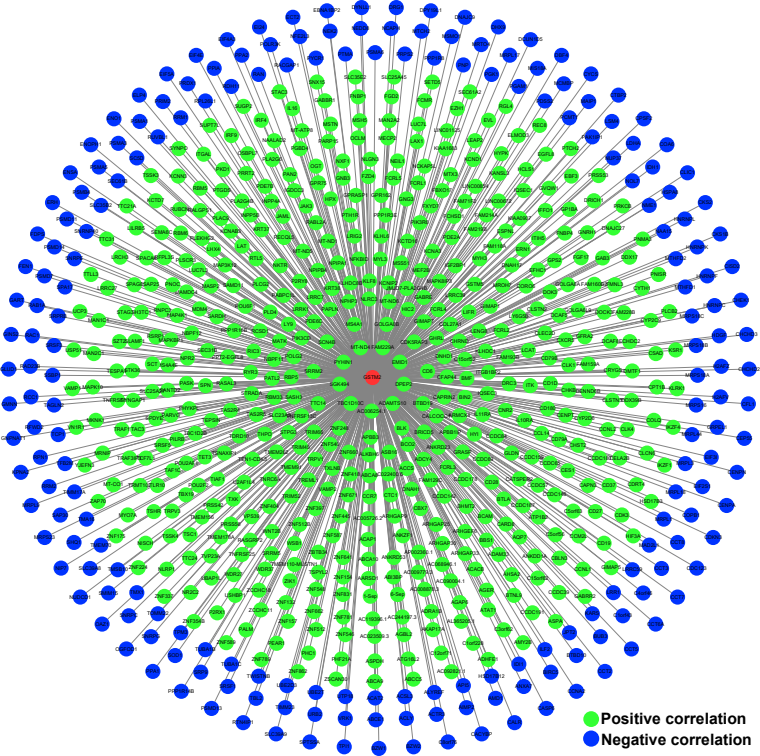

Supplement: Supplementary file 3 — Additional file 3: Figure S3. Gene interaction network for the GSTM2 gene and potentially related COAD gene cohort in TCGA. [file 12881_2020_1066_MOESM3_ESM.pdf]
