# Supplementary material for: Clinical significance and biological mechanisms of glutathione S-transferase mu gene family in colon adenocarcinoma
Source: BMC Med Genet. 2020 Jun 15;21:130. doi: 10.1186/s12881-020-01066-2 (PMC7296959; doi:10.1186/s12881-020-01066-2)
Supplement: Supplementary file 4 — Additional file 4: Table S1. Clinical information. [file 12881_2020_1066_MOESM4_ESM.pdf]

Table S1 Clinical data for 438 patients

Variables	Patients (n=438)	No. of events (%)	MST (days)	HR (95% CI)	Log-rank *P*
Gender					
Male	234	54 (23.1%)	2475	Ref.	0.545
Female	204	44 (21.6%)	N/A	1.131 (0.759-1.686)	
Age (years)					
≥60	314	74 (23.6%)	2475	Ref.	0.387
<60	122	23 (18.9%)	N/A	0.813 (0.509-1.300)	
missing	2				
Tumor stage					
i	73	4 (5.4%)	N/A	Ref.	**<0.001**
ii	167	27 (16.2%)	2821	0.089 (0.031-0.251)	
iii	126	31 (24.6%)	N/A	0.198 (0.118-0.335)	
iv	61	31(50.8%)	858	0.360 (0.218-0.596)	
missing	11				

Abbreviations: MST, median survival time; HR, hazard ratio; CI, confidence interval; N/A, Not applicable.
