# Supplementary material for: Clinical significance and biological mechanisms of glutathione S-transferase mu gene family in colon adenocarcinoma
Source: BMC Med Genet. 2020 Jun 15;21:130. doi: 10.1186/s12881-020-01066-2 (PMC7296959; doi:10.1186/s12881-020-01066-2)
Supplement: Supplementary file 5 — Additional file 5: Table S2. KEGG enrichment result by GSEA for GSTM2 (c2.all.v6.2.symbols.gmt). [file 12881_2020_1066_MOESM5_ESM.pdf]

Table S2 KEGG enrichment result by GSEA for *GSTM2* (c2.all.v6.2.symbols.gmt)

NAME	SIZE	ES	NES	NOM p-val	FDR q-val
PENG_GLUTAMINE_DEPRIVATION_DN	324	0.641568	2.314537	<0.0001	8.47E-04
RHEIN_ALL_GLUCOCORTICOID_THERAPY_DN	348	0.661351	2.315424	<0.0001	0.001059
TARTE_PLASMA_CELL_VS_PLASMABLAST_DN	300	0.654672	2.270752	<0.0001	0.001308
WONG_PROTEASOME_GENE_MODULE	49	0.731322	2.272478	<0.0001	0.001373
BORCZUK_MALIGNANT_MESOTHELIOMA_UP	291	0.647288	2.320954	<0.0001	0.001412
PENG_LEUCINE_DEPRIVATION_DN	182	0.661383	2.258491	<0.0001	0.001475
PENG_RAPAMYCIN_RESPONSE_DN	229	0.662135	2.281554	<0.0001	0.001602
CHIANG_LIVER_CANCER_SUBCLASS_UNANNOTATED_DN	182	0.739816	2.247181	<0.0001	0.001633
BASSO_B_LYMPHOCYTE_NETWORK	134	0.568031	2.23562	<0.0001	0.001692
WEST_ADRENOCORTICAL_TUMOR_UP	282	0.624028	2.373334	<0.0001	0.001732
BOYAULT_LIVER_CANCER_SUBCLASS_G3_UP	181	0.698385	2.236032	<0.0001	0.001846
CHANG_CORE_SERUM_RESPONSE_UP	198	0.645945	2.225785	<0.0001	0.002052
LEE_LIVER_CANCER_SURVIVAL_DN	165	0.700705	2.323513	<0.0001	0.002118
REACTOME_CELL_CYCLE	384	0.629451	2.209381	<0.0001	0.002548
KARLSSON_TGFB1_TARGETS_UP	118	0.6051	2.1958	<0.000	0.0026

		24	65	1	12
**YAO_TEMPORAL_RESPONSE_TO_PROGESTERONE_CLUSTER_10**	64	0.6936	2.2155	<0.000	0.0026
		99	64	1	2
**OUELLET_OVARIAN_CANCER_INVASIVE_VS_LMP_UP**	117	0.6439	2.1650	<0.000	0.0027
		52	03	1	09
**RHODES_CANCER_META_SIGNATURE**	63	0.7202	2.1963	<0.000	0.0027
		17	39	1	66
**STARK_PREFRONTAL_CORTEX_22Q11_DELETION_DN**	470	0.6043	2.1734	<0.000	0.0027
		5	99	1	69
**NAKAMURA_TUMOR_ZONE_PERIPHERAL_VS_CENTRAL_UP**	267	0.5234	2.1681	<0.000	0.0028
		52	83	1	16
**BARIS_THYROID_CANCER_UP**	23	0.7136	2.1650	<0.000	0.0028
		45	21	1	18
**VECCHI_GASTRIC_CANCER_EARLY_UP**	384	0.5984	2.1806	<0.000	0.0028
		8	07	1	34
**HORIUCHI_WTAP_TARGETS_DN**	293	0.6339	2.1821	<0.000	0.0028
		74	56	1	77
**REACTOME_SIGNALING_BY_WNT**	62	0.7142	2.1655	<0.000	0.0029
		69	42	1	03
**GRADE_COLON_AND_RECTAL_CANCER_UP**	272	0.6300	2.1971	<0.000	0.0029
		04	55	1	39
**REACTOME_REGULATION_OF_MITOTIC_CELL_CYCLE**	77	0.7478	2.1683	<0.000	0.0029
		45	98	1	44
**REACTOME_CELL_CYCLE_MITOTIC**	299	0.6280	2.1606	<0.000	0.0029
		1	21	1	58
**LY_AGING_OLD_DN**	55	0.7189	2.1507	<0.000	0.0029
		69	7	1	68
**REACTOME_HIV_INFECTION**	184	0.5899	2.1584	<0.000	0.0029
		09	5	1	82
**CUI_GLUCOSE_DEPRIVATION**	59	0.6126	2.1466	<0.000	0.0029
		77	06	1	93
**LINDGREN_BLADDER_CANCER_CLUSTER_3_UP**	304	0.6118	2.1477	<0.000	0.0030

		67	32	1	32
**MALONEY_RESPONSE_TO_17AAG_DN**	76	0.7130	2.1508	<0.000	0.0030
		8	72	1	58
**ALONSO_METASTASIS_UP**	186	0.5824	2.1575	<0.000	0.0030
		41	69	1	69
**CAFFAREL_RESPONSE_TO_THC_DN**	29	0.7659	2.1511	<0.000	0.0031
		34		1	54
**REACTOME_REGULATION_OF_MRNA_STABILITY_BY_PROTEINS_THAT_BIND_AU_RICH_ELEMENTS**	81	0.6583	2.1515	0.0020	0.0032
		66	19	41	55
**BENPORATH_PROLIFERATION**	136	0.7353	2.1531	<0.000	0.0032
		52	91	1	64
**REACTOME_APC_C_CDH1_MEDIATED_DEGRADATION_OF_CDC20_AND_OTHER_APC_C_CDH1_TARGETED_PROTEINS_IN_LATE_MI**	64	0.7512	2.1371	0.0020	0.0033
**TOSIS_EARLY_G1**		65		41	16
**MORI_LARGE_PRE_BII_LYMPHOCYTE_UP**	84	0.7499	2.1372	<0.000	0.0034
		99	03	1	05
**GARY_CD5_TARGETS_DN**	414	0.6110	2.1341	<0.000	0.0034
		16	17	1	63
**HU_ANGIOGENESIS_DN**	35	0.7314	2.1333	<0.000	0.0034
		04	88	1	66
**REACTOME_ACTIVATION_OF_NF_KAPPAB_IN_B_CELLS**	61	0.6722	2.1235	0.0019	0.0035
		29	45	92	22
**BLUM_RESPONSE_TO_SALIRASIB_DN**	335	0.5825	2.1236	<0.000	0.0035
		35	08	1	97
**REACTOME_AUTODEGRADATION_OF_CDH1_BY_CDH1_APC_C**	56	0.7660	2.1249	<0.000	0.0036
		16	62	1	4
**WANG_RESPONSE_TO_GSK3_INHIBITOR_SB216763_DN**	332	0.5614	2.1239	<0.000	0.0036
		21	75	1	57
**WONG_EMBRYONIC_STEM_CELL_CORE**	328	0.6972	2.1271	<0.000	0.0036
		58	94	1	96
**REACTOME_APC_C_CDC20_MEDIATED_DEGRADATION_OF_MITOTIC_PROTEINS**	65	0.7552	2.1288	0.0020	0.0037
		11	87	41	14
**SCHLOSSER_MYC_TARGETS_REPRESSED_BY_SERUM**	152	0.6964	2.1253	<0.000	0.0037

		08	85	1	23
**REACTOME_MITOTIC_G1_G1_S_PHASES**	130	0.6892	2.1285	0.0020	0.0037
		45	44	16	46
**SESTO_RESPONSE_TO_UV_C0**	105	0.5843	2.1141	<0.000	0.0039
		36	59	1	24
**SHAFFER_IRF4_TARGETS_IN_ACTIVATED_B_LYMPHOCYTE**	76	0.6004	2.1135	0.0019	0.0039
		64	73	84	29
**DITTMER_PTHLH_TARGETS_UP**	110	0.5337	2.1147	<0.000	0.0039
		07	9	1	41
**REACTOME_HOST_INTERACTIONS_OF_HIV_FACTORS**	117	0.6038	2.1124	0.0039	0.0039
		94	7	92	68
**REACTOME_CYCLIN_E_ASSOCIATED_EVENTS_DURING_G1_S_TRANSITION_**	62	0.7504	2.1156	<0.000	0.0040
		4	05	1	21
**SHEDDEN_LUNG_CANCER_POOR_SURVIVAL_A6**	426	0.6366	2.1069	<0.000	0.0042
		68	05	1	72
**RUIZ_TNC_TARGETS_DN**	136	0.6282	2.1031	<0.000	0.0046
		49	1	1	41
**REACTOME_CELL_CYCLE_CHECKPOINTS**	111	0.7141	2.1001	0.0020	0.0046
		72	5	37	76
**RAMALHO_STEMNESS_UP**	196	0.5814	2.1007	<0.000	0.0047
		86	38	1	2
**REACTOME_DOWNSTREAM_SIGNALING_EVENTS_OF_B_CELL_RECEPTOR_BCR**	92	0.5668	2.0962	0.0041	0.0047
		38	36	07	6
**MOREAUX_MULTIPLE_MYELOMA_BY_TACI_DN**	154	0.6368	2.0949	<0.000	0.0047
		46	92	1	65
**MORI_MATURE_B_LYMPHOCYTE_DN**	71	0.5876	2.0964	<0.000	0.0048
		15	18	1	16
**YAO_TEMPORAL_RESPONSE_TO_PROGESTERONE_CLUSTER_17**	170	0.6794	2.0969	0.0021	0.0048
		58	8	1	99
**SANA_RESPONSE_TO_IFNG_DN**	81	0.6166	2.0889	<0.000	0.0051
		21	19	1	52
**MEINHOLD_OVARIAN_CANCER_LOW_GRADE_DN**	20	0.7828	2.0895	<0.000	0.0051

		43	73	1	56
**REACTOME_MITOTIC_M_M_G1_PHASES**	160	0.6877	2.0840	<0.000	0.0052
		35	1	1	41
**CHIANG_LIVER_CANCER_SUBCLASS_PROLIFERATION_UP**	164	0.5707	2.0869	0.0018	0.0052
		53	67	48	85
**REACTOME_DNA_REPLICATION**	180	0.6892	2.0863	<0.000	0.0052
		62	03	1	87
**GRAHAM_CML_DIVIDING_VS_NORMAL_QUIESCENT_UP**	176	0.6630	2.0825	<0.000	0.0053
		97	55	1	01
**HOLLEMAN_ASPARAGINASE_RESISTANCE_ALL_DN**	22	0.7660	2.0840	<0.000	0.0053
		79	2	1	19
**OLSSON_E2F3_TARGETS_DN**	45	0.6177	2.0845	<0.000	0.0053
		24	62	1	66
**FOURNIER_ACINAR_DEVELOPMENT_LATE_2**	265	0.6024	2.0795	<0.000	0.0055
		08	96	1	97
**REACTOME_APOPTOSIS**	141	0.5207	2.0750	<0.000	0.0056
		86	1	1	33
**REACTOME_REGULATION_OF_APOPTOSIS**	56	0.6839	2.0778	0.0041	0.0056
		59	1	49	54
**PAL_PRMT5_TARGETS_UP**	195	0.5331	2.0753	0.0020	0.0056
		25	76	12	87
**REACTOME_S_PHASE**	106	0.7213	2.0756	0.0020	0.0057
		56	82	37	25
**BIOCARTA_PROTEASOME_PATHWAY**	28	0.8488	2.0778	<0.000	0.0057
		03	53	1	34
**GARCIA_TARGETS_OF_FLI1_AND_DAX1_DN**	162	0.5706	2.0633	<0.000	0.0058
		94	92	1	22
**CAFFAREL_RESPONSE_TO_THC_24HR_5_DN**	55	0.6149	2.0622	<0.000	0.0058
		52	24	1	39
**UDAYAKUMAR_MED1_TARGETS_UP**	129	0.5340	2.0642	<0.000	0.0058
		9	01	1	61
**SCHLOSSER_MYC_TARGETS_AND_SERUM_RESPONSE_DN**	45	0.7209	2.0613	<0.000	0.0058

		78	38	1	81
**WANG_TUMOR_INVASIVENESS_UP**	362	0.4793	2.0644	<0.000	0.0059
		47	17	1	21
**REACTOME_CROSS_PRESENTATION_OF_SOLUBLE_EXOGENOUS_ANTIGENS_ENDOSOMES**	47	0.7242	2.0682	0.0040	0.0059
		03	43	82	28
**WINNEPENNINCKX_MELANOMA_METASTASIS_UP**	151	0.7119	2.0686	<0.000	0.0059
		04	1	1	84
**YAO_TEMPORAL_RESPONSE_TO_PROGESTERONE_CLUSTER_13**	164	0.7016	2.0644	<0.000	0.0059
		22	29	1	94
**KEGG_PROTEASOME**	42	0.7698	2.0650	<0.000	0.0060
		29	72	1	17
**HOFFMANN_LARGE_TO_SMALL_PRE_BII_LYMPHOCYTE_UP**	157	0.6502	2.0700	0.0019	0.0060
		02	5	38	24
**SCIBETTA_KDM5B_TARGETS_DN**	77	0.5732	2.0687	<0.000	0.0060
		37	2	1	62
**REACTOME_METABOLISM_OF_PROTEINS**	414	0.5525	2.0653	<0.000	0.0060
		98	03	1	92
**REACTOME_G1_S_TRANSITION**	106	0.7103	2.0554	0.0040	0.0063
		94	16	57	86
**GUTIERREZ_MULTIPLE_MYELOMA_UP**	29	0.7297	2.0560	<0.000	0.0064
		11	22	1	19
**YAMAZAKI_TCEB3_TARGETS_DN**	201	0.4775	2.0539	<0.000	0.0064
		71	12	1	91
**REACTOME_SCFSKP2_MEDIATED_DEGRADATION_OF_P27_P21**	53	0.7619	2.0502	<0.000	0.0065
		61	8	1	35
**ROME_INSULIN_TARGETS_IN_MUSCLE_UP**	407	0.4965	2.0505	<0.000	0.0065
		87	54	1	76
**REACTOME_P53_DEPENDENT_G1_DNA_DAMAGE_RESPONSE**	53	0.7170	2.0523	0.0020	0.0065
		89	45	53	93
**GOLDRATH_HOMEOSTATIC_PROLIFERATION**	163	0.5386	2.0518	<0.000	0.0066
		17	26	1	19
**SARRIO_EPITHELIAL_MESENCHYMAL_TRANSITION_UP**	170	0.6166	2.0506	<0.000	0.0066

		01	47	1	23
**BOYAULT_LIVER_CANCER_SUBCLASS_G23_UP**	49	0.6945	2.0466	<0.000	0.0066
		69	81	1	47
**REACTOME_TRANSCRIPTION**	187	0.5976	2.0450	<0.000	0.0066
		1	12	1	49
**KOBAYASHI_EGFR_SIGNALING_24HR_DN**	242	0.6931	2.0456	0.0019	0.0066
		45	29	92	84
**BOYAULT_LIVER_CANCER_SUBCLASS_G123_UP**	44	0.6758	2.0437	<0.000	0.0066
		67	83	1	88
**KEGG_RNA_DEGRADATION**	55	0.6270	2.0507	<0.000	0.0066
		28	14	1	95
**ELVIDGE_HYPOXIA_DN**	136	0.5426	2.0469	0.0020	0.0067
		37	46	7	05
**IVANOVA_HEMATOPOIESIS_INTERMEDIATE_PROGENITOR**	140	0.5298	2.0482	<0.000	0.0067
		9	28	1	17
**WINTER_HYPOXIA_UP**	88	0.5877	2.0429	0.0020	0.0067
		9	82	41	38
**CONCANNON_APOPTOSIS_BY_EPOXOMICIN_DN**	159	0.4774	2.0418	<0.000	0.0067
		01	22	1	63
**PROVENZANI_METASTASIS_UP**	183	0.4868	2.0471	<0.000	0.0067
		34	18	1	66
**BHATTACHARYA_EMBRYONIC_STEM_CELL**	86	0.6060	2.0399	<0.000	0.0067
		62	67	1	66
**MORI_IMMATURE_B_LYMPHOCYTE_DN**	90	0.7192	2.0402	0.0019	0.0067
		18	78	8	91
**REACTOME_AUTODEGRADATION_OF_THE_E3_UBIQUITIN_LIGASE_COP1**	47	0.7370	2.0391	0.0060	0.0068
		72	97	61	08
**BASAKI_YBX1_TARGETS_UP**	268	0.5641	2.0371	0.0020	0.0069
		78	55	37	01
**SCHUHMACHER_MYC_TARGETS_UP**	77	0.6850	2.0331	0.0020	0.0069
		95	07	41	15
**REACTOME_CDT1_ASSOCIATION_WITH_THE_CDC6_ORC_ORIGIN_COMPLEX**	54	0.7433	2.0332	0.0040	0.0069

		35	69	98	61
**MOREAUX_B_LYMPHOCYTE_MATURATION_BY_TACI_DN**	66	0.6901	2.0364	<0.000	0.0069
		55	27	1	61
**REACTOME_SYNTHESIS_OF_DNA**	90	0.7179	2.0371	0.0060	0.0069
		51	88	48	65
**REACTOME_ORC1_REMOVAL_FROM_CHROMATIN**	65	0.7264	2.0360	0.0041	0.0069
		14	23	67	65
**REACTOME_P53_INDEPENDENT_G1_S_DNA_DAMAGE_CHECKPOINT**	48	0.7477	2.0337	0.0040	0.0069
		99	85	82	81
**FERRANDO_T_ALL_WITH_MLL_ENL_FUSION_DN**	83	0.5713	2.0351	0.0019	0.0070
		67	33	88	1
**WONG_MITOCHONDRIA_GENE_MODULE**	216	0.6824	2.0346	0.0042	0.0070
		82	9	64	1
**REACTOME_PERK_REGULATED_GENE_EXPRESSION**	26	0.6693	2.0338	0.0019	0.0070
		76	56	61	42
**REACTOME_SCF_BETA_TRCP_MEDIATED_DEGRADATION_OF_EMI1**	49	0.7459	2.0283	0.0040	0.0071
		22	53	73	27
**YU_MYC_TARGETS_UP**	39	0.8093	2.0287	<0.000	0.0071
		54	94	1	78
**MUELLER_PLURINET**	293	0.5980	2.0302	<0.000	0.0071
		35	95	1	85
**REACTOME_REGULATION_OF_ORNITHINE_DECARBOXYLASE_ODC**	48	0.7462	2.0291	0.0061	0.0071
		3	63	98	96
**CAIRO_PML_TARGETS_BOUND_BY_MYC_UP**	23	0.7005	2.0275	0.0020	0.0072
		41	2	2	06
**KAAB_FAILED_HEART_ATRIUM_DN**	136	0.5234	2.0292	0.0040	0.0072
		81	25	57	5
**CHICAS_RB1_TARGETS_LOW_SERUM**	84	0.5676	2.0260	<0.000	0.0073
			4	1	29
**REACTOME_ANTIGEN_PROCESSING_UBIQUITINATION_PROTEASOME_DEGRADATION**	191	0.4958	2.0234	0.0020	0.0075
		41	37	12	6
**MORI_PRE_BI_LYMPHOCYTE_UP**	77	0.6485	2.0189	<0.000	0.0076

		59	02	1	96
**WHITEFORD_PEDIATRIC_CANCER_MARKERS**	110	0.6963	2.0220	0.0039	0.0077
		85	35	76	
**REACTOME_DESTABILIZATION_OF_MRNA_BY_AUF1_HNRNP_D0**	50	0.7301	2.0213	0.0040	0.0077
		71	82	9	1
**REACTOME_VIF_MEDIATED_DEGRADATION_OF_APOBEC3G**	47	0.7475	2.0190	0.0040	0.0077
		84	15	9	47
**ELLWOOD_MYC_TARGETS_DN**	38	0.5973	2.0193	0.0019	0.0077
		04	29	72	96
**MOOTHA_HUMAN_MITODB_6_2002**	413	0.5945	2.0194	0.0042	0.0078
		84	72	46	43
**REACTOME_ASSEMBLY_OF_THE_PRE_REPLICATIVE_COMPLEX**	63	0.7331	2.0143	0.0062	0.0081
		28	63	5	3
**DANG_MYC_TARGETS_UP**	138	0.6476	2.0125	<0.000	0.0082
		78	99	1	44
**MOHANKUMAR_HOXA1_TARGETS_UP**	384	0.4529	2.0078	<0.000	0.0085
		9	21	1	75
**ZAMORA_NOS2_TARGETS_UP**	63	0.6129	2.0062	<0.000	0.0086
		06	2	1	99
**MENSSEN_MYC_TARGETS**	51	0.6863	2.0038	<0.000	0.0087
		76	8	1	76
**MANALO_HYPOXIA_DN**	271	0.6614	2.0031	0.0041	0.0087
		13	2	15	76
**KEGG_PYRIMIDINE_METABOLISM**	95	0.5620	2.0051	<0.000	0.0087
		94	24	1	84
**WEST_ADRENOCORTICAL_TUMOR_MARKERS_UP**	21	0.7115	2.0040	<0.000	0.0088
		54	3	1	28
**KIM_MYC_AMPLIFICATION_TARGETS_UP**	186	0.5023	2.0013	<0.000	0.0089
		72	77	1	4
**REACTOME_DEADENYLATION_DEPENDENT_MRNA_DECAY**	42	0.6240	2.0001	<0.000	0.0090
		76	73	1	76
**SASAKI_ADULT_T_CELL_LEUKEMIA**	168	0.5032	1.9987	<0.000	0.0091

		24	95	1	34
**REACTOME_METABOLISM_OF_NUCLEOTIDES**	69	0.5783	1.9950	<0.000	0.0093
		58	1	1	55
**BLALOCK_ALZHEIMERS_DISEASE_INCIPIENT_DN**	160	0.4658	1.9956	<0.000	0.0093
		76	35	1	57
**REACTOME_METABOLISM_OF_RNA**	252	0.6298	1.9957	0.0042	0.0094
		7	42	11	17
**APPIERTO_RESPONSE_TO_FENRETINIDE_DN**	50	0.5683	1.9929	0.0020	0.0095
		75	79	66	63
**HU_GENOTOXIC_DAMAGE_4HR**	35	0.6509	1.9922	0.0041	0.0095
		31	32	15	72
**PELLICCIOTTA_HDAC_IN_ANTIGEN_PRESENTATION_UP**	62	0.6555	1.9912	0.0020	0.0095
		32	73	41	83
**SAKAI_TUMOR_INFILTRATING_MONOCYTES_DN**	78	0.6088	1.9916	0.0019	0.0095
		74	92	57	91
**VANTVEER_BREAST_CANCER_METASTASIS_DN**	111	0.6029	1.9903	<0.000	0.0096
		6	11	1	62
**WILCOX_RESPONSE_TO_PROGESTERONE_UP**	140	0.5642	1.9890	<0.000	0.0097
		48	76	1	11
**MOOTHA_PGC**	400	0.5071	1.9876	0.0041	0.0098
		9	53	24	16
**REACTOME_CLASS_I_MHC_MEDIATED_ANTIGEN_PROCESSING_PRESENTATION**	228	0.4751	1.9842	<0.000	0.0101
		94	83	1	88
**BURTON_ADIPOGENESIS_5**	114	0.5986	1.9789	0.0062	0.0108
		54	54	63	03
**RHODES_UNDIFFERENTIATED_CANCER**	67	0.7431	1.9795	0.0020	0.0108
		32	29	62	09
**CAFFAREL_RESPONSE_TO_THC_24HR_5_UP**	30	0.6527	1.9795	0.0040	0.0108
		32	87	16	69
**HEDENFALK_BREAST_CANCER_HEREDITARY_VS_SPORADIC**	46	0.5600	1.9769	<0.000	0.0109
		09	86	1	64
**FUJII_YBX1_TARGETS_DN**	193	0.6009	1.9755	0.0038	0.0110

		49	13	99	59
**ZHANG_RESPONSE_TO_CANTHARIDIN_DN**	67	0.6717	1.9694	<0.000	0.0119
		93	64	1	87
**SEIDEN_MET_SIGNALING**	18	0.8040	1.9620	<0.000	0.0120
		33	17	1	07
**GAJATE_RESPONSE_TO_TRABECTEDIN_DN**	18	0.6621	1.9621	<0.000	0.0120
		37	14	1	45
**LEE_EARLY_T_LYMPHOCYTE_UP**	96	0.6504	1.9686	0.0020	0.0120
		73	45	37	73
**YAO_TEMPORAL_RESPONSE_TO_PROGESTERONE_CLUSTER_11**	95	0.5511	1.9621	0.0020	0.0121
		54	71	12	03
**HU_GENOTOXIN_ACTION_DIRECT_VS_INDIRECT_24HR**	51	0.5268	1.9641	<0.000	0.0121
		78	72	1	37
**BYSTRYKH_HEMATOPOIESIS_STEM_CELL_AND_BRAIN_QTL_CIS**	57	0.5269	1.9623	0.0021	0.0121
		24	84	69	41
**ELVIDGE_HIF1A_TARGETS_UP**	63	0.5458	1.9627	0.0020	0.0121
		9	5	16	46
**MOOTHA_MITOCHONDRIA**	426	0.5808	1.9669	0.0043	0.0121
		08	18	01	53
**REACTOME_ASPARAGINE_N_LINKED_GLYCOSYLATION**	80	0.5440	1.9675	<0.000	0.0121
		85	55	1	63
**YAO_TEMPORAL_RESPONSE_TO_PROGESTERONE_CLUSTER_14**	134	0.5626	1.9629	<0.000	0.0121
		53	78	1	69
**REACTOME_CDK_MEDIATED_PHOSPHORYLATION_AND_REMOVAL_OF_CDC6**	46	0.7362	1.9643	0.0082	0.0121
		17	76	3	7
**ODONNELL_TFRC_TARGETS_DN**	120	0.6120	1.9659	0.0059	0.0121
		59	91	52	85
**GOTZMANN_EPITHELIAL_TO_MESENCHYMAL_TRANSITION_DN**	198	0.4658	1.9631	<0.000	0.0122
		09	17	1	13
**REACTOME_TRNA_AMINOACYLATION**	42	0.6982	1.9634	<0.000	0.0122
		1	79	1	17
**CHOI_ATL_STAGE_PREDICTOR**	38	0.6158	1.9649	0.0038	0.0122

		15	95	17	33
**OUELLET_CULTURED_OVARIAN_CANCER_INVASIVE_VS_LMP_UP**	65	0.5387	1.9644	<0.000	0.0122
		69	09	1	43
**HEDENFALK_BREAST_CANCER_BRCA1_VS_BRCA2**	156	0.4688	1.9591	<0.000	0.0124
		7	79	1	05
**REACTOME_M_G1_TRANSITION**	78	0.7085	1.9582	0.0062	0.0124
		57	69	11	66
**SHIPP_DLBCL_VS_FOLLICULAR_LYMPHOMA_UP**	44	0.7104	1.9551	0.0040	0.0128
		74	14	9	43
**REACTOME_MITOTIC_PROMETAPHASE**	78	0.6660	1.9548	0.0019	0.0128
		76	65	88	51
**SOTIRIOU_BREAST_CANCER_GRADE_1_VS_3_UP**	141	0.7582	1.9554	0.0020	0.0128
		72	09	16	6
**WHITFIELD_CELL_CYCLE_G2_M**	194	0.4778	1.9543	0.0019	0.0128
		55	39	53	91
**GREENBAUM_E2A_TARGETS_UP**	32	0.7446	1.9555	<0.000	0.0129
		05	43	1	04
**HONMA_DOCETAXEL_RESISTANCE**	30	0.7223	1.9538	<0.000	0.0129
		34	2	1	12
**ROSTY_CERVICAL_CANCER_PROLIFERATION_CLUSTER**	133	0.7390	1.9499	0.0079	0.0132
		69	99	21	76
**DEN_INTERACT_WITH_LCA5**	25	0.6729	1.9513	0.0020	0.0132
		56	31	16	94
**REACTOME_ANTIGEN_PROCESSING_CROSS_PRESENTATION**	72	0.6279	1.9491	0.0082	0.0133
		35	84	14	03
**DANG_REGULATED_BY_MYC_UP**	71	0.5750	1.9509	0.0041	0.0133
		79	62	41	04
**REACTOME_G1_PHASE**	35	0.6082	1.9501	<0.000	0.0133
		04	57	1	35
**REACTOME_SYNTHESIS_AND_INTERCONVERSION_OF_NUCLEOTIDE_DI_AND_TRIPHOSPHATES**	18	0.7253	1.9463	0.0020	0.0137
		51	77	75	93
**NAKAMURA_CANCER_MICROENVIRONMENT_DN**	43	0.6833	1.9454	0.0039	0.0138

		99	81	68	52
**MISSIAGLIA_REGULATED_BY_METHYLATION_DN**	115	0.6740	1.9436	0.0020	0.0140
		56	69	24	07
**KEGG_CELL_CYCLE**	124	0.5604	1.9441	0.0040	0.0140
		41	24	49	13
**REACTOME_LATE_PHASE_OF_HIV_LIFE_CYCLE**	92	0.5720	1.9395	0.0020	0.0147
		5	26	28	72
**LE_EGR2_TARGETS_UP**	104	0.5786	1.9389	0.0039	0.0147
		57	54	6	97
**DAZARD_RESPONSE_TO_UV_SCC_UP**	111	0.6118	1.9376	<0.000	0.0149
		07	32	1	73
**ZHOU_CELL_CYCLE_GENES_IN_IR_RESPONSE_24HR**	119	0.6850	1.9370	0.0059	0.015
		97	31	52	
**BIDUS_METASTASIS_UP**	203	0.6296	1.9367	0.0039	0.0150
		5	6	68	12
**JUBAN_TARGETS_OF_SPI1_AND_FLI1_DN**	84	0.5071	1.9354	<0.000	0.0151
		28	73	1	65
**PID_PLK1_PATHWAY**	43	0.6460	1.9339	0.0019	0.0153
		85	2	61	69
**MOLENAAR_TARGETS_OF_CCND1_AND_CDK4_DN**	51	0.7155	1.9342	<0.000	0.0153
		45	41	1	92
**REACTOME_HIV_LIFE_CYCLE**	105	0.5641	1.9332	<0.000	0.0154
		78	21	1	17
**YU_BAP1_TARGETS**	29	0.6879	1.9308	0.0020	0.0157
		2	48	62	6
**BENPORATH_ES_1**	351	0.4955	1.9301	<0.000	0.0158
		48	9	1	15
**SHEPARD_CRUSH_AND_BURN_MUTANT_UP**	185	0.4720	1.9295	<0.000	0.0158
		57	26	1	8
**PRAMOONJAGO_SOX4_TARGETS_DN**	50	0.6012	1.9284	0.0020	0.0160
		12	66	75	54
**KEGG_HUNTINGTONS_DISEASE**	168	0.5485	1.9248	0.0083	0.0161

		4	57	68	28
**PELLICCIOTTA_HDAC_IN_ANTIGEN_PRESENTATION_DN**	49	0.6579	1.9277	0.0058	0.0161
		77	98	94	28
**LANDIS_ERBB2_BREAST_TUMORS_324_UP**	139	0.4894	1.9250	0.0019	0.0161
		59	03	65	85
**ZUCCHI_METASTASIS_UP**	38	0.5719	1.9267	0.0019	0.0161
		76	55	76	96
**MULLIGAN_NTF3_SIGNALING_VIA_INSR_AND_IGF1R_UP**	23	0.6420	1.9252	0.0058	0.0162
		71	04	03	16
**ZHANG_BREAST_CANCER_PROGENITORS_UP**	399	0.5846	1.9261	0.0039	0.0162
		75	2	37	39
**CHANG_CYCLING_GENES**	138	0.6644	1.9253	0.0098	0.0162
		15	14	23	75
**REACTOME_METABOLISM_OF_MRNA**	208	0.6166	1.9226	0.0104	0.0164
		06	27	6	09
**REACTOME_METABOLISM_OF_AMINO_ACIDS_AND_DERIVATIVES**	186	0.4978	1.9220	0.0020	0.0164
		57	98	79	39
**WANG_CLIM2_TARGETS_DN**	176	0.5160	1.9227	<0.000	0.0164
		05	86	1	49
**MARKEY_RB1_ACUTE_LOF_UP**	223	0.5642	1.9213	0.0040	0.0164
		59	45	24	84
**KANG_DOXORUBICIN_RESISTANCE_UP**	50	0.7852	1.9199	0.0019	0.0166
		66	47	49	94
**WAMUNYOKOLI_OVARIAN_CANCER_GRADES_1_2_UP**	134	0.5858	1.9186	0.0157	0.0168
		58	78	48	
**REACTOME_CHROMOSOME_MAINTENANCE**	110	0.6578	1.9186	0.0019	0.0168
		94	88	53	77
**KEGG_ONE_CARBON_POOL_BY_FOLATE**	17	0.7174	1.9169	0.0020	0.0169
		29	35	41	98
**BIOCARTA_MPR_PATHWAY**	33	0.5830	1.9171	<0.000	0.0170
		2	74	1	17
**FURUKAWA_DUSP6_TARGETS_PCI35_DN**	67	0.6184	1.9158	0.0019	0.0171

		39	13	84	39
**REACTOME_APC_CDC20_MEDIATED_DEGRADATION_OF_NEK2A**	21	0.7563	1.9141	<0.000	0.0174
		16	45	1	75
**MELLMAN_TUT1_TARGETS_UP**	18	0.6420	1.9115	0.0041	0.0179
		19	05	41	82
**ODONNELL_TARGETS_OF_MYC_AND_TFRC_DN**	43	0.7468	1.9107	<0.000	0.0180
		81	81	1	48
**PID_MYC_ACTIV_PATHWAY**	78	0.5523	1.9098	0.0019	0.0180
		4	27	53	83
**HSIAO_HOUSEKEEPING_GENES**	384	0.5485	1.9100	0.0122	0.0181
		1	66	45	19
**MARTINEZ_RESPONSE_TO_TRABECTEDIN_UP**	67	0.5498	1.9093	0.0021	0.0181
		75	69	05	25
**HESS_TARGETS_OF_HOXA9_AND_MEIS1_UP**	63	0.5566	1.9060	0.0042	0.0186
		47	96	11	99
**REACTOME_ACTIVATION_OF_GENES_BY_ATF4**	23	0.6452	1.9026	0.0059	0.0193
		25	47	76	94
**REACTOME_RNA_POL_I_RNA_POL_III_AND_MITOCHONDRIAL_TRANSCRIPTION**	109	0.6394	1.9020	0.0295	0.0194
		92	4	86	17
**REACTOME_INHIBITION_OF_THE_PROTEOLYTIC_ACTIVITY_OF_APC_C_REQUIRED_FOR_THE_ONSET_OF_ANAPHASE_BY_MITOTI**	18	0.7532	1.9006	<0.000	0.0196
**C_SPINDLE_CHECKPOINT_COMPONENTS**		97	52	1	72
**SONG_TARGETS_OF_IE86_CMV_PROTEIN**	58	0.7125	1.8991	0.0060	0.0199
		31	93	98	45
**BURTON_ADIPOGENESIS_3**	100	0.6421	1.8960	0.0155	0.0201
		53	54	04	36
**GRAHAM_NORMAL_QUIESCENT_VS_NORMAL_DIVIDING_DN**	85	0.7212	1.8971	0.0080	0.0201
		76	68	65	37
**GRADE_METASTASIS_DN**	42	0.6560	1.8957	0.0099	0.0201
		26	28	8	48
**JIANG_HYPOXIA_VIA_VHL**	33	0.5872	1.8963	<0.000	0.0201
		76	54	1	55
**DAIRKEE_CANCER_PRONE_RESPONSE_BPA**	49	0.5443	1.8972	0.0021	0.0201

		21	54	28	99
**CHUNG_BLISTER_CYTOTOXICITY_UP**	123	0.5033	1.8964	0.0120	0.0202
		64	45	24	26
**GAVIN_FOXP3_TARGETS_CLUSTER_P6**	88	0.5733	1.8973	0.0118	0.0202
		89	59	58	66
**TIEN_INTESTINE_PROBIOTICS_24HR_DN**	210	0.4886	1.8935	0.0077	0.0204
		25	37	82	1
**KEGG_AMINOACYL_TRNA_BIOSYNTHESIS**	41	0.6633	1.8936	<0.000	0.0204
		49	83	1	34
**STONER_ESOPHAGEAL_CARCINOGENESIS_UP**	36	0.5606	1.8938	<0.000	0.0204
		43	74	1	81
**REACTOME_TELOMERE_MAINTENANCE**	71	0.7246	1.8905	0.0078	0.0207
		26	31	59	23
**ZHAN_V2_LATE_DIFFERENTIATION_GENES**	42	0.5709	1.8915	0.0020	0.0207
		03	39	33	61
**REACTOME_RNA_POL_II_PRE_TRANSCRIPTION_EVENTS**	51	0.5718	1.8909	0.0060	0.0207
		36	4	36	63
**THILLAINADESAN_ZNF217_TARGETS_UP**	42	0.5799	1.8906	0.0041	0.0207
		79	57	07	68
**CHNG_MULTIPLE_MYELOMA_HYPERPLOID_DN**	28	0.6261	1.8870	0.0059	0.0214
		96	81	17	25
**REACTOME_METABOLISM_OF_NON_CODING_RNA**	47	0.6628	1.8862	0.0081	0.0214
		05	68	14	26
**ZHANG_TLX_TARGETS_60HR_DN**	263	0.6176	1.8858	0.0120	0.0214
		01	95	48	52
**CROONQUIST_NRAS_SIGNALING_DN**	71	0.7253	1.8863	0.0120	0.0215
		23	38	48	05
**REACTOME_PHOSPHORYLATION_OF_THE_APC_C**	17	0.7435	1.8826	<0.000	0.0219
		28	63	1	38
**REACTOME_FORMATION_OF_TRANSCRIPTION_COUPLED_NER_TC_NER_REPAIR_COMPLEX**	28	0.6160	1.8836	0.0063	0.0219
		97	27	29	41
**FISCHER_G2_M_CELL_CYCLE**	220	0.5764	1.8832	0.0138	0.0219

		78	35	89	71
**REACTOME_ER_PHAGOSOME_PATHWAY**	58	0.6884	1.8828	0.0104	0.0219
		65	4	6	91
**REACTOME_RNA_POL_II_TRANSCRIPTION_PRE_INITIATION_AND_PROMOTER_OPENING**	39	0.6079	1.8806	0.0019	0.0221
		15	82	92	5
**REACTOME_MRNA_SPLICING_MINOR_PATHWAY**	40	0.6747	1.8807	0.0103	0.0222
		78	55	95	13
**REACTOME_CONVERSION_FROM_APC_C_CDC20_TO_APC_C_CDH1_IN_LATE_ANAPHASE**	16	0.7059	1.8808	0.002	0.0222
		24	06		97
**SESTO_RESPONSE_TO_UV_C7**	65	0.5471	1.8787	0.0059	0.0224
		51	28	41	99
**LANDIS_BREAST_CANCER_PROGRESSION_UP**	40	0.5905	1.8771	0.0019	0.0227
		33	69	96	89
**REACTOME_NUCLEOTIDE_EXCISION_REPAIR**	48	0.5976	1.8753	0.0060	0.0231
		07	45	85	22
**SUNG_METASTASIS_STROMA_DN**	49	0.5837	1.8755	0.0078	0.0231
		17	28	13	57
**SCHLOSSER_MYC_TARGETS_AND_SERUM_RESPONSE_UP**	46	0.6247	1.8711	0.0062	0.0234
		71	3	89	36
**ZHOU_TNF_SIGNALING_4HR**	52	0.5239	1.8713	0.0097	0.0234
		42	57	85	52
**PARK_HSC_AND_MULTIPOTENT_PROGENITORS**	49	0.5099	1.8728	0.0020	0.0235
		23	91	04	
**CHIARADONNA_NEOPLASTIC_TRANSFORMATION_KRAS_UP**	121	0.4724	1.8721	0.0019	0.0235
		19	82	31	1
**PEART_HDAC_PROLIFERATION_CLUSTER_DN**	73	0.5166	1.8714	0.0019	0.0235
		2	08	8	22
**SAKAI_CHRONIC_HEPATITIS_VS_LIVER_CANCER_UP**	79	0.6123	1.8730	<0.000	0.0235
		88	37	1	44
**REACTOME_APC_C_CDC20_MEDIATED_DEGRADATION_OF_CYCLIN_B**	19	0.7390	1.8722	<0.000	0.0235
		86	19	1	89
**REACTOME_TRANSCRIPTION_COUPLED_NER_TC_NER**	43	0.6172	1.8714	0.0041	0.0236

		36	98	15	01
**REACTOME_PROCESSING_OF_CAPPED_INTRONLESS_PRE_MRNA**	23	0.6895	1.8685	0.0059	0.0240
		46	57	64	35
**REACTOME_DNA_REPAIR**	101	0.5666	1.8663	0.0042	0.0241
		54	02	46	99
**WHITFIELD_CELL_CYCLE_M_G1**	139	0.4787	1.8670	0.0019	0.0242
		62	97	34	33
**KIM_WT1_TARGETS_DN**	426	0.4519	1.8673	0.0019	0.0242
		19	75	42	56
**BRACHAT_RESPONSE_TO_CAMPTOTHECIN_DN**	43	0.5660	1.8663	0.0039	0.0242
		5	46	76	84
**COLLER_MYC_TARGETS_UP**	25	0.7095	1.8654	0.0062	0.0242
		67	1	24	91
**VIETOR_IFRD1_TARGETS**	21	0.6384	1.8666	0.0039	0.0242
		51	67	53	94
**REACTOME_PROTEIN_FOLDING**	51	0.5299	1.8647	0.0038	0.0243
		93	29	61	04
**WANG_TARGETS_OF_MLL_CBP_FUSION_DN**	42	0.5334	1.8648	0.0040	0.0243
		49	43	9	51
**CROONQUIST_IL6_DEPRIVATION_DN**	96	0.6977	1.8632	0.0121	0.0244
		49	99	21	64
**WHITFIELD_CELL_CYCLE_LITERATURE**	44	0.7424	1.8634	0.0180	0.0245
		58		72	33
**REACTOME_PREFOLDIN_MEDIATED_TRANSFER_OF_SUBSTRATE_TO_CCT_TRIC**	27	0.6639	1.8625	0.0060	0.0246
		31	08	24	18
**HU_GENOTOXIC_DAMAGE_24HR**	32	0.6156	1.8621	0.0101	0.0246
		58	42	01	38
**KAYO_AGING_MUSCLE_DN**	117	0.4482	1.8601	<0.000	0.0250
		68	02	1	57
**POMEROY_MEDULLOBLASTOMA_PROGNOSIS_DN**	43	0.6394	1.8569	0.0040	0.0250
		52	3	57	58
**ALCALA_APOPTOSIS**	85	0.5084	1.8581	0.0101	0.0250

		04	29	42	78
**ZHOU_CELL_CYCLE_GENES_IN_IR_RESPONSE_6HR**	80	0.7269	1.8572	0.0061	0.0250
		25	22	35	79
**PID_RAC1_PATHWAY**	54	0.4853	1.8577	0.0057	0.0250
		11	98	03	85
**BHATI_G2M_ARREST_BY_2METHOXYESTRADIOL_UP**	108	0.4884	1.8584	<0.000	0.0250
		52	75	1	92
**BILANGES_RAPAMYCIN_SENSITIVE_VIA_TSC1_AND_TSC2**	71	0.6133	1.8574	0.0060	0.0250
		46	5	36	93
**JIANG_AGING_HYPOTHALAMUS_UP**	46	0.6046	1.8585	0.0042	0.0251
		01	45	02	57
**REACTOME_CYCLIN_A_B1_ASSOCIATED_EVENTS_DURING_G2_M_TRANSITION**	15	0.7906	1.8585	0.002	0.0252
		61	72		45
**RAHMAN_TP53_TARGETS_PHOSPHORYLATED**	21	0.7472	1.8589	<0.000	0.0252
		18	5	1	95
**BURTON_ADIPOGENESIS_PEAK_AT_24HR**	42	0.6925	1.8586	0.0042	0.0253
		97	02	02	23
**JAZAERI_BREAST_CANCER_BRCA1_VS_BRCA2_UP**	47	0.5215	1.8542	0.0020	0.0256
		73	2	16	08
**REACTOME_CYTOSOLIC_TRNA_AMINOACYLATION**	24	0.7239	1.8535	0.0039	0.0256
		65	47	45	87
**GAZDA_DIAMOND_BLACKFAN_ANEMIA_PROGENITOR_DN**	62	0.5558	1.8529	0.0040	0.0257
		98	53	08	62
**TOOKER_GEMCITABINE_RESISTANCE_DN**	119	0.4815	1.8517	0.0037	0.0258
		23	21	81	69
**DUTERTRE_ESTRADIOL_RESPONSE_24HR_UP**	309	0.5830	1.8519	0.0320	0.0259
		86	05	64	01
**MATTIOLI_MGUS_VS_PCL**	92	0.5301	1.8520	0.0081	0.0259
		57	63	8	32
**IVANOVA_HEMATOPOIESIS_EARLY_PROGENITOR**	481	0.3919	1.8508	<0.000	0.0259
		43	22	1	82
**REACTOME_MITOCHONDRIAL_PROTEIN_IMPORT**	48	0.6987	1.8510	0.0086	0.0260

		03	95	39	01
**KEGG_DNA_REPLICATION**	36	0.7221	1.8464	0.0059	0.0269
		6	46	41	9
**YOSHIMURA_MAPK8_TARGETS_DN**	347	0.3967	1.8459	0.0019	0.0270
		99	66	34	29
**KAUFFMANN_MELANOMA_RELAPSE_UP**	59	0.7403	1.8438	0.0040	0.0275
		9	58	82	08
**MMS_MOUSE_LYMPH_HIGH_4HRS_UP**	31	0.6539	1.8438	0.0019	0.0275
		19	65	92	92
**KEGG_OXIDATIVE_PHOSPHORYLATION**	114	0.6504	1.8432	0.0151	0.0276
		61	57	19	48
**VANOEVELEN_MYOGENESIS_SIN3A_TARGETS**	208	0.3987	1.8403	<0.000	0.0279
		23	39	1	32
**YAO_TEMPORAL_RESPONSE_TO_PROGESTERONE_CLUSTER_7**	72	0.4816	1.8415	0.0019	0.0279
		44	11	57	33
**WIERENGA_PML_INTERACTOME**	40	0.5255	1.8416	0.0019	0.0279
		91	88	72	61
**KEGG_NUCLEOTIDE_EXCISION_REPAIR**	44	0.5933	1.8404	0.0040	0.0279
		38	92	08	69
**BAE_BRCA1_TARGETS_UP**	70	0.4899	1.8410	0.0097	0.0279
		29	58	85	92
**TAKAO_RESPONSE_TO_UVB_RADIATION_DN**	95	0.4591	1.8406	0.0019	0.0280
		35	84	38	12
**REACTOME_MITOCHONDRIAL_TRNA_AMINOACYLATION**	21	0.7108	1.8418	<0.000	0.0280
		15	15	1	18
**ZHU_CMV_24_HR_UP**	93	0.5201	1.8371	0.0099	0.0282
		55	24	21	94
**KEGG_PARKINSONS_DISEASE**	109	0.6301	1.8371	0.0149	0.0283
		17	78	25	68
**KEGG_PURINE_METABOLISM**	150	0.4223	1.8373	<0.000	0.0284
		78	36	1	16
**TIEN_INTESTINE_PROBIOTICS_6HR_DN**	158	0.4490	1.8379	<0.000	0.0284

		92	04	1	32
AMUNDSON_GAMMA_RADIATION_RESPONSE	39	0.7337	1.8375	0.0081	0.0284
		38	12	47	65
IRITANI_MAD1_TARGETS_DN	46	0.6419	1.8379	0.01	0.0285
		06	49		09
KAMMINGA_EZH2_TARGETS	40	0.7609	1.8353	0.0038	0.0285
		08	1	76	5
REACTOME_BIOSYNTHESIS_OF_THE_N_GLYCAN_PRECURSOR_DOLICHOL_LIPID_LINKED_OLIGOSACCHARIDE_LLO_AND_TRANS	28	0.6273	1.8355	0.0040	0.0285
FER_TO_A_NASCENT_PROTEIN		45		49	95
WU_APOPTOSIS_BY_CDKN1A_VIA_TP53	53	0.6739	1.8348	0.0080	0.0285
		88	19	48	97
SMITH_LIVER_CANCER	42	0.5798	1.8356	0.0101	0.0286
		56	66	21	42
WELCSH_BRCA1_TARGETS_UP	192	0.4882	1.8343	0.0059	0.0286
		33	1	76	5
BLUM_RESPONSE_TO_SALIRASIB_UP	236	0.4402	1.8338	0.0100	0.0286
		71	89	2	93
REN_BOUND_BY_E2F	60	0.7136	1.8333	0.0081	0.0286
		38	19	3	97
REACTOME_PURINE_METABOLISM	32	0.5805	1.8334	0.0040	0.0287
		84	43	57	56
KEGG_OOCYTE_MEIOSIS	106	0.4453	1.8311	0.0018	0.0291
		33	26	73	23
NGO_MALIGNANT_GLIOMA_1P_LOH	15	0.7515	1.8304	0.0020	0.0291
		39	71	66	32
ZHAN_VARIABLE_EARLY_DIFFERENTIATION_GENES_DN	30	0.5751	1.8313	0.0020	0.0291
		53	27	79	54
BIOCARTA_CHREBP2_PATHWAY	39	0.5228	1.8306	0.0040	0.0291
		88	44	24	62
HOLLEMAN_PREDNISOLONE_RESISTANCE_B_ALL_UP	22	0.6239	1.8298	0.0079	0.0292
		92	15	68	35
REACTOME_TCA_CYCLE_AND_RESPIRATORY_ELECTRON_TRANSPORT	115	0.6552	1.8272	0.0173	0.0300

		46	3	91	14
**MORI_EMU_MYC_LYMPHOMA_BY_ONSET_TIME_UP**	99	0.5056	1.8256	0.0121	0.0300
		9	06	7	75
**FARMER_BREAST_CANCER_CLUSTER_2**	32	0.8048	1.8259	0.004	0.0300
		01	36		86
**DAIRKEE_CANCER_PRONE_RESPONSE_BPA_E2**	112	0.4567	1.8262	0.0039	0.0300
		36	03	84	94
**BIOCARTA_BCELLSURVIVAL_PATHWAY**	16	0.5917	1.8265	0.0058	0.0301
		53	1	25	09
**SEIDEN_ONCOGENESIS_BY_MET**	84	0.6710	1.8250	0.0096	0.0301
		88	49	15	18
**TONKS_TARGETS_OF_RUNX1_RUNX1T1_FUSION_MONOCYTE_UP**	195	0.4267	1.8239	<0.000	0.0302
		43	31	1	34
**SMITH_TERT_TARGETS_UP**	141	0.4523	1.8243	0.0137	0.0302
		91	42	25	48
**REACTOME_ENOS_ACTIVATION_AND_REGULATION**	19	0.6229	1.8216	0.0060	0.0308
		45	13	12	61
**REACTOME_TRANSLATION**	144	0.6596	1.8203	0.0258	0.0311
		47	84	96	35
**AFFAR_YY1_TARGETS_DN**	227	0.4451	1.8193	0.0020	0.0313
		71	66	45	82
**LI_AMPLIFIED_IN_LUNG_CANCER**	170	0.5344	1.8189	0.0105	0.0314
		73	5	26	73
**NADERI_BREAST_CANCER_PROGNOSIS_UP**	46	0.5823	1.8171	0.0145	0.0319
		61	92	53	75
**REACTOME_FORMATION_OF_TUBULIN_FOLDING_INTERMEDIATES_BY_CCT_TRIC**	21	0.6717	1.8167	0.0079	0.0320
		39	08	21	4
**REACTOME_RESPIRATORY_ELECTRON_TRANSPORT**	64	0.7569	1.8156	0.0087	0.0321
		11	02	72	26
**KEGG_ALZHEIMERS_DISEASE**	154	0.5136	1.8159	0.0126	0.0321
		25	09	32	43
**GOLUB_ALL_VS_AML_UP**	24	0.5770	1.8144	0.0062	0.0322

		33	99	24	92
**KUROKAWA_LIVER_CANCER_CHEMOTHERAPY_DN**	38	0.5264	1.8147	<0.000	0.0323
		65	42	1	14
**WATANABE_RECTAL_CANCER_RADIOTHERAPY_RESPONSIVE_UP**	103	0.4717	1.8117	0.0041	0.0330
		54	31	67	89
**TIEN_INTESTINE_PROBIOTICS_2HR_UP**	27	0.6133	1.8091	0.0078	0.0338
		52	59	28	1
**PUJANA_BRCA_CENTERED_NETWORK**	114	0.6306	1.8085	0.0119	0.0339
		73	13	05	04
**FLORIO_NEOCORTEX_BASAL_RADIAL_GLIA_DN**	187	0.5769	1.8057	0.0275	0.0345
		32	83	59	4
**WU_HBX_TARGETS_2_UP**	22	0.5948	1.8059	0.0063	0.0345
		66	18	83	86
**YAO_TEMPORAL_RESPONSE_TO_PROGESTERONE_CLUSTER_12**	77	0.5006	1.8052	0.0085	0.0346
		42	68	11	14
**AKL_HTLV1_INFECTION_UP**	24	0.5873	1.8044	0.0124	0.0348
		52	55	74	19
**MOOTHA_VOXPHOS**	83	0.7265	1.8037	0.0110	0.0348
		96	59	38	55
**MARTINEZ_RESPONSE_TO_TRABECTEDIN_DN**	265	0.4953	1.8038	0.0038	0.0348
		14	96	91	95
**REACTOME_MHC_CLASS_II_ANTIGEN_PRESENTATION**	87	0.4646	1.8026	0.0096	0.0351
		02	77	53	54
**ZHAN_MULTIPLE_MYELOMA_PR_UP**	43	0.7585	1.8018	0.0177	0.0352
		63	42	51	01
**TANG_SENESCENCE_TP53_TARGETS_DN**	54	0.6321	1.8020	0.0274	0.0352
		62	75	51	41
**SIMBULAN_PARP1_TARGETS_DN**	17	0.7134	1.7995	0.0080	0.0357
		25	93	16	2
**KEGG_BIOSYNTHESIS_OF_UNSATURATED_FATTY_ACIDS**	20	0.6262	1.7998	0.0041	0.0357
		96	17	49	55
**MARIADASON_RESPONSE_TO_CURCUMIN_SULINDAC_5**	21	0.5975	1.7974	0.0122	0.0358

		93	33	7	19
**MITSIADES_RESPONSE_TO_APLIDIN_DN**	239	0.5194	1.7984	0.0190	0.0358
		1	35	84	7
**REACTOME_CHOLESTEROL_BIOSYNTHESIS**	20	0.8081	1.7975	0.0041	0.0358
		33	39	58	82
**REACTOME_ASSOCIATION_OF_TRIC_CCT_WITH_TARGET_PROTEINS_DURING_BIOSYNTHESIS**	26	0.5673	1.7985	0.0114	0.0359
		71	86	72	12
**KOKKINAKIS_METHIONINE_DEPRIVATION_96HR_DN**	75	0.4793	1.7977	0.0080	0.0359
		08	82	32	14
**BIOCARTA_ACTINY_PATHWAY**	20	0.6369	1.7979	0.0099	0.0359
		89	32	6	64
**BOUDOUKHA_BOUND_BY_IGF2BP2**	103	0.4620	1.7966	0.0082	0.0359
		79	78	64	7
**REACTOME_RESPIRATORY_ELECTRON_TRANSPORT_ATP_SYNTHESIS_BY_CHEMIOSMOTIC_COUPLING_AND_HEAT_PRODUCTIO**	79	0.7234	1.7957	0.0129	0.0361
**N_BY_UNCOUPLING_PROTEINS_**		52	27	59	12
**WANG_METASTASIS_OF_BREAST_CANCER_ESR1_UP**	19	0.6877	1.7958	0.0097	0.0361
		62	2	66	71
**SHAFFER_IRF4_TARGETS_IN_MYELOMA_VS_MATURE_B_LYMPHOCYTE**	99	0.4842	1.7938	0.006	0.0366
		45	91		24
**EGUCHI_CELL_CYCLE_RB1_TARGETS**	23	0.8302	1.7923	0.0039	0.0367
		2	16	29	91
**REACTOME_DEPOSITION_OF_NEW_CENPA_CONTAINING_NUCLEOSOMES_AT_THE_CENTROMERE**	56	0.7789	1.7931	0.0174	0.0367
		67	24	76	95
**LIANG_HEMATOPOIESIS_STEM_CELL_NUMBER_SMALL_VS_HUGE_UP**	37	0.5331	1.7925	0.0019	0.0368
		82	43	84	07
**LUI_THYROID_CANCER_PAX8_PPARG_DN**	45	0.6212	1.7910	0.0157	0.0368
		85	65	79	2
**PUJANA_BRCA2_PCC_NETWORK**	400	0.5160	1.7927	0.0217	0.0368
		99	46	39	33
**KEGG_MISMATCH_REPAIR**	23	0.6799	1.7904	0.0098	0.0369
		82	84	23	13
**ELVIDGE_HIF1A_AND_HIF2A_TARGETS_UP**	38	0.5469	1.7914	0.0061	0.0369

		3	05	35	29
**SPIELMAN_LYMPHOBLAST_EUROPEAN_VS_ASIAN_UP**	466	0.4633	1.7916	0.0228	0.0369
		84	53	69	32
**YAMASHITA_LIVER_CANCER_WITH_EPCAM_UP**	52	0.5948	1.7888	0.0201	0.0373
		54	11	21	19
**PID_FOXM1_PATHWAY**	39	0.5569	1.7889	0.0175	0.0373
		51	25	1	88
**WANG_SMARCE1_TARGETS_DN**	346	0.4160	1.7883	<0.000	0.0374
		7	66	1	02
**BURTON_ADIPOGENESIS_PEAK_AT_16HR**	40	0.6212	1.7871	0.0337	0.0375
		59	73	97	94
**REACTOME_INFLUENZA_LIFE_CYCLE**	134	0.6405	1.7873	0.0225	0.0376
		4	56	41	25
**REACTOME_MEMBRANE_TRAFFICKING**	121	0.4476	1.7868	0.0020	0.0376
		86	18	66	46
**LANG_MYB_FAMILY_TARGETS**	29	0.5388	1.7863	0.0038	0.0377
		95	76	76	18
**REACTOME_SIGNALING_BY_THE_B_CELL_RECEPTOR_BCR**	121	0.4390	1.7849	0.0102	0.0381
		85	33	88	85
**REACTOME_RNA_POL_II_TRANSCRIPTION**	93	0.5399	1.7834	0.0161	0.0385
		65	33	94	41
**SCHMIDT_POR_TARGETS_IN_LIMB_BUD_UP**	25	0.7186	1.7836	0.0039	0.0385
		63	61	76	82
**MODY_HIPPOCAMPUS_PRENATAL**	42	0.6138	1.7826	0.0188	0.0387
		04	23	68	07
**SCIAN_CELL_CYCLE_TARGETS_OF_TP53_AND_TP73_DN**	22	0.7500	1.7823	0.01	0.0387
		21	5		25
**GOLDRATH_ANTIGEN_RESPONSE**	331	0.4734	1.7798	0.0183	0.0396
		26	62	67	17
**ZHAN_MULTIPLE_MYELOMA_SUBGROUPS**	30	0.6507	1.7790	0.0121	0.0398
		27	29	95	74
**XU_RESPONSE_TO_TRETINOIN_AND_NSC682994_DN**	15	0.7328	1.7774	0.0139	0.0402

		36	27	44	94
**REACTOME_DARPP_32_EVENTS**	23	0.5979	1.7768	0.0140	0.0404
		68	38	28	34
**FERREIRA_EWINGS_SARCOMA_UNSTABLE_VS_STABLE_UP**	148	0.5453	1.7759	0.0189	0.0406
		56	49	47	65
**ISHIDA_E2F_TARGETS**	50	0.7448	1.7742	0.0200	0.0410
		86	69	4	15
**KEGG_UBIQUITIN_MEDIATED_PROTEOLYSIS**	129	0.4303	1.7746	0.0079	0.0410
		51	86	37	62
**NUNODA_RESPONSE_TO_DASATINIB_IMATINIB_UP**	29	0.5490	1.7743	0.0062	0.0410
		41	97	76	82
**KONG_E2F3_TARGETS**	94	0.6471	1.7709	0.0295	0.0420
		21	7	86	81
**DELPUECH_FOXO3_TARGETS_DN**	39	0.6093	1.7706	0.0061	0.0421
		06	8	22	11
**BARRIER_COLON_CANCER_RECURRENCE_UP**	41	0.5888	1.7710	0.0059	0.0421
		64	09	17	66
**JIANG_HYPOXIA_CANCER**	76	0.4561	1.7699	0.0040	0.0422
		47	85	82	5
**SESTO_RESPONSE_TO_UV_C3**	19	0.6411	1.7693	0.0121	0.0424
		83	04	21	44
**STEIN_ESRRA_TARGETS_RESPONSIVE_TO_ESTROGEN_DN**	38	0.6351	1.7680	0.0193	0.0425
		26	83	8	94
**ABRAHAM_ALPC_VS_MULTIPLE_MYELOMA_DN**	19	0.5675	1.7681	0.0102	0.0426
		24	45	04	76
**KEGG_PROTEIN_EXPORT**	22	0.7161	1.7682	0.0080	0.0427
		37	02	81	63
**IVANOVA_HEMATOPOIESIS_LATE_PROGENITOR**	497	0.4109	1.7644	0.0019	0.0437
		14	1	84	7
**LANDIS_ERBB2_BREAST_TUMORS_65_UP**	21	0.6193	1.7649	0.0173	0.0437
		38	71	08	78
**KEGG_SPLICEOSOME**	123	0.5487	1.7645	0.0229	0.0438

		62	03	17	4
**CHENG_RESPONSE_TO_NICKEL_ACETATE**	41	0.4876	1.7617	0.0020	0.0448
		03	16	33	07
**EPPERT_PROGENITOR**	125	0.5359	1.7604	0.0097	0.0451
		36	8	66	42
**HORTON_SREBF_TARGETS**	24	0.7603	1.7592	0.0083	0.0455
		23	95	86	46
**REACTOME_CTNNB1_PHOSPHORYLATION_CASCADE**	15	0.6303	1.7586	0.0077	0.0456
		18	69	52	33
**JIANG_AGING_CEREBRAL_CORTEX_UP**	34	0.5458	1.7579	0.0102	0.0457
		77	79	04	71
**KRIEG_KDM3A_TARGETS_NOT_HYPOXIA**	185	0.4316	1.7575	0.0019	0.0458
		31	4	57	39
**REACTOME_G2_M_CHECKPOINTS**	41	0.7056	1.7566	0.0160	0.0460
		8	99	32	72
**FRASOR_RESPONSE_TO_SERM_OR_FULVESTRANT_DN**	50	0.6403	1.7536	0.0314	0.0464
		3	94	34	63
**CHESLER_BRAIN_QTL_CIS**	69	0.4306	1.7541	<0.000	0.0465
		5	47	1	19
**PID_AURORA_B_PATHWAY**	37	0.6212	1.7546	0.0297	0.0465
		51	62	03	51
**REACTOME_DESTABILIZATION_OF_MRNA_BY_TRISTETRAPROLIN_TTP**	17	0.6340	1.7543	0.0039	0.0465
		52	04	45	65
**KEGG_PYRUVATE_METABOLISM**	37	0.5641	1.7537	0.0160	0.0465
		3	04	32	71
**BIOCARTA_IGF1MTOR_PATHWAY**	20	0.5481	1.7551	0.0099	0.0465
		56	43	21	94
**REACTOME_FATTY_ACYL_COA_BIOSYNTHESIS**	18	0.6272	1.7530	0.0079	0.0466
		04	88	84	16
**PENG_GLUCOSE_DEPRIVATION_DN**	162	0.4067	1.7547	<0.000	0.0466
		1	85	1	25
**BURTON_ADIPOGENESIS_6**	177	0.4644	1.7517	0.0124	0.0470

		83	77	48	59
**ACOSTA_PROLIFERATION_INDEPENDENT_MYC_TARGETS_UP**	75	0.4951	1.7500	0.0101	0.0475
		18	91	21	89
**BARRIER_CANCER_RELAPSE_TUMOR_SAMPLE_UP**	15	0.7834	1.7488	0.0060	0.0479
		28	84	24	62
**REACTOME_POST_TRANSLATIONAL_PROTEIN_MODIFICATION**	174	0.4229	1.7477	0.0056	0.0480
		53	77	93	87
**LUI_TARGETS_OF_PAX8_PPARG_FUSION**	34	0.6587	1.7479	0.0239	0.0481
		7	45	52	38
**TIEN_INTESTINE_PROBIOTICS_6HR_UP**	55	0.5906	1.7479	0.0263	0.0482
		24	93	16	31
**MAYBURD_RESPONSE_TO_L663536_DN**	51	0.5718	1.7469	0.0193	0.0482
		52	31	8	91
**ACEVEDO_LIVER_CANCER_WITH_H3K9ME3_DN**	107	0.5278	1.7451	0.0195	0.0488
		12	83	69	2
**GROSS_HYPOXIA_VIA_HIF1A_UP**	75	0.4719	1.7453	0.0082	0.0488
		67	89	3	55
**JOHANSSON_GLIOMAGENESIS_BY_PDGFB_UP**	57	0.4748	1.7435	0.0213	0.0493
		83	53	59	52
**WANG_CISPLATIN_RESPONSE_AND_XPC_UP**	192	0.4124	1.7428	0.0063	0.0495
		91	74	03	31
**XU_HGF_SIGNALING_NOT_VIA_AKT1_48HR_DN**	20	0.5953	1.7417	0.0134	0.0498
		06	71	87	92
**WHITFIELD_CELL_CYCLE_G2**	169	0.4466	1.7401	0.0238	0.0504
		57	57	57	64
**WALLACE_PROSTATE_CANCER_UP**	20	0.6375	1.7395	0.0077	0.0506
		15	36	37	25
**JI_RESPONSE_TO_FSH_UP**	70	0.4358	1.7361	0.0081	0.0519
		5	77	47	72
**REACTOME_ACTIVATION_OF_ATR_IN_RESPONSE_TO_REPLICATION_STRESS**	35	0.7034	1.7357	0.0220	0.0520
		37	83	44	35
**JAIN_NFKB_SIGNALING**	71	0.4490	1.7353	0.0041	0.0521

		12	16	75	22
**KEGG_CYSTEINE_AND_METHIONINE_METABOLISM**	33	0.5031	1.7349	0.0038	0.0521
		23	56	46	71
**REACTOME_DNA_STRAND_ELONGATION**	30	0.7435	1.734	0.0184	0.0524
		94		43	74
**CHEMNITZ_RESPONSE_TO_PROSTAGLANDIN_E2_UP**	129	0.4915	1.7325	0.0234	0.0530
		91	73	54	35
**PUJANA_BREAST_CANCER_LIT_INT_NETWORK**	97	0.4939	1.7312	0.0352	0.0533
		93	87	25	66
**FLECHNER_PBL_KIDNEY_TRANSPLANT_REJECTED_VS_OK_UP**	62	0.4589	1.7314	0.0096	0.0534
		38	64	9	14
**BIOCARTA_CDC42RAC_PATHWAY**	16	0.6935	1.7308	0.0117	0.0534
		99	44	42	24
**BIOCARTA_EIF_PATHWAY**	16	0.6211	1.7292	0.0118	0.0538
		1	68	58	87
**SANSOM_APC_TARGETS_UP**	114	0.4115	1.7290	<0.000	0.0539
		77	11	1	
**HASLINGER_B_CLL_WITH_6Q21_DELETION**	18	0.6906	1.7293	0.0173	0.0539
		66	59	41	59
**LOCKWOOD_AMPLIFIED_IN_LUNG_CANCER**	205	0.4389	1.7284	0.0180	0.0540
		73	84	36	44
**VANHARANTA_UTERINE_FIBROID_WITH_7Q_DELETION_UP**	65	0.4699	1.7263	0.0121	0.0549
		02	59	7	87
**ZHOU_TNF_SIGNALING_30MIN**	51	0.5542	1.7254	0.0227	0.0552
		09	51	27	36
**DARWICHE_SQUAMOUS_CELL_CARCINOMA_UP**	129	0.3974	1.7242	0.0020	0.0554
		72	04	45	86
**PEART_HDAC_PROLIFERATION_CLUSTER_UP**	55	0.4745	1.7244	0.0135	0.0555
		59		66	14
**REACTOME_MRNA_CAPPING**	28	0.5638	1.7245	0.0261	0.0555
		57	67	04	35
**REACTOME_MRNA_PROCESSING**	151	0.5225	1.7231	0.0286	0.0556

		35		3	69
**PIONTEK_PKD1_TARGETS_DN**	16	0.6092	1.7234	0.01	0.0557
		58	55		07
**REACTOME_GOLGI_ASSOCIATED_VESICLE_BIOGENESIS**	50	0.4729	1.7231	0.0081	0.0557
		36	1	8	82
**SESTO_RESPONSE_TO_UV_C1**	72	0.5064	1.7220	0.0136	0.0559
		38	05	72	41
**MACLACHLAN_BRCA1_TARGETS_UP**	21	0.5895	1.7221	0.0177	0.0560
		3	26	87	1
**REACTOME_ACTIVATION_OF_THE_MRNA_UPON_BINDING_OF_THE_CAP_BINDING_COMPLEX_AND_EIFS_AND_SUBSEQUENT_BIN**	56	0.6636	1.7213	0.0335	0.0560
**DING_TO_43S**		24	77	97	91
**REACTOME_FORMATION_OF_THE_TERNARY_COMPLEX_AND_SUBSEQUENTLY_THE_43S_COMPLEX**	48	0.7044	1.7207	0.0278	0.0560
		28	67	88	92
**LI_WILMS_TUMOR_VS_FETAL_KIDNEY_1_DN**	159	0.5303	1.7209	0.0307	0.0561
		36	14	38	58
**REACTOME_E2F_MEDIATED_REGULATION_OF_DNA_REPLICATION**	33	0.6500	1.7189	0.0217	0.0568
		71	46	39	5
**BURTON_ADIPOGENESIS_10**	27	0.5704	1.7182	0.0196	0.0570
		59	49	85	35
**MORI_PLASMA_CELL_UP**	49	0.5514	1.7170	0.0118	0.0573
		16	84	11	54
**ZHU_CMV_ALL_UP**	118	0.4839	1.7171	0.0260	0.0574
		68	52	52	36
**ENK_UV_RESPONSE_KERATINOCYTE_DN**	479	0.4348	1.7162	0.0136	0.0576
		47	48	99	05
**PUIFFE_INVASION_INHIBITED_BY_ASCITES_UP**	81	0.4359	1.7152	0.0098	0.0578
		46	08	81	22
**MILI_PSEUDOPODIA_HAPTOTAXIS_UP**	483	0.5632	1.7153	0.0352	0.0578
		22	86	94	74
**IIZUKA_LIVER_CANCER_PROGRESSION_G1_G2_DN**	24	0.5636	1.7142	0.0098	0.0580
		7	55	23	78
**REACTOME_SRP_DEPENDENT_COTRANSLATIONAL_PROTEIN_TARGETING_TO_MEMBRANE**	107	0.6729	1.7133	0.0341	0.0581

		74	63	37	06
**PARK_HSC_VS_MULTIPOTENT_PROGENITORS_DN**	18	0.6096	1.7143	0.0116	0.0581
		16	58	73	47
**ZHONG_SECRETOME_OF_LUNG_CANCER_AND_FIBROBLAST**	127	0.4679	1.7133	0.0161	0.0582
		71	96	29	21
**REACTOME_LAGGING_STRAND_SYNTHESIS**	19	0.7440	1.7135	0.0061	0.0582
		25	34	35	65
**COLLIS_PRKDC_REGULATORS**	15	0.5845	1.7120	0.0158	0.0586
		02	33	73	08
**LY_AGING_PREMATURE_DN**	29	0.6169	1.7101	0.0225	0.0593
		12	99	87	13
**GAVIN_FOXP3_TARGETS_CLUSTER_T7**	95	0.4574	1.7082	0.0285	0.0599
		87	95	71	84
**NAM_FXYD5_TARGETS_DN**	18	0.6297	1.7083	0.0304	0.0600
		72	51	88	86
**KEGG_VALINE_LEUCINE_AND_ISOLEUCINE_DEGRADATION**	44	0.5816	1.7077	0.0203	0.0601
		83	07	67	14
**FINETTI_BREAST_CANCER_KINOME_RED**	16	0.8483	1.7065	0.0081	0.0605
		43	43	3	57
**STEIN_ESR1_TARGETS**	81	0.4541	1.7042	0.0154	0.0616
		22	17	74	23
**REACTOME_PROCESSING_OF_CAPPED_INTRON_CONTAINING_PRE_MRNA**	132	0.5387	1.7028	0.0269	0.0620
		69	69	15	01
**WANG_LMO4_TARGETS_DN**	326	0.4093	1.7025	0.0058	0.0620
		53	43	37	44
**REACTOME_TRANS_GOLGI_NETWORK_VESICLE_BUDDING**	57	0.4581	1.7029	0.0121	0.0620
		16	59	95	92
**VERNELL_RETINOBLASTOMA_PATHWAY_UP**	68	0.6132	1.7022	0.0463	0.0621
		06	04	71	02
**AIYAR_COBRA1_TARGETS_DN**	28	0.5074	1.6994	0.0102	0.0634
		03	32	04	13
**REACTOME_PEROXISOMAL_LIPID_METABOLISM**	20	0.6190	1.6984	0.0294	0.0636

		22	04	74	91
**WANG_TUMOR_INVASIVENESS_DN**	201	0.3849	1.6986	0.0019	0.0637
		45	17	12	04
**REACTOME_FORMATION_OF_RNA_POL_II_ELONGATION_COMPLEX_**	35	0.5369	1.6979	0.0348	0.0637
		1	54	36	41
**LIU_PROSTATE_CANCER_UP**	88	0.4326	1.6969	0.0159	0.0639
		77	31	36	89
**ELVIDGE_HYPOXIA_BY_DMOG_DN**	54	0.4801	1.6971	0.0224	0.0640
		05	26	03	53
**BROWN_MYELOID_CELL_DEVELOPMENT_DN**	119	0.4065	1.6946	0.0080	0.0647
		61	5	48	78
**ZHONG_RESPONSE_TO_AZACITIDINE_AND_TSA_DN**	63	0.5139	1.6947	0.0240	0.0648
		17	47	48	49
**JIANG_AGING_CEREBRAL_CORTEX_DN**	52	0.4589	1.6947	0.0132	0.0649
		54	53	83	78
**PID_PRL_SIGNALING_EVENTS_PATHWAY**	23	0.5449	1.6909	0.0203	0.0663
		08	06	25	12
**PID_ERBB1_DOWNSTREAM_PATHWAY**	105	0.4237	1.6905	0.008	0.0663
		54	63		62
**REACTOME_ENDOSOMAL_SORTING_COMPLEX_REQUIRED_FOR_TRANSPORT_ESCRT**	26	0.5948	1.6910	0.0204	0.0663
		47	6	92	86
**BOHN_PRIMARY_IMMUNODEFICIENCY_SYNDROM_UP**	45	0.5043	1.6895	0.0173	0.0666
		31	51	41	65
**REACTOME_UNFOLDED_PROTEIN_RESPONSE**	72	0.4445	1.6891	0.0216	0.0667
		26	31	11	26
**KEGG_STEROID_BIOSYNTHESIS**	16	0.7009	1.6879	0.0179	0.0671
		97	76	64	9
**KEGG_PEROXISOME**	77	0.4954	1.6867	0.0290	0.0677
		96	25	46	64
**VANTVEER_BREAST_CANCER_ESR1_DN**	225	0.4134	1.6855	0.0117	0.0682
			76	88	25
**JEON_SMAD6_TARGETS_DN**	19	0.6084	1.6851	0.0325	0.0683

		16	04	2	52
**KEGG_CITRATE_CYCLE_TCA_CYCLE**	29	0.6377	1.6839	0.0301	0.0685
		57	89	2	47
**REACTOME_MRNA_SPLICING**	104	0.5445	1.6840	0.0390	0.0686
		84	74	14	35
**TURASHVILI_BREAST_DUCTAL_CARCINOMA_VS_LOBULAR_NORMAL_UP**	71	0.5362	1.6841	0.0218	0.0687
		14	06	69	45
**TOOKER_GEMCITABINE_RESISTANCE_UP**	76	0.4705	1.6829	0.0245	0.0689
		23	3	4	76
**KAUFFMANN_DNA_REPAIR_GENES**	219	0.4670	1.6816	0.0349	0.0692
		89	27	79	96
**BIOCARTA_G2_PATHWAY**	24	0.5847	1.6816	0.0251	0.0694
		62	46	57	2
**REACTOME_LYSOSOME_VESICLE_BIOGENESIS**	23	0.5483	1.6783	0.0333	0.0707
		69	64	33	3
**HAHTOLA_CTCL_CUTANEOUS**	25	0.5500	1.6772	0.0214	0.0712
		37	66	84	59
**NAKAYAMA_SOFT_TISSUE_TUMORS_PCA2_UP**	85	0.4870	1.6753	0.0260	0.0721
		53	88	52	43
**REICHERT_MITOSIS_LIN9_TARGETS**	27	0.6842	1.6749	0.03	0.0722
		71	95		39
**KEGG_ARGININE_AND_PROLINE_METABOLISM**	50	0.4780	1.6728	0.0161	0.0727
		13	49	94	49
**CHOI_ATL_CHRONIC_VS_ACUTE_DN**	18	0.6489	1.6732	0.0193	0.0727
		09	58	05	74
**DING_LUNG_CANCER_EXPRESSION_BY_COPY_NUMBER**	96	0.5393	1.6722	0.0415	0.0728
		12	26	88	02
**REACTOME_INTRINSIC_PATHWAY_FOR_APOPTOSIS**	29	0.5173	1.6716	0.0127	0.0728
		15	7	66	44
**KEGG_RNA_POLYMERASE**	29	0.5933	1.6733	0.0309	0.0728
		45	18	28	69
**FOURNIER_ACINAR_DEVELOPMENT_LATE_DN**	21	0.6241	1.6723	0.0391	0.0728

		73	3	39	8
**WANG_RESPONSE_TO_FORSKOLIN_UP**	22	0.581	1.6734	0.0292	0.0729
			66	4	42
**CEBALLOS_TARGETS_OF_TP53_AND_MYC_UP**	21	0.5235	1.6717	0.0254	0.0729
		16	32	4	42
**SESTO_RESPONSE_TO_UV_C6**	38	0.5233	1.6710	0.0223	0.0731
		31	14	12	21
**BURTON_ADIPOGENESIS_4**	44	0.4866	1.6692	0.0084	0.0740
		04	2	39	63
**WILLIAMS_ESR1_TARGETS_UP**	26	0.5278	1.6680	0.0160	0.0744
		03	23	97	97
**REACTOME_FORMATION_OF_THE_HIV1_EARLY_ELONGATION_COMPLEX**	28	0.5575	1.6682	0.0334	0.0745
		71	55	03	02
**REACTOME_EXTENSION_OF_TELOMERES**	27	0.6812	1.6664	0.0306	0.0752
		76	51	12	59
**KEGG_PROPANOATE_METABOLISM**	32	0.5545	1.6659	0.0363	0.0754
		64	4	64	13
**BIOCARTA_MITOCHONDRIA_PATHWAY**	21	0.5852	1.6652	0.0228	0.0757
		1	39	22	
**TAKAO_RESPONSE_TO_UVB_RADIATION_UP**	82	0.5627	1.6644	0.0425	0.0759
		38	5	53	76
**PUIFFE_INVASION_INHIBITED_BY_ASCITES_DN**	141	0.4601	1.6632	0.0234	0.0765
		36	58	83	11
**STEIN_ESRRA_TARGETS**	495	0.3681	1.6628	0.0224	0.0765
		32	04	49	14
**TOMIDA_METASTASIS_UP**	26	0.5558	1.6620	0.0254	0.0766
		16	16	9	91
**KAPOSI_LIVER_CANCER_MET_UP**	17	0.5654	1.6598	0.0118	0.0777
		65	55	34	7
**PID_BARD1_PATHWAY**	28	0.6276	1.6591	0.0463	0.0780
		41	55	71	97
**REACTOME_GLOBAL_GENOMIC_NER_GG_NER**	32	0.5662	1.6581	0.0383	0.0784

		59	07	84	03
REACTOME_INTERACTIONS_OF_VPR_WITH_HOST_CELLULAR_PROTEINS	31	0.5841	1.6567	0.026	0.0790
		03	43		38
LUI_THYROID_CANCER_CLUSTER_3	28	0.6713	1.6558	0.0364	0.0794
		69	67	37	36
REACTOME_KINESINS	23	0.5824	1.6537	0.0464	0.0804
		57	89	65	43
REACTOME_PROCESSIVE_SYNTHESIS_ON_THE_LAGGING_STRAND	15	0.7154	1.6526	0.0184	0.0806
		98	84	05	37
GRAHAM_CML_QUIESCENT_VS_NORMAL_QUIESCENT_UP	80	0.4682	1.6528	0.0304	0.0806
		66	95	26	68
PECE_MAMMARY_STEM_CELL_UP	134	0.5207	1.6520	0.0440	0.0807
		72	41	25	3
BIOCARTA_CELLCYCLE_PATHWAY	23	0.5644	1.6520	0.0256	0.0808
		4	92	41	48
LY_AGING_MIDDLE_DN	16	0.7561	1.6501	0.0258	0.0815
			95	45	29
REACTOME_DEADENYLATION_OF_MRNA	17	0.5705	1.6484	0.0253	0.0821
		13	77	41	68
XU_CREBBP_TARGETS_UP	24	0.5177	1.6485	0.0202	0.0823
		31	99	84	71
MCCABE_HOXC6_TARGETS_CANCER_DN	20	0.5373	1.6472	0.0186	0.0827
		91	87	34	83
REACTOME_G0_AND_EARLY_G1	23	0.6355	1.6463	0.0337	0.0830
		77	25	3	86
CHAUHAN_RESPONSE_TO_METHOXYESTRADIOL_DN	97	0.4402	1.6465	0.0436	0.0831
		67	32	51	03
REACTOME_POST_TRANSLATIONAL_MODIFICATION_SYNTHESIS_OF_GPI_ANCHORED_PROTEINS	26	0.5357	1.6453	0.0281	0.0833
		51	59	69	6
MARIADASON_RESPONSE_TO_CURCUMIN_SULINDAC_7	16	0.5352	1.6454	0.0210	0.0834
		15	98	33	24
REACTOME_DESTABILIZATION_OF_MRNA_BY_KSRP	17	0.6055	1.6424	0.0162	0.0848

		01	14	27	27
**WATANABE_RECTAL_CANCER_RADIOTHERAPY_RESPONSIVE_DN**	89	0.4373	1.6414	0.0142	0.0853
		04	22	28	07
**NOUZOVA_TRETINOIN_AND_H4_ACETYLATION**	130	0.4091	1.6410	0.0103	0.0853
		43	74	31	46
**YIH_RESPONSE_TO_ARSENITE_C3**	35	0.4778	1.6403	0.0317	0.0856
		16	92	46	17
**SU_TESTIS**	63	0.5214	1.6394	0.0299	0.0860
		48	94	4	01
**GAZDA_DIAMOND_BLACKFAN_ANEMIA_MYELOID_DN**	36	0.4722	1.6372	0.0317	0.0868
		36	08	46	05
**MARTORIATI_MDM4_TARGETS_FETAL_LIVER_UP**	207	0.3562	1.6372	0.0020	0.0869
		76	58	49	32
**KEGG_ALANINE_ASPARTATE_AND_GLUTAMATE_METABOLISM**	32	0.4793	1.6367	0.0163	0.0869
		99	68	6	56
**BIOCARTA_NDKDYNAMIN_PATHWAY**	18	0.5215	1.6356	0.0337	0.0875
		93	37	08	13
**FLECHNER_PBL_KIDNEY_TRANSPLANT_OK_VS_DONOR_UP**	145	0.3668	1.6333	0.0122	0.0886
		37	24	2	85
**PID_LIS1_PATHWAY**	28	0.4942	1.6334	0.0136	0.0887
		05	29	72	64
**REACTOME_ANTIVIRAL_MECHANISM_BY_IFN_STIMULATED_GENES**	64	0.4801	1.6309	0.0487	0.0899
		43	54	33	68
**SCHAEFFER_PROSTATE_DEVELOPMENT_AND_CANCER_BOX4_DN**	32	0.5735	1.6300	0.0433	0.0904
		94	71	93	09
**GALLUZZI_PERMEABILIZE_MITOCHONDRIA**	43	0.4467	1.6291	0.0187	0.0908
		19	59	11	11
**REACTOME_RESOLUTION_OF_AP_SITES_VIA_THE_MULTIPLE_NUCLEOTIDE_PATCH_REPLACEMENT_PATHWAY**	17	0.648	1.6287	0.0460	0.0909
			42	25	24
**PID_ATM_PATHWAY**	33	0.5301	1.6274	0.0344	0.0912
		27	88	83	66
**GEORGES_CELL_CYCLE_MIR192_TARGETS**	61	0.5311	1.6276	0.0373	0.0912

		2	83	28	86
**REACTOME_PYRUVATE_METABOLISM**	18	0.5885	1.6277	0.0327	0.0914
		87	29	2	07
**KOINUMA_COLON_CANCER_MSI_UP**	16	0.6697	1.6265	0.0447	0.0917
		29	8	47	67
**KAAB_HEART_ATRIUM_VS_VENTRICLE_DN**	254	0.3630	1.6230	0.0103	0.0938
		84	92	31	12
**FARMER_BREAST_CANCER_CLUSTER_3**	15	0.5907	1.6223	0.0421	0.0941
		07	74	25	41
**NADLER_OBESITY_DN**	47	0.4687	1.6212	0.0319	0.0945
		76	92	36	27
**MODY_HIPPOCAMPUS_NEONATAL**	35	0.4977	1.6208	0.0272	0.0946
		75	05	37	9
**LIU_SOX4_TARGETS_DN**	292	0.3467	1.6146	0.0159	0.0982
		85	78	05	92
**KEGG_N_GLYCAN_BIOSYNTHESIS**	46	0.4704	1.6142	0.0257	0.0983
		2	37	43	82
**REACTOME_CITRIC_ACID_CYCLE_TCA_CYCLE**	19	0.6690	1.6131	0.0482	0.0989
		74	29	9	5
**STEIN_ESRRA_TARGETS_UP**	361	0.3995	1.6104	0.0452	0.1005
		81	5	67	52
**MOOTHA_TCA**	16	0.6943	1.6091	0.0405	0.1011
		64	54	68	31
**REACTOME_AMINO_ACID_SYNTHESIS_AND_INTERCONVERSION_TRANSAMINATION**	15	0.6368	1.6086	0.0419	0.1012
		98	64	16	79
**REACTOME_DESTABILIZATION_OF_MRNA_BY_BRF1**	17	0.5725	1.6079	0.0321	0.1013
		3	64	29	84
**PID_ARF6_DOWNSTREAM_PATHWAY**	15	0.5822	1.6074	0.0294	0.1015
		71	56	7	12
**LAU_APOPTOSIS_CDKN2A_UP**	55	0.4169	1.6079	0.0244	0.1015
		65	96	4	4
**HOUSTIS_ROS**	35	0.4920	1.6033	0.0400	0.1040

		33	61	76	48
**TARTE_PLASMA_CELL_VS_B_LYMPHOCYTE_UP**	75	0.4751	1.6026	0.0468	0.1043
		4	71	75	77
**HOEBEKE_LYMPHOID_STEM_CELL_DN**	82	0.4079	1.6014	0.0341	0.1050
		43	59	37	61
**ZHENG_GLIOBLASTOMA_PLASTICITY_UP**	239	0.3839	1.6004	0.0387	0.1054
		12		6	14
**MARKEY_RB1_CHRONIC_LOF_UP**	111	0.3793	1.6004	0.0190	0.1055
		09	13	48	83
**PID_TELOMERASE_PATHWAY**	67	0.4088	1.5984	0.0191	0.1064
		49	28	2	56
**ANDERSEN_LIVER_CANCER_KRT19_UP**	34	0.6071	1.5980	0.0470	0.1065
		61	41	35	54
**ZHONG_SECRETOME_OF_LUNG_CANCER_AND_MACROPHAGE**	74	0.4571	1.5969	0.0488	0.1070
		85	75	8	38
**KIM_WT1_TARGETS_12HR_DN**	200	0.3632	1.5941	0.0077	0.1086
		62	39	82	84
**PARK_HSC_VS_MULTIPOTENT_PROGENITORS_UP**	18	0.5177	1.5926	0.0234	0.1095
		19	99	38	33
**SWEET_LUNG_CANCER_KRAS_UP**	465	0.3502	1.5919	0.0162	0.1099
		69	51	27	68
**DUTERTRE_ESTRADIOL_RESPONSE_6HR_UP**	218	0.3827	1.59	0.0298	0.1111
		34		21	64
**KEGG_AMINO_SUGAR_AND_NUCLEOTIDE_SUGAR_METABOLISM**	43	0.4896	1.5858	0.0425	0.1137
		54	26	96	01
**JIANG_VHL_TARGETS**	129	0.3853	1.5841	0.0312	0.1145
		48	67	5	31
**BYSTRYKH_HEMATOPOIESIS_STEM_CELL_QTL_CIS**	109	0.3632	1.5828	0.0161	0.1149
		18	57	94	65
**TAYLOR_METHYLATED_IN_ACUTE_LYMPHOBLASTIC_LEUKEMIA**	73	0.4252	1.5822	0.0099	0.1152
		58	85	8	42
**SWEET_KRAS_ONCOGENIC_SIGNATURE**	88	0.4108	1.5809	0.0342	0.1154

		03	63	74	57
**KARAKAS_TGFB1_SIGNALING**	18	0.5782	1.5800	0.0483	0.1155
		01	02	56	67
**PID_P53_REGULATION_PATHWAY**	57	0.4305	1.5790	0.0260	0.1159
		18	63	52	44
**DARWICHE_PAPILLOMA_RISK_HIGH_UP**	131	0.3694	1.5772	0.0123	0.1170
		32	7	71	92
**KYNG_RESPONSE_TO_H2O2_VIA_ERCC6_UP**	39	0.4208	1.5764	0.0160	0.1175
		78	67	32	28
**MARIADASON_RESPONSE_TO_BUTYRATE_SULINDAC_4**	21	0.5582	1.5732	0.0328	0.1191
			34	54	6
**APRELIKOVA_BRCA1_TARGETS**	48	0.4544	1.5736	0.0355	0.1192
		59	02	73	71
**ONDER_CDH1_TARGETS_1_DN**	163	0.4480	1.5718	0.0406	0.1199
		28	43	19	6
**HOFMANN_MYELODYSPLASTIC_SYNDROM_LOW_RISK_DN**	30	0.5303	1.5707	0.0443	0.1203
		82	44	55	72
**ACEVEDO_NORMAL_TISSUE_ADJACENT_TO_LIVER_TUMOR_DN**	336	0.3906	1.5688	0.0481	0.1214
		86	42	93	85
**JAEGER_METASTASIS_UP**	43	0.4618	1.5682	0.0444	0.1215
		16	32	02	64
**KYNG_WERNER_SYNDROM_AND_NORMAL_AGING_DN**	210	0.3187	1.5679	0.0058	0.1215
		31	57	82	77
**ST_FAS_SIGNALING_PATHWAY**	63	0.4115	1.5664	0.0235	0.1222
		46	69	76	91
**COLINA_TARGETS_OF_4EBP1_AND_4EBP2**	346	0.3394	1.5650	0.0079	0.1227
		31	24	21	94
**FARMER_BREAST_CANCER_APOCRINE_VS_BASAL**	313	0.3389	1.5614	0.0190	0.1252
		23	28	48	37
**STEIN_ESRRA_TARGETS_RESPONSIVE_TO_ESTROGEN_UP**	29	0.4655	1.5593	0.0345	0.1267
		3	49	53	19
**YAO_TEMPORAL_RESPONSE_TO_PROGESTERONE_CLUSTER_9**	70	0.4232	1.5554	0.0364	0.1291

		18	88	37	51
**PID_MYC_PATHWAY**	25	0.4963	1.5545	0.0400	0.1297
		31	05	8	79
**DARWICHE_PAPILLOMA_RISK_LOW_UP**	143	0.3534	1.5511	0.0140	0.1317
		92	85	85	87
**LI_CYTIDINE_ANALOG_PATHWAY**	15	0.5370	1.5500	0.0453	0.1324
		01	85	65	65
**GENTILE_UV_RESPONSE_CLUSTER_D5**	37	0.4398	1.5493	0.0273	0.1328
		85	88	97	24
**KEGG_BASAL_TRANSCRIPTION_FACTORS**	34	0.4824	1.5464	0.0464	0.1347
		17	6	22	57
**WANG_LMO4_TARGETS_UP**	329	0.3331	1.5446	0.0229	0.1356
		54	99	89	16
**TERAO_AOX4_TARGETS_SKIN_UP**	35	0.4433	1.5439	0.0334	0.1357
		71	84	65	74
**HAHTOLA_MYCOSIS_FUNGOIDES_SKIN_UP**	173	0.3647	1.5413	0.0346	0.1375
		35	52	23	01
**SHEPARD_BMYB_MORPHOLINO_UP**	200	0.3191	1.5386	0.0061	0.1387
		2	35	98	96
**PID_PI3KCI_AKT_PATHWAY**	35	0.4279	1.5369	0.0452	0.1398
		32	1	76	79
**BIOCARTA_RHO_PATHWAY**	32	0.4712	1.5364	0.0465	0.1401
		62	19	59	02
**HOLLMANN_APOPTOSIS_VIA_CD40_DN**	239	0.3423	1.5357	0.0421	0.1404
		74	7	69	66
**SWEET_KRAS_TARGETS_DN**	59	0.4081	1.5335	0.0314	0.1416
		47	73	96	2
**GRYDER_PAX3FOXO1_TOP_ENHANCERS**	427	0.3247	1.5309	0.0230	0.1434
		65	07	33	34
**ZHAN_MULTIPLE_MYELOMA_CD2_DN**	45	0.4181	1.5304	0.0311	0.1434
		27	32	2	56
**SCHAEFFER_PROSTATE_DEVELOPMENT_6HR_DN**	482	0.3183	1.5306	0.0196	0.1434

		92	27	85	78
**GROSS_HYPOXIA_VIA_ELK3_AND_HIF1A_DN**	100	0.3573	1.5289	0.0242	0.1442
		33	66	42	36
**YAGI_AML_RELAPSE_PROGNOSIS**	34	0.4424	1.5259	0.0281	0.1459
		63	19	69	39
**LABBE_WNT3A_TARGETS_UP**	108	0.3648	1.5219	0.0294	0.1486
		43	78	12	85
**GUO_TARGETS_OF_IRS1_AND_IRS2**	92	0.3718	1.5186	0.0320	0.1503
		53	62	64	25
**DARWICHE_SKIN_TUMOR_PROMOTER_UP**	124	0.3694	1.5181	0.0289	0.1503
		68	83	26	35
**FARMER_BREAST_CANCER_BASAL_VS_LULMINAL**	317	0.3242	1.5182	0.0172	0.1505
		87	36	74	
**PID_FOXO_PATHWAY**	49	0.4129	1.5165	0.0469	0.1511
		02	95	92	48
**KEGG_P53_SIGNALING_PATHWAY**	66	0.4055	1.5156	0.0355	0.1517
		59	87	73	3
**SYED_ESTRADIOL_RESPONSE**	19	0.4569	1.5115	0.0419	0.1542
		58	09	85	65
**AIYAR_COBRA1_TARGETS_UP**	38	0.4124	1.5065	0.0399	0.1578
		97	05	24	18
**JIANG_HYPOXIA_NORMAL**	294	0.3291	1.5058	0.0273	0.1582
		16	82	97	01
**MORI_SMALL_PRE_BII_LYMPHOCYTE_DN**	74	0.3786	1.5031	0.0391	0.1598
		3	33	75	28
**WAKABAYASHI_ADIPOGENESIS_PPARG_RXRA_BOUND_WITH_H4K20ME1_MARK**	132	0.3572	1.5031	0.0440	0.1600
		83	88	88	09
**PID_CDC42_PATHWAY**	69	0.3884	1.4931	0.0484	0.1668
		99	35	5	96
**GENTILE_UV_RESPONSE_CLUSTER_D7**	36	0.4132	1.4905	0.0486	0.1683
		92	35	82	35
**ENK_UV_RESPONSE_EPIDERMIS_UP**	277	0.3508	1.4838	0.0482	0.1737

		83	93	9	34
**LINSLEY_MIR16_TARGETS**	190	0.3339	1.4822	0.0355	0.1744
		86	21	73	35
**LIU_COMMON_CANCER_GENES**	67	0.3662	1.4810	0.0300	0.1750
		12	51	6	94
**UEDA_PERIFERAL_CLOCK**	161	0.3244	1.4786	0.0321	0.1763
		41	95	29	57
**AMUNDSON_GENOTOXIC_SIGNATURE**	98	0.3470	1.4760	0.0333	0.1773
		22	36	99	71
**DITTMER_PTHLH_TARGETS_DN**	72	0.3547	1.4685	0.0418	0.1834
		18	04	25	84
**CREIGHTON_ENDOCRINE_THERAPY_RESISTANCE_4**	278	0.2898	1.4627	0.0039	0.1871
		04	83	6	38
**ZHAN_MULTIPLE_MYELOMA_UP**	60	0.3755	1.4620	0.0427	0.1873
		87	04	7	99
**SERVITJA_LIVER_HNF1A_TARGETS_UP**	132	0.3392	1.4593	0.0444	0.1885
		98	31	87	87
**WALLACE_PROSTATE_CANCER_RACE_DN**	74	0.3706	1.4595	0.0441	0.1888
		89	82	77	43
**REACTOME_DIABETES_PATHWAYS**	121	0.3408	1.4403	0.0366	0.2020
		34	72	8	25
**CREIGHTON_ENDOCRINE_THERAPY_RESISTANCE_1**	482	0.2972	1.4367	0.0319	0.2050
		32	18	36	6
**LIANG_HEMATOPOIESIS_STEM_CELL_NUMBER_LARGE_VS_TINY_DN**	40	0.3820	1.4351	0.0408	0.2061
		27	96	16	87
**BOYLAN_MULTIPLE_MYELOMA_C_D_UP**	129	0.3265	1.4231	0.0485	0.2150
		25	19	83	34
**KIM_WT1_TARGETS_8HR_DN**	114	0.3147	1.4213	0.016	0.2164
		63	5		34
**PURBEY_TARGETS_OF_CTBP1_AND_SATB1_DN**	172	0.3099	1.4065	0.0464	0.2272
		53	47	65	85
