# Supplementary material for: Clinical significance and biological mechanisms of glutathione S-transferase mu gene family in colon adenocarcinoma
Source: BMC Med Genet. 2020 Jun 15;21:130. doi: 10.1186/s12881-020-01066-2 (PMC7296959; doi:10.1186/s12881-020-01066-2)
Supplement: Supplementary file 6 — Additional file 6: Table S3. GO enrichment results by GSEA for GSTM2 (c5.all.v6.2.symbols.gmt). [file 12881_2020_1066_MOESM6_ESM.pdf]

Table S3 GO enrichment results by GSEA for *GSTM2* (c5.all.v6.2.symbols.gmt).

NAME	SIZE	ES	NES	NOM p-val	FDR q-val
GO_NEGATIVE_REGULATION_OF_MITOTIC_NUCLEAR_DIVISION	32	0.668489	2.083699	<0.0001	0.013091
GO_REGULATION_OF_SISTER_CHROMATID_SEGREGATION	61	0.631214	2.088217	<0.0001	0.013201
GO_ANAPHASE_PROMOTING_COMPLEX_DEPENDENT_CATABOLIC_PROCESS	75	0.762444	2.080222	<0.0001	0.013215
GO_UNFOLDED_PROTEIN_BINDING	85	0.61794	2.101719	<0.0001	0.013448
GO_CELLULAR_PROTEIN_COMPLEX_DISASSEMBLY	119	0.696437	2.095502	<0.0001	0.013573
GO_PROTEIN_TRANSPORTER_ACTIVITY	96	0.513712	2.089238	<0.0001	0.013901
GO_POSITIVE_REGULATION_OF_PROTEIN_MODIFICATION_BY_SMALL_PROTEIN_CONJUGATION_OR_REMOVAL	184	0.530921	2.072336	<0.0001	0.014312
GO_PROTEASOMAL_PROTEIN_CATABOLIC_PROCESS	262	0.510697	2.101769	<0.0001	0.014569
GO_REGULATION_OF_CHROMOSOME_SEGREGATION	77	0.625799	2.065479	<0.0001	0.014965
GO_RETROGRADE_VESICLE_MEDIATED_TRANSPORT_GOLGI_TO_ER	75	0.563607	2.106583	<0.0001	0.015003
GO_CHROMOSOME_LOCALIZATION	55	0.635315	2.109664	<0.0001	0.015168
GO_PROTEIN_FOLDING	193	0.541606	2.135797	<0.0001	0.015604
GO_REGULATION_OF_PROTEASOMAL_PROTEIN_CATABOLIC_PROCESS	173	0.478245	2.120341	<0.0001	0.016281
GO_MACROMOLECULAR_COMPLEX_DISASSEMBLY	174	0.616254	2.159528	<0.0001	0.016369
GO_PROTEASOME_COMPLEX	73	0.706195	2.110823	<0.0001	0.016441
GO_REGULATION_OF_NUCLEAR_DIVISION	152	0.489991	2.053254	0.001946	0.017265
GO_NEGATIVE_REGULATION_OF_PROTEIN_MODIFICATION_BY_SMALL_PROTEIN_CONJUGATION_OR_REMOVAL	133	0.57387	2.0479	<0.0001	0.017267
GO_REGULATION_OF_PROTEIN_UBIQUITINATION_INVOLVED_IN_UBIQUITIN_DEPENDENT_PROTEIN_CATABOLIC_PROCESS	101	0.62523	2.042074	<0.0001	0.017317
GO_ATP_DEPENDENT_CHROMATIN_REMODELING	66	0.636228	2.044433	0.001992	0.017408
GO_POSITIVE_REGULATION_OF_CELLULAR_PROTEIN_CATABOLIC_PROCESS	185	0.518435	2.050122	<0.0001	0.017488
GO_SPINDLE_CHECKPOINT	23	0.710218	2.009637	<0.0001	0.017753

**GO_TRANSLATIONAL_ELONGATION**	10	0.73	2.03	<0.0	0.01
	8	9804	161	001	7827
**GO_REGULATION_OF_UBIQUITIN_PROTEIN_LIGASE_ACTIVITY**	17	0.69	2.03	<0.0	0.01
		9845	4521	001	7861
**GO_REGULATION_OF_CELL_CYCLE_PHASE_TRANSITION**	30	0.46	2.02	<0.0	0.01
	7	1738	8355	001	7986
**GO_KINETOCHORE**	10	0.62	2.03	<0.0	0.01
	3	8929	5834	001	8047
**GO_2_IRON_2_SULFUR_CLUSTER_BINDING**	20	0.78	2.00	<0.0	0.01
		8838	9892	001	814
**GO_REGULATION_OF_CELLULAR_PROTEIN_CATABOLIC_PROCESS**	26	0.49	2.12	<0.0	0.01
	2	8432	0352	001	8606
**GO_NEGATIVE_REGULATION_OF_CELL_DIVISION**	56	0.56	2.01	<0.0	0.01
		0643	0039	001	8618
**GO_POSITIVE_REGULATION_OF_LIGASE_ACTIVITY**	10	0.68	2.13	<0.0	0.01
	5	5897	623	001	8725
**GO_MITOCHONDRIAL_TRANSLATION**	10	0.76	2.01	<0.0	0.01
	4	3487	5506	001	8765
**GO_NEGATIVE_REGULATION_OF_NUCLEAR_DIVISION**	42	0.60	2.01	<0.0	0.01
		174	851	001	8814
**GO_CHROMOSOMAL_REGION**	29	0.55	2.01	0.00	0.01
	5	9833	6623	823	8897
**GO_RNA_PHOSPHODIESTER_BOND_HYDROLYSIS**	10	0.54	2.01	0.00	0.01
	7	9019	016	1965	9031
**GO_TRANSCRIPTION_COUPLED_NUCLEOTIDE_EXCISION_REPAIR**	72	0.61	2.01	<0.0	0.01
		5223	1102	001	9108
**GO_REGULATION_OF_PROTEIN_CATABOLIC_PROCESS**	37	0.43	2.01	<0.0	0.01
	6	5746	9515	001	9174
**GO_METAPHASE_PLATE_CONGRESSION**	41	0.65	2.02	<0.0	0.01
		1259	0683	001	9523
**GO_CHROMOSOME_CENTROMERIC_REGION**	15	0.58	2.01	0.00	0.01
	3	4303	1164	1996	963
**GO_NUCLEOBASE_BIOSYNTHETIC_PROCESS**	17	0.74	2.00	0.00	0.01
		9587	1168	1965	9918
**GO_REGULATION_OF_LIGASE_ACTIVITY**	12	0.64	2.16	<0.0	0.02
	5	6547	198	001	0194
**GO_TRANSLATIONAL_TERMINATION**	91	0.78	1.99	<0.0	0.02
		3797	3443	001	08
**GO_TRANSLATION_FACTOR_ACTIVITY_RNA_BINDING**	82	0.57	1.99	<0.0	0.02
		9001	1918	001	0858
**GO_MITOCHONDRIAL_MATRIX**	38	0.58	1.96	<0.0	0.02
	4	3076	2221	001	101
**GO_ORGANELLAR_SMALL_RIBOSOMAL_SUBUNIT**	25	0.81	1.94	<0.0	0.02
		9386	7079	001	1045
**GO_SISTER_CHROMATID_SEGREGATION**	16	0.58	1.94	0.00	0.02
	1	0967	7973	7905	1145
**GO_CYTOSKELETON_DEPENDENT_CYTOKINESIS**	38	0.58	1.98	<0.0	0.02
		4186	8042	001	1175

**GO_CELL_CYCLE_PHASE_TRANSITION**	24	0.49	1.95	0.00	0.02
	7	5002	4892	4024	1186
**GO_ORGANELLE_ENVELOPE_LUMEN**	73	0.59	1.96	<0.0	0.02
		7552	7184	001	123
**GO_DNA_DAMAGE_RESPONSE_DETECTION_OF_DNA_DAMAGE**	36	0.67	1.98	<0.0	0.02
		8741	5925	001	1236
**GO_NUCLEAR_TRANSCRIBED_MRNA_CATABOLIC_PROCESS_EXONUCLEOLYTIC**	30	0.70	1.98	<0.0	0.02
		3513	9533	001	1238
**GO_NEGATIVE_REGULATION_OF_CHROMOSOME_SEGREGATION**	25	0.68	1.99	<0.0	0.02
		3089	3655	001	1261
**GO_CATALYTIC_STEP_2_SPLICEOSOME**	84	0.60	1.95	0.00	0.02
		2059	59	5871	1269
**GO_ER_NUCLEUS_SIGNALING_PATHWAY**	31	0.61	1.96	<0.0	0.02
		0732	2538	001	1293
**GO_REGULATION_OF_CENTROSOME_CYCLE**	38	0.63	1.95	0.00	0.02
		2463	005	2062	1325
**GO_POST_TRANSLATIONAL_PROTEIN_MODIFICATION**	32	0.58	1.94	0.00	0.02
		5724	8245	1923	1362
**GO_PROTEIN_POLYUBIQUITINATION**	23	0.46	1.96	<0.0	0.02
	3	3152	3716	001	1383
**GO_BINDING_OF_SPERM_TO_ZONA_PELLUCIDA**	28	0.62	1.94	<0.0	0.02
		5813	889	001	1406
**GO_REGULATION_OF_PROTEIN_MODIFICATION_BY_SMALL_PROTEIN_CONJUGA**	26	0.46	1.95	<0.0	0.02
**TION_OR_REMOVAL**	5	3356	2802	001	1444
**GO_MITOTIC_NUCLEAR_DIVISION**	33	0.50	1.98	0.00	0.02
	7	4722	1308	3992	1484
**GO_NIK_NF_KAPPAB_SIGNALING**	82	0.62	1.99	0.00	0.02
		9912	4741	1883	1504
**GO_CELLULAR_COMPONENT_DISASSEMBLY**	48	0.41	1.96	<0.0	0.02
	5	7347	4793	001	1525
**GO_REGULATION_OF_MITOTIC_CELL_CYCLE**	44	0.42	1.96	0.00	0.02
	3	3842	7244	1965	1557
**GO_AMIDE_BIOSYNTHETIC_PROCESS**	47	0.56	1.95	0.00	0.02
	9	3962	1319	1894	1576
**GO_MATURATION_OF_SSU_RRNA**	40	0.71	1.95	<0.0	0.02
		1255	5921	001	1577
**GO_RIBOSOME_BIOGENESIS**	29	0.64	1.95	0.00	0.02
	0	1127	0123	578	1613
**GO_NCRNA_METABOLIC_PROCESS**	49	0.58	1.97	0.00	0.02
	6	5088	7377	1965	1681
**GO_CHAPERONE_MEDIATED_PROTEIN_FOLDING**	43	0.58	1.96	<0.0	0.02
		1241	7947	001	1712
**GO_RIBOSOMAL_SMALL_SUBUNIT_BIOGENESIS**	54	0.70	1.95	0.00	0.02
		4716	6601	5758	1741
**GO_PROTEIN_TARGETING**	38	0.46	1.95	0.00	0.02
	6	9614	8645	1828	1749
**GO_RNA_POLYMERASE_COMPLEX**	11	0.53	1.97	<0.0	0.02
	7	1449	1717	001	1766

**GO_TRANSLATION_INITIATION_FACTOR_ACTIVITY**	48	0.658059	1.968536	<0.0001	0.021784
**GO_CONDENSED_CHROMOSOME_CENTROMERIC_REGION**	83	0.646247	1.977977	0.001969	0.021826
**GO_OXIDOREDUCTASE_COMPLEX**	90	0.653503	1.941862	0.005671	0.021834
**GO_CELL_DIVISION**	422	0.468709	1.981618	0.004008	0.021897
**GO_REGULATION_OF_CELLULAR_AMINO_ACID_METABOLIC_PROCESS**	65	0.644133	1.942471	0.005597	0.021903
**GO_REGULATION_OF_CELLULAR_AMINE_METABOLIC_PROCESS**	85	0.589816	1.972502	0.003738	0.021961
**GO_POSITIVE_REGULATION_OF_PROTEIN_CATABOLIC_PROCESS**	253	0.451429	1.969128	<0.0001	0.022007
**GO_ANAPHASE_PROMOTING_COMPLEX**	20	0.707928	1.956751	<0.0001	0.022028
**GO_NUCLEOBASE_CONTAINING_SMALL_MOLECULE_INTERCONVERSION**	21	0.696346	1.940136	<0.0001	0.022165
**GO_NUCLEOTIDE_EXCISION_REPAIR**	111	0.545754	1.942582	<0.0001	0.022177
**GO_ORGANELLAR_RIBOSOME**	67	0.776072	1.938626	<0.0001	0.02234
**GO_ESTABLISHMENT_OF_PROTEIN_LOCALIZATION_TO_ORGANELLE**	344	0.500119	1.972517	0.005525	0.022367
**GO_REGULATION_OF_PROTEASOMAL_UBIQUITIN_DEPENDENT_PROTEIN_CATABOLIC_PROCESS**	141	0.507805	2.175973	<0.0001	0.022495
**GO_NUCLEAR_CHROMOSOME_TELOMERIC_REGION**	121	0.588058	1.972891	0.012397	0.022663
**GO_INTRAMOLECULAR_TRANSFERASE_ACTIVITY**	26	0.670586	1.929936	0.001927	0.022823
**GO_RIBONUCLEOPROTEIN_COMPLEX_BINDING**	89	0.521265	1.930535	<0.0001	0.022942
**GO_CELL_CYCLE_G2_M_PHASE_TRANSITION**	134	0.491143	1.931088	<0.0001	0.023049
**GO_INNATE_IMMUNE_RESPONSE_ACTIVATING_CELL_SURFACE_RECEPTOR_SIGNALING_PATHWAY**	104	0.536971	1.927813	0.001919	0.023158
**GO_CHROMOSOME_SEGREGATION**	244	0.527175	1.926454	0.007874	0.023239
**GO_DNA_TEMPLATED_TRANSCRIPTION_ELONGATION**	86	0.541791	1.925623	<0.0001	0.023261
**GO_ISOPRENOID_BIOSYNTHETIC_PROCESS**	23	0.684919	1.931235	0.001901	0.023286
**GO_DNA_REPLICATION_INDEPENDENT_NUCLEOSOME_ORGANIZATION**	47	0.688533	1.931379	0.006061	0.023554
**GO_POSTTRANSCRIPTIONAL_REGULATION_OF_GENE_EXPRESSION**	413	0.424967	1.933189	0.001916	0.023576
**GO_RRNA_METABOLIC_PROCESS**	243	0.642949	1.922825	0.00578	0.023722

**GO_PROTEIN_TRANSMEMBRANE_TRANSPORT**	49	0.60	1.93	<0.0	0.02
		7839	1802	001	3723
**GO_NCRNA_PROCESSING**	36	0.61	1.92	0.00	0.02
	1	1967	3147	5964	3875
**GO_MIDBODY**	12	0.45	1.91	<0.0	0.02
	1	7856	9208	001	4526
**GO_STEROL_BIOSYNTHETIC_PROCESS**	40	0.67	1.91	0.00	0.02
		945	6774	5825	4731
**GO_ORGANELLE_FISSION**	44	0.46	1.90	0.00	0.02
	0	4813	9314	5976	4732
**GO_REGULATION_OF_TRANSLATIONAL_INITIATION**	80	0.51	1.91	0.00	0.02
		598	7554	1976	4754
**GO_MATURATION_OF_5_8S_RRNA**	28	0.71	1.91	0.00	0.02
		4389	1	1961	4944
**GO_TRANSCRIPTION_ELONGATION_FROM_RNA_POLYMERASE_II_PROMOTER**	71	0.54	1.90	<0.0	0.02
		7268	9349	001	4948
**GO_NUCLEAR_REPLICATION_FORK**	39	0.62	1.90	0.00	0.02
		1693	9411	8247	5168
**GO_ISOMERASE_ACTIVITY**	14	0.47	1.91	<0.0	0.02
	1	5029	1069	001	517
**GO_NUCLEIC_ACID_PHOSPHODIESTER_BOND_HYDROLYSIS**	22	0.47	1.91	0.00	0.02
	9	7997	3892	1957	5287
**GO_MITOTIC_CYTOKINESIS**	31	0.60	1.90	<0.0	0.02
		111	6118	001	5308
**GO_SPLICEOSOMAL_TRI_SNRNP_COMPLEX**	26	0.74	1.91	<0.0	0.02
		9615	4395	001	5324
**GO_HISTONE_EXCHANGE**	45	0.69	1.91	0.00	0.02
		9364	1158	7968	5373
**GO_SPLICEOSOMAL_SNRNP_ASSEMBLY**	37	0.68	1.91	<0.0	0.02
		8364	2502	001	5415
**GO_CHROMOSOME_TELOMERIC_REGION**	14	0.55	1.90	0.01	0.02
	8	8776	6456	6807	5446
**GO_RIBOSOME_ASSEMBLY**	49	0.71	1.91	0.00	0.02
		6855	1368	381	5514
**GO_DNA_DIRECTED_RNA_POLYMERASE_II_HOLOENZYME**	92	0.50	1.90	0.00	0.02
		7219	3011	1965	5528
**GO_RAN_GTPASE_BINDING**	31	0.62	1.90	0.00	0.02
		9552	3673	2058	5598
**GO_RNA_POLYMERASE_ACTIVITY**	43	0.61	1.90	<0.0	0.02
		5069	4261	001	5604
**GO_TRNA_METABOLIC_PROCESS**	16	0.60	1.90	0.00	0.02
	6	6074	1444	6	5749
**GO_MITOTIC_SISTER_CHROMATID_SEGREGATION**	88	0.56	1.90	0.00	0.02
		8778	0161	7905	6071
**GO_PROTEIN_LOCALIZATION_TO_MITOCHONDRION**	62	0.60	1.89	0.00	0.02
		2913	4851	1898	7224
**GO_PROTEIN_MONOUBIQUITINATION**	49	0.52	1.89	<0.0	0.02
		1094	4934	001	7417

GO_BLASTOCYST_DEVELOPMENT	57	0.48	1.89	<0.0	0.02
		9099	527	001	7527
GO_PROTEASOME_BINDING	16	0.69	1.88	0.00	0.02
		3663	4568	3831	8842
GO_RIBOSOME	21	0.68	1.88	0.00	0.02
	2	6449	6582	565	8848
GO_POSITIVE_REGULATION_OF_TELOMERE_MAINTENANCE_VIA_TELOMERE_LE	32	0.61	1.88	0.00	0.02
NGTHENING		1437	7185	5814	8851
GO_FC_EPSILON_RECEPTOR_SIGNALING_PATHWAY	12	0.48	1.88	0.00	0.02
	3	9623	9151	1894	8883
GO_RIBONUCLEASE_ACTIVITY	86	0.51	1.88	0.00	0.02
		3454	5188	3914	8902
GO_RIBONUCLEOPROTEIN_COMPLEX_BIOGENESIS	41	0.57	1.88	0.00	0.02
	2	9464	7514	9747	8952
GO_NUCLEOBASE_METABOLIC_PROCESS	37	0.56	1.88	0.00	0.02
		1475	5277	1996	9086
GO_NUCLEAR_PORE	73	0.54	1.88	0.00	0.02
		4571	7696	5825	9136
GO_90S_PRERIBOSOME	23	0.71	1.88	0.00	0.02
		7956	3178	1996	9236
GO_COENZYME_BIOSYNTHETIC_PROCESS	11	0.53	1.87	0.00	0.02
	2	9461	701	3914	9754
GO_MITOCHONDRIAL_TRANSPORT	16	0.52	1.87	0.00	0.02
	0	2061	5403	3839	9859
GO_TBP_CLASS_PROTEIN_BINDING	20	0.62	1.87	0.00	0.02
		8542	4713	1938	987
GO_PROTEIN_DNA_COMPLEX	14	0.61	1.87	0.03	0.02
	8	1314	7025	4413	9972
GO_POSTREPLICATION_REPAIR	48	0.57	1.87	0.00	0.02
		6122	7611	5871	9973
GO_NUCLEAR_CHROMOSOME_SEGREGATION	20	0.52	1.87	0.01	0.03
	1	9798	554	3917	0015
GO_SNRNA_METABOLIC_PROCESS	74	0.51	1.87	0.00	0.03
		8174	9143	3868	0033
GO_U4_U6_X_U5_TRI_SNRNP_COMPLEX	21	0.72	1.87	<0.0	0.03
		2564	3524	001	0129
GO_ORGANELLE_INNER_MEMBRANE	47	0.56	1.87	0.00	0.03
	8	2687	7651	5747	019
GO_POSITIVE_REGULATION_OF_MRNA_METABOLIC_PROCESS	42	0.52	1.87	0.00	0.03
		1826	2634	207	0218
GO_CELL_REDOX_HOMEOSTASIS	60	0.52	1.87	0.00	0.03
		0218	9207	1916	0238
GO_MATURATION_OF_SSU_RRNA_FROM_TRICISTRONIC_RRNA_TRANSCRIPT_SSU	33	0.70	1.87	0.00	0.03
_RRNA_5_8S_RRNA_LSU_RRNA_		3054	8009	5941	0279
GO_PIGMENT_GRANULE	10	0.48	1.87	0.00	0.03
	0	1577	0711	3774	0835
GO_PRERIBOSOME	58	0.66	1.86	<0.0	0.03
		9593	9399	001	1072

**GO_CYTOPLASMIC_TRANSLATION**	40	0.708383	1.867947	0.007619	0.031152
**GO_ORGANELLAR_LARGE_RIBOSOMAL_SUBUNIT**	30	0.792195	1.868544	0.001961	0.031158
**GO_REGULATION_OF_TELOMERASE_RNA_LOCALIZATION_TO_CAJAL_BODY**	15	0.876268	1.867178	<0.0001	0.031209
**GO_SPINDLE**	265	0.431364	1.866385	0.003891	0.03123
**GO_PROTEASOME_ACCESSORY_COMPLEX**	23	0.789166	1.864973	<0.0001	0.031235
**GO_METHIONINE_METABOLIC_PROCESS**	18	0.615156	1.865478	0.001876	0.031278
**GO_NUCLEOLAR_PART**	59	0.640111	1.86394	0.002	0.03136
**GO_MITOCHONDRIAL_MEMBRANE_ORGANIZATION**	90	0.513828	1.862918	<0.0001	0.031494
**GO_NCRNA_TRANSCRIPTION**	80	0.497217	1.86116	0.001965	0.031884
**GO_NUCLEOTIDYLTRANSFERASE_ACTIVITY**	121	0.478182	1.860399	<0.0001	0.03197
**GO_DNA_STRAND_ELONGATION**	29	0.687929	1.858867	0.007921	0.032311
**GO_PHOSPHATASE_COMPLEX**	43	0.533063	1.857676	0.006024	0.032483
**GO_COPI_COATED_VESICLE_MEMBRANE**	16	0.726294	1.856385	0.001876	0.032776
**GO_METAL_CLUSTER_BINDING**	60	0.546	1.85437	0.005792	0.033318
**GO_MITOCHONDRIAL_PROTEIN_COMPLEX**	126	0.693346	1.85371	0.007648	0.033355
**GO_REGULATION_OF_RNA_STABILITY**	134	0.578709	2.189253	<0.0001	0.033688
**GO_PROTEIN_LOCALIZATION_TO_CHROMOSOME**	37	0.566554	1.84984	0.009671	0.034505
**GO_SISTER_CHROMATID_COHESION**	98	0.590175	1.848895	0.023211	0.034598
**GO_PROTEIN_TARGETING_TO_MITOCHONDRION**	46	0.653206	1.850003	0.00381	0.034612
**GO_INTRINSIC_COMPONENT_OF_MITOCHONDRIAL_MEMBRANE**	43	0.594242	1.847994	0.003824	0.034755
**GO_CONDENSED_CHROMOSOME**	166	0.534777	1.847275	0.016293	0.034841
**GO_ASPARTATE_FAMILY_AMINO_ACID_BIOSYNTHETIC_PROCESS**	22	0.62603	1.843032	0.003738	0.036044
**GO_SUBSTANTIA_NIGRA_DEVELOPMENT**	44	0.537297	1.843471	0.001848	0.036109
**GO_POSITIVE_REGULATION_OF_CANONICAL_WNT_SIGNALING_PATHWAY**	115	0.46127	1.841265	0.003929	0.036481

**GO_GLYCOSYL_COMPOUND_BIOSYNTHETIC_PROCESS**	10	0.53	1.83	0.00	0.03
	7	4843	561	7605	6787
**GO_ANTIGEN_PROCESSING_AND_PRESENTATION_OF_PEPTIDE_ANTIGEN**	16	0.51	1.83	0.00	0.03
	8	5215	6815	1927	6919
**GO_POSITIVE_REGULATION_OF_DNA_BIOSYNTHETIC_PROCESS**	57	0.48	1.83	<0.0	0.03
		5319	9437	001	6924
**GO_MITOCHONDRIAL_RNA_METABOLIC_PROCESS**	23	0.70	1.83	0.00	0.03
		0868	8911	4049	6925
**GO_REGULATION_OF_ESTABLISHMENT_OF_PLANAR_POLARITY**	10	0.48	1.83	0.00	0.03
	9	105	574	7449	6937
**GO_SMN_SM_PROTEIN_COMPLEX**	17	0.77	1.83	0.00	0.03
		708	2455	1905	6995
**GO_COENZYME_METABOLIC_PROCESS**	24	0.48	1.83	0.00	0.03
	3	7834	6086	5871	7007
**GO_TRNA_PROCESSING**	10	0.60	1.83	0.00	0.03
	6	0474	8189	994	7012
**GO_POSITIVE_REGULATION_OF_GENE_EXPRESSION_EPIGENETIC**	75	0.58	1.83	0.03	0.03
		4821	7174	4483	7015
**GO__DE_NOVO_PROTEIN_FOLDING**	17	0.65	1.83	0.00	0.03
		0085	1824	1961	7068
**GO_CENTROMERE_COMPLEX_ASSEMBLY**	42	0.69	1.83	0.01	0.03
		1698	7426	9841	7117
**GO_DNA_DEPENDENT_DNA_REPLICATION**	89	0.58	1.83	0.02	0.03
		5006	3259	3857	7145
**GO_NUCLEOID**	39	0.63	1.83	0.00	0.03
		3483	1109	3781	7188
**GO_SIGNAL_SEQUENCE_BINDING**	36	0.53	1.83	0.00	0.03
		0819	2462	1908	7202
**GO_NUCLEOTIDE_EXCISION_REPAIR_DNA_DAMAGE_RECOGNITION**	23	0.66	1.83	0.00	0.03
		0999	3278	7449	7347
**GO_REPLISOME**	29	0.61	1.83	0.01	0.03
		0251	3703	5968	7355
**GO_INNER_MITOCHONDRIAL_MEMBRANE_PROTEIN_COMPLEX**	99	0.72	1.82	0.00	0.03
		5779	8773	947	7571
**GO_NEGATIVE_REGULATION_OF_CELLULAR_PROTEIN_CATABOLIC_PROCESS**	60	0.46	1.82	0.00	0.03
		6931	9088	198	7665
**GO_POSITIVE_REGULATION_OF_TELOMERE_MAINTENANCE**	44	0.53	1.82	0.01	0.03
		2948	945	1788	7723
**GO_EUKARYOTIC_TRANSLATION_INITIATION_FACTOR_3_COMPLEX**	16	0.73	1.82	0.00	0.03
		2484	3934	3802	7953
**GO_CELL_CYCLE_CHECKPOINT**	17	0.45	1.82	0.01	0.03
	8	1058	3032	3725	8074
**GO_CLEAVAGE_INVOLVED_IN_RRNA_PROCESSING**	19	0.72	1.82	<0.0	0.03
		4964	398	001	8149
**GO_NUCLEOSOMAL_DNA_BINDING**	29	0.60	1.82	0.01	0.03
		0539	6526	3752	815
**GO_FORMATION_OF_TRANSLATION_PREINITIATION_COMPLEX**	20	0.71	1.82	0.02	0.03
		0563	0898	0755	8209

**GO_NUCLEAR_EUCHROMATIN**	23	0.58	1.82	0.00	0.03
		394	6742	4073	8236
**GO_ERROR_FREE_TRANSLESION_SYNTHESIS**	17	0.72	1.81	0.00	0.03
		4546	9512	1852	8253
**GO_REGULATION_OF_CENTROSOME_DUPLICATION**	31	0.58	1.82	0.00	0.03
		721	5206	6186	8255
**GO_VIRAL_LIFE_CYCLE**	27	0.47	1.82	0.02	0.03
	6	0187	1161	0522	83
**GO_ERAD_PATHWAY**	70	0.47	1.82	0.01	0.03
		8927	3981	1765	835
**GO_TOXIN_TRANSPORT**	36	0.52	1.81	0.00	0.03
		3448	9653	1946	8403
**GO_MATURATION_OF_5_8S_RRNA_FROM_TRICISTRONIC_RRNA_TRANSCRIPT_SS**	19	0.71	1.82	0.00	0.03
**U_RRNA_5_8S_RRNA_LSU_RRNA_**		7845	0007	3831	8404
**GO_TELOMERE_ORGANIZATION**	10	0.55	1.82	0.02	0.03
	1	3953	5297	268	8417
**GO_PROTEIN_DNA_COMPLEX_SUBUNIT_ORGANIZATION**	20	0.52	1.82	0.03	0.03
	2	8413	1186	0675	8483
**GO_MICROBODY_MEMBRANE**	57	0.52	1.82	0.00	0.03
		0972	4118	3788	8519
**GO_ANTIGEN_PROCESSING_AND_PRESENTATION_OF_EXOGENOUS_PEPTIDE_AN**	64	0.64	1.82	0.00	0.03
**TIGEN_VIA_MHC_CLASS_I**		0468	1454	5792	8555
**GO_INTRINSIC_COMPONENT_OF_MITOCHONDRIAL_INNER_MEMBRANE**	16	0.74	1.81	0.00	0.03
		1847	472	5714	8622
**GO_PTERIDINE_CONTAINING_COMPOUND_METABOLIC_PROCESS**	34	0.58	1.81	0.01	0.03
		3972	7941	0204	867
**GO_RNA_PHOSPHODIESTER_BOND_HYDROLYSIS_ENDONUCLEOLYTIC**	54	0.54	1.81	0.00	0.03
		9231	486	8	8746
**GO_DNA_CONFORMATION_CHANGE**	23	0.52	1.81	0.03	0.03
	5	3488	4986	0181	8883
**GO_MITOCHONDRIAL_MEMBRANE_PART**	15	0.63	1.81	0.01	0.03
	5	577	5228	7241	8968
**GO_COFACTOR_BIOSYNTHETIC_PROCESS**	15	0.52	1.81	0.00	0.03
	1	7045	6678	7797	9001
**GO_U12_TYPE_SPLICEOSOMAL_COMPLEX**	25	0.67	1.81	0.00	0.03
		3989	535	7859	9117
**GO_DNA_STRAND_ELONGATION_INVOLVED_IN_DNA_REPLICATION**	25	0.69	1.81	0.00	0.03
		8615	5478	7828	9255
**GO_SMALL_SUBUNIT_PROCESSOME**	32	0.65	1.81	0.00	0.03
		1477	2455	9901	9373
**GO_FOLIC_ACID_METABOLIC_PROCESS**	17	0.66	1.81	0.01	0.03
		3046	1629	0246	9499
**GO_NUCLEOSIDE_MONOPHOSPHATE_BIOSYNTHETIC_PROCESS**	77	0.56	1.81	0.01	0.03
		6373	0189	5152	9889
**GO_HISTONE_MRNA_METABOLIC_PROCESS**	28	0.61	1.80	0.00	0.03
		8324	969	3984	9895
**GO_DNA_PACKAGING**	16	0.55	1.80	0.03	0.04
	0	6676	8974	2258	0023

GO_ER_ASSOCIATED_UBIQUITIN_DEPENDENT_PROTEIN_CATABOLIC_PROCESS	59	0.489475	1.807057	0.011561	0.040469
GO_AMINO_ACID_ACTIVATION	51	0.638506	1.80728	0.006085	0.04058
GO_U5_SNRNP	16	0.719073	1.803703	0.007477	0.040624
GO_INTRAMOLECULAR_OXIDOREDUCTASE_ACTIVITY_TRANSPOSING_S_S_BONDS	19	0.648904	1.803017	0.022346	0.040722
GO_DNA_REPAIR	431	0.455486	1.801092	0.017717	0.040746
GO_SINGLE_STRANDED_DNA_BINDING	77	0.530716	1.806106	0.02449	0.040752
GO_NUCLEOSIDE_TRIPHOSPHATE_METABOLIC_PROCESS	212	0.550799	1.803742	0.022945	0.040794
GO_NCRNA_3_END_PROCESSING	20	0.70774	1.801274	0.005515	0.040858
GO_PTERIDINE_CONTAINING_COMPOUND_BIOSYNTHETIC_PROCESS	16	0.751901	1.801412	0.001976	0.04096
GO_ANTIGEN_PROCESSING_AND_PRESENTATION_OF_PEPTIDE_ANTIGEN_VIA_MHC_CLASS_I	89	0.573187	1.803752	0.011628	0.040976
GO_RESPIRATORY_CHAIN	76	0.728519	1.805055	0.01341	0.040982
GO_REPLICATION_FORK	62	0.573263	1.801516	0.028226	0.041125
GO_SMALL_NUCLEOLAR_RIBONUCLEOPROTEIN_COMPLEX	18	0.766301	1.803828	0.005848	0.041144
GO_SNORNA_BINDING	25	0.653021	1.804185	0.003891	0.04118
GO_THIOESTER_METABOLIC_PROCESS	73	0.501295	1.797941	0.005848	0.041472
GO_POSITIVE_REGULATION_OF_PROTEASOMAL_PROTEIN_CATABOLIC_PROCESS	95	0.417895	1.797562	0.003781	0.041477
GO_ARP2_3_COMPLEX_MEDIATED_ACTIN_NUCLEATION	15	0.6671	1.798738	0.007339	0.041498
GO_U2_SNRNP	18	0.710125	1.797036	0.00956	0.041505
GO_MICROBODY_PART	90	0.497334	1.798109	0.003831	0.041578
GO_NUCLEAR_ENVELOPE_ORGANIZATION	75	0.464063	1.792515	0.005682	0.043127
GO_REGULATION_OF_CELL_DIVISION	246	0.390434	1.791639	0.003899	0.043287
GO_ENERGY_DERIVATION_BY_OXIDATION_OF_ORGANIC_COMPOUNDS	203	0.526526	1.790675	0.017408	0.043329
GO_POSITIVE_REGULATION_OF_CHROMOSOME_SEGREGATION	23	0.602177	1.78998	0.007843	0.043424
GO_CELL_CYCLE_G1_S_PHASE_TRANSITION	107	0.515449	1.790708	0.015968	0.04349

**GO_NUCLEOSIDE_MONOPHOSPHATE_METABOLIC_PROCESS**	22	0.53	1.78	0.02	0.04
	5	4808	912	4809	3635
**GO_RIBOSOMAL_SUBUNIT**	15	0.70	1.78	0.01	0.04
	5	8863	8014	5009	3902
**GO_RRNA_MODIFICATION**	23	0.66	1.78	0.00	0.04
		1941	6361	7843	3933
**GO_POSITIVE_REGULATION_OF_MRNA_PROCESSING**	30	0.53	1.78	0.00	0.04
		989	7117	2033	3968
**GO_DNA_BIOSYNTHETIC_PROCESS**	10	0.49	1.78	0.02	0.04
	7	8629	6634	004	4014
**GO_RIBOSOMAL_LARGE_SUBUNIT_BIOGENESIS**	48	0.67	1.78	0.01	0.04
		3904	7348	3725	4067
**GO_GLOBAL_GENOME_NUCLEOTIDE_EXCISION_REPAIR**	32	0.58	1.78	0.01	0.04
		6182	4917	1811	4138
**GO_NUCLEAR_CHROMOSOME**	48	0.42	1.78	0.02	0.04
	1	6081	4073	2541	4314
**GO_PROTEIN_K11_LINKED_UBIQUITINATION**	26	0.61	1.78	0.01	0.04
		2417	4936	7613	4315
**GO_PURINE_NUCLEOBASE_METABOLIC_PROCESS**	19	0.62	1.78	0.00	0.04
		9319	2866	7874	465
**GO_MITOTIC_CELL_CYCLE_CHECKPOINT**	12	0.44	1.78	0.01	0.04
	9	438	1321	5968	5189
**GO_RNA_MODIFICATION**	10	0.53	1.78	0.01	0.04
	6	0286	053	0163	5306
**GO_CYTOKINESIS**	80	0.44	1.77	<0.0	0.04
		1509	9308	001	5329
**GO_SITE_OF_DOUBLE_STRAND_BREAK**	28	0.58	1.77	0.00	0.04
		2529	8888	5906	5391
**GO_GDP_BINDING**	47	0.56	1.77	0.01	0.04
		7327	9789	3109	5471
**GO_PROTEIN_IMPORT**	15	0.39	1.77	<0.0	0.04
	0	8099	9322	001	551
**GO_TRANSLATION_PREINITIATION_COMPLEX**	16	0.70	1.77	0.01	0.04
		9374	5868	5355	5614
**GO_RRNA_METHYLATION**	15	0.69	1.77	0.00	0.04
		3086	6684	3891	5635
**GO_SULFUR_AMINO_ACID_BIOSYNTHETIC_PROCESS**	19	0.58	1.77	0.01	0.04
		3731	6848	1472	5744
**GO_MEMBRANE_BUDDING**	11	0.44	1.77	0.00	0.04
	1	6504	588	9259	5791
**GO_SPINDLE_MICROTUBULE**	56	0.50	1.77	0.01	0.04
		5879	7024	9531	5855
**GO_GENE_SILENCING**	17	0.48	1.77	0.01	0.04
	8	6159	7114	8367	6002
**GO_REGULATION_OF_TRANSLATIONAL_ELONGATION**	22	0.61	1.77	0.01	0.04
		3476	3498	1928	6072
**GO_PROTEIN_REFOLDING**	19	0.64	1.77	0.01	0.04
		1092	3819	9569	6113

GO_PSEUDOURIDINE_SYNTHESIS	17	0.73	1.77	0.00	0.04
		9604	4006	7952	6246
GO_PRECATALYTIC_SPLICEOSOME	21	0.70	1.77	0.01	0.04
		8901	1816	7787	6466
GO_RIBONUCLEOPROTEIN_COMPLEX_SUBUNIT_ORGANIZATION	18	0.51	1.77	0.01	0.04
	4	192	1273	5748	6523
GO_MULTI_ORGANISM_LOCALIZATION	65	0.51	1.77	0.01	0.04
		3364	2034	7964	6551
GO_COPI_COATED_VESICLE	22	0.61	1.76	0.00	0.04
		293	9871	3683	6643
GO_PURINE_NUCLEOSIDE_BIOSYNTHETIC_PROCESS	81	0.54	1.77	0.01	0.04
		1753	0196	8939	6668
GO_ACTIN_NUCLEATION	21	0.58	1.77	0.01	0.04
		0673	03	105	6784
GO_THREONINE_TYPE_PEPTIDASE_ACTIVITY	20	0.77	1.76	0.01	0.04
		0057	8721	5474	6932
GO_RNA_CATABOLIC_PROCESS	21	0.52	1.76	0.03	0.04
	6	0492	7472	1599	7292
GO_NUCLEOBASE_CONTAINING_SMALL_MOLECULE_METABOLIC_PROCESS	49	0.43	1.76	0.01	0.04
	5	2328	3862	1538	8266
GO_DNA_REPLICATION_DEPENDENT_NUCLEOSOME_ORGANIZATION	31	0.79	1.76	0.03	0.04
		2031	3258	4413	8352
GO_MRNA_CLEAVAGE_FACTOR_COMPLEX	17	0.61	1.76	0.01	0.04
		4473	3883	8145	8435
GO_CELLULAR_RESPIRATION	13	0.61	1.76	0.02	0.04
	5	1804	4173	1113	8437
GO_LARGE_RIBOSOMAL_SUBUNIT	90	0.70	1.76	0.01	0.04
		9777	2659	6949	8479
GO_EXONUCLEASE_ACTIVITY_ACTIVE_WITH_EITHER_RIBO_OR_DEOXYRIBONU	40	0.55	1.76	0.01	0.04
CLEIC_ACIDS_AND_PRODUCING_5_PHOSPHOMONOESTERS		5239	4381	7647	8522
GO_ELECTRON_TRANSPORT_CHAIN	91	0.65	1.76	0.02	0.04
		6341	126	3033	8875
GO_POSITIVE_REGULATION_OF_WNT_SIGNALING_PATHWAY	14	0.39	1.75	0.00	0.04
	8	6685	9586	1876	9429
GO_MITOCHONDRIAL_RESPIRATORY_CHAIN_COMPLEX_ASSEMBLY	63	0.69	1.75	0.01	0.04
		7593	861	7408	9433
GO_NUCLEOTIDE_EXCISION_REPAIR_DNA_INCISION	39	0.56	1.75	0.01	0.04
		3784	6235	4028	9545
GO_RIBOSOMAL_LARGE_SUBUNIT_ASSEMBLY	23	0.72	1.75	0.01	0.04
		002	8619	1385	961
GO_TRANSLESION_SYNTHESIS	36	0.56	1.75	0.01	0.04
		331	6323	3462	9666
GO_RNA_METHYLATION	45	0.57	1.75	0.01	0.04
		0979	7244	2072	9713
GO_CHROMATIN_ASSEMBLY_OR_DISASSEMBLY	14	0.53	1.75	0.04	0.04
	9	7587	6896	4534	9727
GO_POSITIVE_REGULATION_OF_CATABOLIC_PROCESS	38	0.35	1.75	<0.0	0.04
	1	9826	7466	001	9788

GO_OLIGOSACCHARIDE_LIPID_INTERMEDIATE_BIOSYNTHETIC_PROCESS	20	0.63	1.75	0.01	0.04
		3198	4985	1538	9796
GO_ENDORIBONUCLEASE_ACTIVITY_PRODUCING_5_PHOSPHOMONOESTERS	26	0.64	1.75	0.01	0.04
		2285	5302	3944	9813
GO_CHROMATIN_REMODELING	13	0.43	1.75	0.01	0.04
	9	6501	6361	2072	9823
GO_VESICLE_TARGETING	75	0.44	1.75	0.00	0.04
		8625	4485	7168	9838
GO_NADH_DEHYDROGENASE_COMPLEX	42	0.74	1.75	0.01	0.05
		1105	273	6854	0426
GO_ANTIGEN_PROCESSING_AND_PRESENTATION_OF_PEPTIDE_OR_POLYSACCH	87	0.47	1.75	0.00	0.05
ARIDE_ANTIGEN_VIA_MHC_CLASS_II		6083	1042	9615	113
GO_OXIDOREDUCTASE_ACTIVITY_ACTING_ON_NAD_P_H	89	0.56	1.75	0.02	0.05
		3263	0693	1277	1138
GO_MITOCHONDRIAL_RESPIRATORY_CHAIN_COMPLEX_I_BIOGENESIS	51	0.72	1.75	0.01	0.05
		1953	0219	5238	1179
GO_POLYSOME	38	0.52	1.74	0.00	0.05
		6773	9621	7874	1253
GO_ALPHA_AMINO_ACID_BIOSYNTHETIC_PROCESS	72	0.48	1.74	0.00	0.05
		75	9018	789	1345
GO_PYRIMIDINE_NUCLEOTIDE_BIOSYNTHETIC_PROCESS	28	0.58	1.74	0.01	0.05
		535	828	6575	1514
GO_SMALL_RIBOSOMAL_SUBUNIT	65	0.71	1.74	0.01	0.05
		3637	7845	8657	1532
GO_REGULATION_OF_MITOCHONDRIAL_OUTER_MEMBRANE_PERMEABILIZATI	42	0.52	1.74	0.00	0.05
ON_INVOLVED_IN_APOPTOTIC_SIGNALING_PATHWAY		3261	7546	9709	1547
GO_FC_RECEPTOR_SIGNALING_PATHWAY	18	0.42	1.74	0.00	0.05
	2	814	6727	3781	1729
GO_TETRAHYDROFOLATE_METABOLIC_PROCESS	19	0.61	1.74	0.01	0.05
		9939	5875	227	1923
GO_RNA_POLYADENYLATION	28	0.57	1.74	0.01	0.05
		1329	5284	8036	2029
GO_DNA_INTEGRITY_CHECKPOINT	13	0.43	1.74	0.02	0.05
	5	7905	4686	3857	216
GO_CELLULAR_MODIFIED_AMINO_ACID_BIOSYNTHETIC_PROCESS	49	0.50	1.74	0.00	0.05
		7436	3549	6012	2416
GO_FOLIC_ACID_CONTAINING_COMPOUND_METABOLIC_PROCESS	27	0.57	1.74	0.01	0.05
		1753	3826	8256	2451
GO_PROTEIN_UBIQUITINATION_INVOLVED_IN_UBIQUITIN_DEPENDENT_PROTEI	12	0.41	1.74	0.00	0.05
N_CATABOLIC_PROCESS	7	0361	203	1916	2496
GO_COFACTOR_METABOLIC_PROCESS	31	0.46	1.74	0.01	0.05
	1	8728	1544	3917	2545
GO_TRANSLATIONAL_INITIATION	14	0.64	1.74	0.03	0.05
	2	2859	2438	525	2582
GO_DISULFIDE_OXIDOREDUCTASE_ACTIVITY	27	0.59	1.74	0.01	0.05
		4315	2854	3359	2587
GO_POSITIVE_REGULATION_OF_PROTEOLYSIS	35	0.38	1.74	0.00	0.05
	1	6207	2149	3953	2612

**GO_CELLULAR_PROTEIN_COMPLEX_ASSEMBLY**	31	0.38	1.73	0.00	0.05
	3	5198	9793	566	2909
**GO_DNA_REPLICATION**	19	0.49	1.73	0.04	0.05
	0	3729	9967	6278	2996
**GO_MEDIATOR_COMPLEX**	34	0.48	1.73	0.00	0.05
		9712	9982	9615	3158
**GO_THIOESTER_BIOSYNTHETIC_PROCESS**	46	0.51	1.73	0.01	0.05
		514	8187	5326	3453
**GO_ANTIGEN_PROCESSING_AND_PRESENTATION**	20	0.47	1.73	0.01	0.05
	4	3604	7715	7308	3529
**GO_DNA_TEMPLATED_TRANSCRIPTION_TERMINATION**	97	0.52	1.73	0.02	0.05
		298	6272	7397	3867
**GO_NUCLEOSIDE_PHOSPHATE_BIOSYNTHETIC_PROCESS**	17	0.43	1.73	0.00	0.05
	1	5221	654	9452	3915
**GO_NUCLEAR_TRANSCRIBED_MRNA_CATABOLIC_PROCESS_DEADENYLATION_D**	53	0.49	1.73	0.00	0.05
**EPENDENT_DECAY**		2428	3687	9615	4515
**GO_NUCLEAR_EXOSOME_RNASE_COMPLEX_**	15	0.69	1.73	0.01	0.05
		5562	3157	5123	4607
**GO_NUCLEUS_ORGANIZATION**	12	0.42	1.73	0.00	0.05
	1	4566	3795	2	4639
**GO_GENE_SILENCING_BY_RNA**	12	0.49	1.73	0.02	0.05
	4	4599	3864	8689	4776
**GO_NUCLEASE_ACTIVITY**	18	0.43	1.73	0.01	0.05
	5	094	0416	1765	4912
**GO_NUCLEAR_TRANSPORT**	33	0.37	1.73	0.00	0.05
	9	1201	2053	198	4923
**GO_GLYCOSYL_COMPOUND_METABOLIC_PROCESS**	34	0.46	1.72	0.02	0.05
	4	2699	9856	1277	5029
**GO_OXIDATIVE_PHOSPHORYLATION**	79	0.69	1.73	0.02	0.05
		442	0457	277	5067
**GO_POSITIVE_REGULATION_OF_MITOTIC_NUCLEAR_DIVISION**	46	0.46	1.73	0.00	0.05
		1063	0644	5988	512
**GO_POSITIVE_REGULATION_OF_MITOCHONDRIAL_OUTER_MEMBRANE_PERME**	36	0.52	1.72	0.01	0.05
**ABILIZATION_INVOLVED_IN_APOPTOTIC_SIGNALING_PATHWAY**		6542	6663	5656	6034
**GO_MEMBRANE_DISASSEMBLY**	44	0.52	1.72	0.02	0.05
		9708	7346	1696	6062
**GO_PRENYLTRANSFERASE_ACTIVITY**	15	0.64	1.72	0.01	0.05
		6675	6943	1719	6074
**GO_DEOXYRIBONUCLEOSIDE_TRIPHOSPHATE_METABOLIC_PROCESS**	16	0.68	1.72	0.01	0.05
		2091	6021	6129	6144
**GO_EXOSOME_RNASE_COMPLEX_**	21	0.62	1.72	0.01	0.05
		8125	2284	7476	7804
**GO_ENDOPLASMIC_RETICULUM_ORGANIZATION**	36	0.51	1.72	0.01	0.05
		6354	0302	7143	7845
**GO_RNA_PHOSPHODIESTER_BOND_HYDROLYSIS_EXONUCLEOLYTIC**	33	0.56	1.72	0.00	0.05
		1503	0863	994	7881
**GO_POSITIVE_REGULATION_OF_VIRAL_RELEASE_FROM_HOST_CELL**	15	0.64	1.72	0.01	0.05
		5509	1116	3619	7915

**GO_REGULATION_OF_CELLULAR_RESPONSE_TO_HEAT**	72	0.459761	1.721735	0.013699	0.057935
**GO_PURINE_NUCLEOSIDE_MONOPHOSPHATE_BIOSYNTHETIC_PROCESS**	57	0.556637	1.720314	0.029466	0.058015
**GO_TELOMERE_MAINTENANCE_VIA_RECOMBINATION**	32	0.631772	1.721143	0.028056	0.058074
**GO_DNA_SYNTHESIS_INVOLVED_IN_DNA_REPAIR**	67	0.53651	1.718964	0.034343	0.058314
**GO_MITOCHONDRIAL_TRANSMEMBRANE_TRANSPORT**	52	0.616714	1.718512	0.026515	0.058341
**GO_NEGATIVE_REGULATION_OF_PROTEIN_CATABOLIC_PROCESS**	103	0.37905	1.714341	<0.0001	0.059577
**GO_NUCLEAR_IMPORT**	126	0.406894	1.714692	0.004	0.059593
**GO_MITOTIC_RECOMBINATION**	41	0.603166	1.71318	0.044088	0.059664
**GO_FATTY_ACYL_COA_METABOLIC_PROCESS**	45	0.496694	1.715152	0.017578	0.059718
**GO_LIGASE_ACTIVITY_FORMING_CARBON_OXYGEN_BONDS**	44	0.601403	1.714747	0.024048	0.059729
**GO_REGULATION_OF_PROTEIN_STABILITY**	208	0.367098	1.713363	0.001905	0.059765
**GO_CELLULAR_AMINO_ACID_METABOLIC_PROCESS**	312	0.408881	1.7153	0.006135	0.059822
**GO_PEPTIDYL_PROLINE_MODIFICATION**	45	0.49611	1.713469	0.009671	0.059873
**GO_CELL_SEPARATION_AFTER_CYTOKINESIS**	17	0.620864	1.709761	0.01581	0.060884
**GO_NUCLEAR_ENVELOPE_REASSEMBLY**	17	0.60282	1.710454	0.016886	0.060909
**GO_DOUBLE_STRAND_BREAK_REPAIR**	150	0.492757	1.709863	0.04251	0.061009
**GO_RNA_CAPPING**	34	0.593629	1.707469	0.018036	0.061878
**GO_REGULATION_OF_CELLULAR_KETONE_METABOLIC_PROCESS**	165	0.406132	1.705157	0.005736	0.062893
**GO_VIRION_ASSEMBLY**	36	0.553808	1.704134	0.015444	0.063273
**GO_SULFUR_AMINO_ACID_METABOLIC_PROCESS**	40	0.466447	1.703129	0.009634	0.063354
**GO_THIOLESTER_HYDROLASE_ACTIVITY**	30	0.544642	1.703512	0.012024	0.063486
**GO_INTRAMOLECULAR_OXIDOREDUCTASE_ACTIVITY**	49	0.495098	1.703176	0.011299	0.0635
**GO_TRICARBOXYLIC_ACID_METABOLIC_PROCESS**	36	0.585055	1.701307	0.030075	0.064195
**GO_ELECTRON_CARRIER_ACTIVITY**	106	0.514399	1.700664	0.027613	0.064409

**GO_RRNA_BINDING**	54	0.64	1.69	0.03	0.06
		0086	8405	0132	5239
**GO_DNA_GEOMETRIC_CHANGE**	77	0.51	1.69	0.03	0.06
		7813	7891	75	532
**GO_STRUCTURAL_CONSTITUENT_OF_RIBOSOME**	19	0.61	1.69	0.04	0.06
	9	6637	8544	4776	5347
**GO_REGULATION_OF_CYTOKINESIS**	54	0.46	1.69	0.01	0.06
		3927	7494	6064	5356
**GO_REGULATION_OF_PROTEIN_PHOSPHATASE_TYPE_2A_ACTIVITY**	23	0.52	1.69	0.01	0.06
		0077	6194	378	5481
**GO_REGULATION_OF_PROTEIN_INSERTION_INTO_MITOCHONDRIAL_MEMBRAN**	29	0.52	1.69	0.01	0.06
**E_INVOLVED_IN_APOPTOTIC_SIGNALING_PATHWAY**		6801	6339	6032	5596
**GO_SINGLE_ORGANISM_MEMBRANE_BUDDING**	71	0.45	1.69	0.00	0.06
		1369	659	9158	5638
**GO_NUCLEOTIDE_EXCISION_REPAIR_DNA_GAP_FILLING**	24	0.59	1.69	0.04	0.06
		9159	545	3152	5783
**GO_POSITIVE_REGULATION_OF_TELOMERASE_ACTIVITY**	27	0.51	1.69	0.00	0.06
		2194	5009	7707	5879
**GO_PROTEIN_AUTOUBIQUITINATION**	47	0.46	1.69	0.00	0.06
		2582	3629	7797	6227
**GO_OXIDOREDUCTASE_ACTIVITY_ACTING_ON_NAD_P_H_QUINONE_OR_SIMILAR**	51	0.66	1.69	0.03	0.06
**_COMPOUND_AS_ACCEPTOR**		9626	3909	8388	6265
**GO_PURINE_CONTAINING_COMPOUND_SALVAGE**	15	0.62	1.69	0.02	0.06
		5984	265	2857	6598
**GO_ORGANOPHOSPHATE_ESTER_TRANSMEMBRANE_TRANSPORTER_ACTIVITY**	23	0.54	1.69	0.02	0.06
		9278	122	4096	6806
**GO_PYRIMIDINE_CONTAINING_COMPOUND_BIOSYNTHETIC_PROCESS**	37	0.53	1.69	0.00	0.06
		3614	1254	7648	6965
**GO_ER_TO_GOLGI_VESICLE_MEDIATED_TRANSPORT**	16	0.39	1.69	0.01	0.06
	0	6738	0486	2844	7057
**GO_CYTOPLASMIC_EXOSOME_RNASE_COMPLEX_**	15	0.66	1.69	0.02	0.06
		1908	1298	1359	7121
**GO_REGULATION_OF_DNA_REPLICATION**	15	0.38	1.68	0.01	0.06
	3	4988	9035	3672	7296
**GO_NEGATIVE_REGULATION_OF_TRANSLATIONAL_INITIATION**	20	0.56	1.68	0.02	0.06
		1435	9651	0992	7315
**GO_ENDORIBONUCLEASE_ACTIVITY**	45	0.51	1.68	0.02	0.06
		2007	922	0121	7367
**GO_CIS_TRANS_ISOMERASE_ACTIVITY**	37	0.50	1.68	0.02	0.06
		8259	7115	3166	7682
**GO_CYTOSOLIC_PART**	20	0.51	1.68	0.04	0.06
	8	3678	7287	9632	778
**GO_REGULATION_OF_INTRINSIC_APOPTOTIC_SIGNALING_PATHWAY_BY_P53_CL**	21	0.55	1.68	0.02	0.06
**ASS_MEDIATOR**		6188	7518	7613	784
**GO_DNA_REPLICATION_INITIATION**	28	0.66	1.68	0.03	0.06
		7767	7768	6	7869
**GO_SPINDLE_POLE**	11	0.43	1.68	0.03	0.06
	2	3996	6284	1558	7967

**GO_UBIQUITIN_LIKE_PROTEIN_CONJUGATING_ENZYME_ACTIVITY**	29	0.56	1.68	0.02	0.06
		5357	5697	7668	8093
**GO_GENERATION_OF_PRECURSOR_METABOLITES_AND_ENERGY**	26	0.47	1.68	0.03	0.06
	9	2545	4657	3074	8531
**GO_PEROXISOMAL_TRANSPORT**	18	0.56	1.68	0.01	0.06
		8867	3753	0101	8798
**GO_MULTIVESICULAR_BODY_ORGANIZATION**	30	0.57	1.68	0.02	0.06
		4903	2279	2044	9492
**GO_ASPARTATE_FAMILY_AMINO_ACID_METABOLIC_PROCESS**	51	0.47	1.68	0.01	0.07
		6871	0476	7274	0226
**GO_NADP_METABOLIC_PROCESS**	27	0.54	1.67	0.01	0.07
		7053	9879	6605	037
**GO_MITOTIC_SPINDLE_ORGANIZATION**	64	0.49	1.67	0.03	0.07
		0264	8947	8153	0765
**GO_GOLGI_VESICLE_TRANSPORT**	30	0.36	1.67	0.00	0.07
	7	1877	8098	3711	114
**GO_MICROBODY**	12	0.43	1.67	0.01	0.07
	3	8135	6585	7078	1747
**GO_PROTEIN_LOCALIZATION_TO_LYSOSOME**	17	0.54	1.67	0.02	0.07
		7655	5054	0408	2244
**GO_POLY_PURINE_TRACT_BINDING**	18	0.56	1.67	0.03	0.07
		4185	0002	5714	3271
**GO_CHAPERONE_BINDING**	77	0.43	1.67	0.02	0.07
		2933	2523	5048	3333
**GO_PROTEIN_DESTABILIZATION**	32	0.46	1.67	0.01	0.07
		7358	2839	9646	3366
**GO_SPLICEOSOMAL_COMPLEX**	16	0.48	1.67	0.04	0.07
	2	6543	1027	6185	3407
**GO_REGULATION_OF_TRANSLATION_IN_RESPONSE_TO_STRESS**	19	0.53	1.66	0.01	0.07
		476	9417	5564	3426
**GO_NUCLEOSIDE_TRIPHOSPHATE_BIOSYNTHETIC_PROCESS**	56	0.56	1.67	0.04	0.07
		4026	0048	0153	3427
**GO_SIGNAL_TRANSDUCTION_IN_RESPONSE_TO_DNA_DAMAGE**	91	0.41	1.67	0.00	0.07
		3897	1187	9921	3515
**GO_POSITIVE_REGULATION_OF_MRNA_3_END_PROCESSING**	16	0.56	1.67	0.02	0.07
		5645	0505	0243	3524
**GO_NUCLEAR_UBIQUITIN_LIGASE_COMPLEX**	39	0.49	1.67	0.01	0.07
		1673	0123	5094	3568
**GO_NEGATIVE_REGULATION_OF_CELL_CYCLE_PHASE_TRANSITION**	13	0.38	1.67	0.02	0.07
	6	108	1506	1484	3689
**GO_NUCLEAR_ENVELOPE**	37	0.34	1.66	0.00	0.07
	9	1029	7549	3883	4334
**GO_RNA_3_END_PROCESSING**	95	0.48	1.66	0.04	0.07
		5632	6899	5908	4491
**GO_NUCLEOBASE_CONTAINING_COMPOUND_TRANSPORT**	18	0.40	1.66	0.01	0.07
	5	4445	6038	6227	4669
**GO_POSITIVE_REGULATION_OF_CELL_CYCLE_PHASE_TRANSITION**	66	0.42	1.66	0.01	0.07
		3055	5597	9569	4785

**GO_RNA_SPLICING_VIA_TRANSESTERIFICATION_REACTIONS**	258	0.46523	1.664421	0.049505	0.0749
**GO_NEGATIVE_REGULATION_OF_MITOTIC_CELL_CYCLE**	183	0.373661	1.664885	0.023762	0.074985
**GO_POSITIVE_REGULATION_OF_DNA_TEMPLATED_TRANSCRIPTION_INITIATION**	24	0.509498	1.662848	0.015968	0.075469
**GO_POSITIVE_REGULATION_OF_CYTOKINESIS**	26	0.528476	1.662969	0.025243	0.075587
**GO_RNA_POLYMERASE_II_TRANSCRIPTION_FACTOR_COMPLEX**	96	0.408597	1.661906	0.003922	0.075742
**GO_OXIDOREDUCTASE_ACTIVITY_ACTING_ON_A_SULFUR_GROUP_OF_DONORS**	43	0.498782	1.656321	0.024209	0.078844
**GO_MITOCHONDRIAL_FUSION**	15	0.59353	1.655645	0.03125	0.07903
**GO_MYELIN_SHEATH**	161	0.433523	1.655323	0.020833	0.07904
**GO_PHOSPHOTRANSFERASE_ACTIVITY_FOR_OTHER_SUBSTITUTED_PHOSPHATE_GROUPS**	18	0.558375	1.654396	0.015534	0.079271
**GO_REGULATION_OF_TELOMERE_MAINTENANCE_VIA_TELOMERE_LENGTHENING**	49	0.479072	1.653864	0.033138	0.079432
**GO_REGULATION_OF_TELOMERE_MAINTENANCE**	64	0.457314	1.652713	0.031936	0.0799
**GO_VESICLE_COATING**	73	0.43402	1.650664	0.01444	0.080579
**GO_NEGATIVE_REGULATION_OF_CANONICAL_WNT_SIGNALING_PATHWAY**	156	0.392129	1.650949	0.013183	0.080595
**GO_MITOTIC_DNA_INTEGRITY_CHECKPOINT**	93	0.417075	1.650233	0.033932	0.080657
**GO_RRNA_TRANSCRIPTION**	17	0.570285	1.651037	0.03263	0.08072
**GO_RIBONUCLEOPROTEIN_GRANULE**	137	0.388451	1.648548	0.009901	0.081511
**GO_PIGMENT_BIOSYNTHETIC_PROCESS**	46	0.497611	1.645475	0.024048	0.082596
**GO_REGULATION_OF_DNA_BIOSYNTHETIC_PROCESS**	90	0.382535	1.646506	0.007813	0.082616
**GO_DEOXYRIBONUCLEOTIDE_METABOLIC_PROCESS**	33	0.554464	1.645628	0.047893	0.082654
**GO_ENDONUCLEASE_ACTIVITY**	115	0.414684	1.645773	0.017928	0.08276
**GO_TRANSLATION_INITIATION_FACTOR_BINDING**	27	0.52618	1.645938	0.018182	0.082819
**GO_OXIDOREDUCTION_COENZYME_METABOLIC_PROCESS**	95	0.496049	1.644715	0.035985	0.082867
**GO_SNRNA_PROCESSING**	20	0.605058	1.643927	0.027559	0.08317
**GO_NUCLEOSOME_BINDING**	44	0.482845	1.642764	0.044898	0.083271

**GO_PROTEIN_TETRAMERIZATION**	130	0.416943	1.643066	0.038685	0.083298
**GO_POSITIVE_REGULATION_OF_DNA_REPAIR**	33	0.490784	1.643328	0.015385	0.083323
**GO_REGULATION_OF_TRANSCRIPTION_FROM_RNA_POLYMERASE_I_PROMOTER**	23	0.525433	1.641021	0.02449	0.084055
**GO_TRNA_TRANSPORT**	32	0.579561	1.641259	0.043825	0.084096
**GO_REGULATION_OF_MEMBRANE_PERMEABILITY**	69	0.437475	1.640514	0.011673	0.084155
**GO_EXONUCLEASE_ACTIVITY**	70	0.466351	1.639844	0.038911	0.084391
**GO_NUCLEOTIDE_TRANSMEMBRANE_TRANSPORTER_ACTIVITY**	19	0.548533	1.636625	0.02947	0.086298
**GO_NON_CANONICAL_WNT_SIGNALING_PATHWAY**	134	0.415801	1.634467	0.015066	0.087277
**GO_TRANS_GOLGI_NETWORK_TRANSPORT_VESICLE**	27	0.479689	1.63464	0.019455	0.087356
**GO_NEGATIVE_REGULATION_OF_INTRINSIC_APOPTOTIC_SIGNALING_PATHWAY_BY_P53_CLASS_MEDIATOR**	17	0.565775	1.632703	0.039761	0.088213
**GO_GLYOXYLATE_METABOLIC_PROCESS**	27	0.548214	1.630224	0.040777	0.089745
**GO_BLASTOCYST_GROWTH**	15	0.608019	1.62853	0.021097	0.090742
**GO_TRNA_SPECIFIC_RIBONUCLEASE_ACTIVITY**	16	0.692333	1.627912	0.031311	0.090961
**GO_REGULATION_OF_GENE_EXPRESSION_EPIGENETIC**	201	0.427465	1.625431	0.041237	0.092207
**GO_PROTEIN_EXIT_FROM_ENDOPLASMIC_RETICULUM**	18	0.589426	1.624781	0.040619	0.092418
**GO_POSITIVE_REGULATION_OF_MITOTIC_CELL_CYCLE**	118	0.381924	1.622842	0.010417	0.093163
**GO_CELLULAR_AMINO_ACID_BIOSYNTHETIC_PROCESS**	86	0.421186	1.622936	0.02381	0.093304
**GO_OXIDOREDUCTASE_ACTIVITY_ACTING_ON_THE_ALDEHYDE_OR_OXO_GROUP_OF_DONORS_NAD_OR_NADP_AS_ACCEPTOR**	33	0.50746	1.622952	0.025391	0.0935
**GO_MITOCHONDRIAL_ELECTRON_TRANSPORT_CYTOCHROME_C_TO_OXYGEN**	16	0.729625	1.621491	0.040541	0.093521
**GO_REGULATION_OF_SULFUR_METABOLIC_PROCESS**	19	0.542949	1.621863	0.036364	0.093659
**GO_PRERIBOSOME_LARGE_SUBUNIT_PRECURSOR**	20	0.688506	1.621494	0.040486	0.093722
**GO_ERROR_PRONE_TRANSLESION_SYNTHESIS**	19	0.62062	1.617495	0.045098	0.095258
**GO_SPINDLE_ASSEMBLY**	67	0.45404	1.618097	0.041502	0.095281
**GO_NUCLEOTIDE_EXCISION_REPAIR_PREINCISION_COMPLEX_ASSEMBLY**	29	0.553094	1.618144	0.040936	0.095452

**GO_RNA_METHYLTRANSFERASE_ACTIVITY**	38	0.53	1.61	0.02	0.09
		3948	6384	7944	5834
**GO_POSITIVE_REGULATION_OF_DNA_METABOLIC_PROCESS**	17	0.34	1.61	0.00	0.09
	2	9259	4472	7859	68
**GO_CYTOPLASMIC_DYNEIN_COMPLEX**	15	0.59	1.61	0.03	0.09
		3045	148	2	7889
**GO_REGULATION_OF_MICROTUBULE_POLYMERIZATION_OR_DEPOLYMERIZATI**	16	0.38	1.61	0.03	0.09
**ON**	3	1388	1492	3663	8084
**GO_PURINE_CONTAINING_COMPOUND_METABOLIC_PROCESS**	37	0.40	1.60	0.03	0.09
	2	0348	9193	6538	813
**GO_ENDONUCLEASE_ACTIVITY_ACTIVE_WITH_EITHER_RIBO_OR_DEOXYRIBON**	34	0.53	1.61	0.03	0.09
**UCLEIC_ACIDS_AND_PRODUCING_5_PHOSPHOMONOESTERS**		363	1506	8462	8272
**GO_HEAT_SHOCK_PROTEIN_BINDING**	85	0.39	1.60	0.01	0.09
		811	9531	4315	8294
**GO_PEROXISOME_ORGANIZATION**	33	0.51	1.60	0.02	0.09
		4291	9764	7397	8307
**GO_TRANSFERASE_COMPLEX_TRANSFERRING_PHOSPHORUS_CONTAINING_GRO**	22	0.35	1.61	0.00	0.09
**UPS**	9	7822	0057	998	8511
**GO_TRNA_BINDING**	43	0.53	1.61	0.04	0.09
		5953	0078	9281	8709
**GO_TELOMERE_MAINTENANCE_VIA_TELOMERASE**	16	0.54	1.60	0.03	0.09
		9843	8054	5225	8799
**GO_MULTI_ORGANISM_ORGANELLE_ORGANIZATION**	23	0.57	1.60	0.04	0.09
		405	6956	7431	9188
**GO_INNER_MITOCHONDRIAL_MEMBRANE_ORGANIZATION**	16	0.66	1.60	0.02	0.09
		0344	6534	8846	9305
**GO_NAD_BINDING**	52	0.50	1.60	0.03	0.09
		2121	7018	1311	9349
**GO_REGULATION_OF_SIGNAL_TRANSDUCTION_BY_P53_CLASS_MEDIATOR**	15	0.37	1.60	0.03	0.09
	5	403	6009		9506
**GO_ANTEROGRADE_AXONAL_TRANSPORT**	22	0.49	1.59	0.03	0.10
		1234	9273	1657	2756
**GO_CYTOCHROME_COMPLEX**	19	0.67	1.59	0.04	0.10
		0925	892	8077	2763
**GO_PROTEIN_K63_LINKED_UBIQUITINATION**	34	0.44	1.59	0.01	0.10
		58	9569	5717	2957
**GO_OXIDOREDUCTASE_ACTIVITY_ACTING_ON_THE_ALDEHYDE_OR_OXO_GROU**	41	0.47	1.59	0.02	0.10
**P_OF_DONORS**		2369	9275	3438	2965
**GO_RIBOSOMAL_SMALL_SUBUNIT_ASSEMBLY**	16	0.74	1.59	0.03	0.10
		7291	7037	4351	387
**GO_MITOCHONDRIAL_ATP_SYNTHESIS_COUPLED_PROTON_TRANSPORT**	17	0.75	1.59	0.03	0.10
		7788	5468	9623	4882
**GO_POSITIVE_REGULATION_OF_TRANSLATIONAL_INITIATION**	22	0.48	1.59	0.01	0.10
		1803	2734	6	6444
**GO_SMALL_MOLECULE_BIOSYNTHETIC_PROCESS**	40	0.35	1.59	0.01	0.10
	7	8369	2895	4842	6536
**GO_ORGANELLE_TRANSPORT_ALONG_MICROTUBULE**	54	0.40	1.59	0.02	0.10
		2892	2228	5341	6617

**GO_MACROMOLECULE_TRANSMEMBRANE_TRANSPORTER_ACTIVITY**	20	0.54828	1.590717	0.036053	0.107529
**GO_RESPONSE_TO_ENDOPLASMIC_RETICULUM_STRESS**	219	0.353692	1.588754	0.013359	0.10857
**GO_PURINE_CONTAINING_COMPOUND_BIOSYNTHETIC_PROCESS**	129	0.398071	1.584256	0.039773	0.10988
**GO_REGULATION_OF_CELLULAR_AMIDE_METABOLIC_PROCESS**	318	0.328699	1.586558	0.007737	0.109885
**GO_CELLULAR_ALDEHYDE_METABOLIC_PROCESS**	77	0.459549	1.585262	0.027559	0.109953
**GO_CYTOPLASMIC_MRNA_PROCESSING_BODY**	62	0.405513	1.584676	0.028	0.109963
**GO_NEGATIVE_REGULATION_OF_CELL_CYCLE_PROCESS**	198	0.351495	1.58555	0.038835	0.109979
**GO_CUL4_RING_E3_UBIQUITIN_LIGASE_COMPLEX**	25	0.520578	1.5849	0.025948	0.110035
**GO_POSITIVE_REGULATION_OF_VIRAL_PROCESS**	84	0.39772	1.585646	0.018405	0.110131
**GO_POSITIVE_REGULATION_OF_DNA_REPLICATION**	84	0.378223	1.583523	0.012024	0.110209
**GO_DNA_TEMPLATED_TRANSCRIPTION_INITIATION**	197	0.348113	1.582562	0.013861	0.110658
**GO_DNA_DIRECTED_RNA_POLYMERASE_II_CORE_COMPLEX**	16	0.582866	1.58169	0.043643	0.110678
**GO_REGULATION_OF_DNA_TEMPLATED_TRANSCRIPTION_INITIATION**	31	0.464306	1.58169	0.021956	0.110893
**GO_G1_DNA_DAMAGE_CHECKPOINT**	68	0.412327	1.578545	0.031373	0.110959
**GO_PYRIMIDINE_NUCLEOSIDE_TRIPHOSPHATE_BIOSYNTHETIC_PROCESS**	17	0.604744	1.581735	0.035647	0.111082
**GO_PROTEIN_DISULFIDE_OXIDOREDUCTASE_ACTIVITY**	20	0.553458	1.578639	0.042718	0.111098
**GO_REGULATION_OF_MRNA_3_END_PROCESSING**	26	0.501425	1.578687	0.04375	0.111278
**GO_POSITIVE_REGULATION_OF_NUCLEAR_DIVISION**	56	0.405152	1.579055	0.015779	0.111485
**GO_ALPHA_AMINO_ACID_METABOLIC_PROCESS**	212	0.362404	1.579626	0.015936	0.111499
**GO_STRUCTURE_SPECIFIC_DNA_BINDING**	113	0.390086	1.579876	0.032587	0.111547
**GO_ORGANIC_CYCLIC_COMPOUND_CATABOLIC_PROCESS**	406	0.383136	1.580144	0.042226	0.111573
**GO_REGULATION_OF_OXIDATIVE_STRESS_INDUCED_INTRINSIC_APOPTOTIC_SIGNALING_PATHWAY**	27	0.46136	1.577089	0.033531	0.11184
**GO_METHYLTRANSFERASE_COMPLEX**	82	0.446186	1.575063	0.044898	0.112705
**GO_SPINDLE_MIDZONE**	25	0.512174	1.575182	0.047337	0.112841

**GO_REGULATION_OF_PROTEIN_COMPLEX_DISASSEMBLY**	19	0.35	1.57	0.02	0.11
	6	9446	522	4048	3023
**GO_SPERM_EGG_RECOGNITION**	37	0.46	1.57	0.03	0.11
		7515	3595	3835	3593
**GO_XENOPHAGY**	98	0.38	1.57	0.02	0.11
		4596	2667	7985	4106
**GO_REGULATION_OF_CHROMOSOME_ORGANIZATION**	26	0.34	1.57	0.02	0.11
	0	333	2262	8056	418
**GO_HSP70_PROTEIN_BINDING**	28	0.46	1.56	0.02	0.11
		286	9996	5641	48
**GO_UBIQUITIN_LIKE_PROTEIN_LIGASE_ACTIVITY**	18	0.34	1.56	0.01	0.11
	7	8939	9156	9084	4808
**GO_ORGANELLE_DISASSEMBLY**	17	0.33	1.57	0.00	0.11
	1	381	0861	947	4815
**GO_UBIQUITIN_LIKE_PROTEIN_LIGASE_BINDING**	25	0.32	1.57	0.00	0.11
	1	9673	0191	5906	4861
**GO_RESPONSE_TO_UV**	12	0.36	1.56	0.02	0.11
	0	1581	5237	2177	7417
**GO_NUCLEAR_TRANSCRIPTION_FACTOR_COMPLEX**	12	0.37	1.56	0.01	0.11
	1	2024	4304	4056	7451
**GO_TRANSFERASE_ACTIVITY_TRANSFERRING_ONE_CARBON_GROUPS**	17	0.39	1.56	0.02	0.11
	6	058	2247	7668	8544
**GO_UBIQUITIN_LIGASE_COMPLEX**	24	0.34	1.56	0.01	0.11
	6	1088	1859	7045	8657
**GO_N_TERMINAL_PROTEIN_AMINO_ACID_MODIFICATION**	24	0.46	1.55	0.04	0.12
		4133	7735	2017	136
**GO_CELLULAR_METABOLIC_COMPOUND_SALVAGE**	35	0.49	1.55	0.04	0.12
		3723	742	2991	1375
**GO_ZINC_II_ION_TRANSPORT**	25	0.48	1.55	0.03	0.12
		3189	6531	9501	187
**GO_POSITIVE_REGULATION_OF_VIRAL_GENOME_REPLICATION**	29	0.43	1.55	0.02	0.12
		285	2092	6	5053
**GO_POSITIVE_REGULATION_OF_TRANSCRIPTION_INITIATION_FROM_RNA_POLY**	17	0.53	1.54	0.03	0.12
**MERASE_II_PROMOTER**		0762	918	3074	5968
**GO_VESICLE_COAT**	44	0.44	1.54	0.03	0.12
		4668	9472	8961	6393
**GO_NEGATIVE_REGULATION_OF_SIGNAL_TRANSDUCTION_BY_P53_CLASS_MEDI**	26	0.47	1.54	0.03	0.12
**ATOR**		9489	9606	6437	6523
**GO_RNA_POLYMERASE_II_CARBOXY_TERMINAL_DOMAIN_KINASE_ACTIVITY**	16	0.57	1.54	0.03	0.12
		6145	5139	8618	8533
**GO_TRNA_METHYLATION**	20	0.59	1.54	0.04	0.13
		0148	3054	6092	002
**GO_AUTOPHAGY**	36	0.32	1.53	0.00	0.13
	0	5352	9242	9488	1854
**GO_RNA_DEPENDENT_DNA_BIOSYNTHETIC_PROCESS**	20	0.49	1.53	0.04	0.13
		6101	9402	9702	1943
**GO_POSITIVE_REGULATION_OF_CELL_CYCLE_PROCESS**	22	0.33	1.53	0.02	0.13
	4	2338	5604	7888	4446

**GO_MACROAUTOPHAGY**	25	0.32	1.52	0.01	0.13
	4	872	9517	306	7813
**GO_DICARBOXYLIC_ACID_METABOLIC_PROCESS**	97	0.40	1.52	0.03	0.13
		3592	7656	9526	8697
**GO_NEGATIVE_REGULATION_OF_ORGANELLE_ORGANIZATION**	35	0.31	1.52	0.03	0.14
	8	866	3718	0242	118
**GO_NUCLEOTIDE_TRANSPORT**	25	0.45	1.52	0.04	0.14
		7155	447	5817	1276
**GO_RESPONSE_TO_TOPOLOGICALLY_INCORRECT_PROTEIN**	15	0.36	1.51	0.04	0.14
	3	098	497	3726	7662
**GO_NEGATIVE_REGULATION_OF_CELL_CYCLE**	40	0.29	1.51	0.01	0.15
	1	6892	0761	0267	0523
**GO_NEGATIVE_REGULATION_OF_TRANSFERASE_ACTIVITY**	33	0.32	1.50	0.01	0.15
	9	3541	4471	9048	5863
**GO_METHYLATION**	22	0.35	1.50	0.04	0.15
	8	2542	1529	1833	7592
**GO_EUCHROMATIN**	30	0.44	1.50	0.03	0.15
		4211	0607	8793	8188
**GO_GOLGI_ORGANIZATION**	83	0.37	1.49	0.04	0.16
		7666	5272	2424	0686
**GO_GLUTAMATE_METABOLIC_PROCESS**	27	0.45	1.49	0.04	0.16
		0086	2286	6559	2061
**GO_ESTABLISHMENT_OF_LOCALIZATION_BY_MOVEMENT_ALONG_MICROTUBU**	90	0.35	1.48	0.02	0.16
**LE**		1294	9585	8355	3575
**GO_REGULATION_OF_MITOCHONDRION_ORGANIZATION**	19	0.34	1.48	0.02	0.16
	6	4365	6557	2989	5588
**GO_NEURAL_NUCLEUS_DEVELOPMENT**	64	0.39	1.47	0.03	0.17
		9514	7937	8745	1635
**GO_PEPTIDYL_CYSTEINE_MODIFICATION**	20	0.47	1.47	0.04	0.17
		1936	6196	9336	3124
**GO_POSITIVE_REGULATION_OF_CHROMOSOME_ORGANIZATION**	13	0.33	1.47	0.03	0.17
	9	5198	2189	6885	5594
**GO_PROTEIN_STABILIZATION**	12	0.32	1.46	0.02	0.17
	4	201	6056	2857	9514
**GO_POSITIVE_REGULATION_OF_MITOCHONDRION_ORGANIZATION**	15	0.34	1.46	0.02	0.17
	0	0057	4905	4762	9888
**GO_GTPASE_ACTIVITY**	23	0.33	1.46	0.04	0.18
	1	0929	118	381	2892
**GO_CELLULAR_PIGMENTATION**	43	0.39	1.46	0.04	0.18
		5128	1279	6729	3356
**GO_LIGASE_ACTIVITY**	37	0.30	1.44	0.02	0.19
	2	8378	9554	9412	1146
**GO_VESICLE_CYTOSKELETAL_TRAFFICKING**	36	0.38	1.44	0.04	0.19
		8189	9665	8733	1301
**GO_POSITIVE_REGULATION_OF_CELL_DIVISION**	11	0.33	1.44	0.02	0.19
	3	6159	4012	9703	5229
**GO_ORGANELLE_LOCALIZATION**	39	0.29	1.43	0.01	0.20
	1	1927	536	9157	1192

**GO_NUCLEAR_MEMBRANE**	25	0.29	1.43	0.03	0.20
	1	7656	1787	3932	3504
**GO_PROTEIN_N_TERMINUS_BINDING**	10	0.32	1.42	0.04	0.20
	1	0066	9804	7525	5258
**GO_ORGANIC_ACID_BIOSYNTHETIC_PROCESS**	24	0.30	1.41	0.03	0.21
	4	9377	1472	7736	9644
**GO_REGULATION_OF_ESTABLISHMENT_OF_PROTEIN_LOCALIZATION_TO_MITO**	11	0.32	1.40	0.03	0.22
**CHONDRION**	9	9967	5905	9474	4352
**GO_ORGANOPHOSPHATE_BIOSYNTHETIC_PROCESS**	42	0.29	1.40	0.04	0.22
	0	4173	1362	7529	8382
**GO_REGULATION_OF_INTRINSIC_APOPTOTIC_SIGNALING_PATHWAY**	13	0.31	1.39	0.04	0.23
	5	551	101	3643	7639
